# MIP diversity from *Trichoderma*: Structural considerations and transcriptional modulation during mycoparasitic association with *Fusarium solani* olive trees

**DOI:** 10.1371/journal.pone.0193760

**Published:** 2018-03-15

**Authors:** Maroua Ben Amira, Robin Mom, David Lopez, Hatem Chaar, Ali Khouaja, Valérie Pujade-Renaud, Boris Fumanal, Aurélie Gousset-Dupont, Gisèle Bronner, Philippe Label, Jean-Louis Julien, Mohamed Ali Triki, Daniel Auguin, Jean-Stéphane Venisse

**Affiliations:** 1 UCA, UMR PIAF, Clermont-Ferrand, France; 2 Faculté des Sciences de Bizerte, Zarzouna, Tunisia; 3 National Institute of Agronomy of Tunisia (INAT), Crop Improvement Laboratory, INRAT, Tunis, Tunisia; 4 National Institute of Agronomy of Tunisia (INAT), Sylvo-Pastoral Laboratory of Tabarka, Tabarka,Tunisia; 5 CIRAD, UMR AGAP, Clermont-Ferrand, France; 6 Université Clermont Auvergne, UMR CNRS 6023 Laboratoire Microorganismes: Génome et Environnement, Clermont-Ferrand, France; 7 Institut de l’Olivier, LR: Amélioration et Protection des Ressources Génétiques de l’Olivier-Université de Sfax, Sfax, Tunisia; 8 Université d’Orléans, Laboratoire de Biologie des Ligneux et des Grandes Cultures, UPRES EA 1207, INRA-USC1328, Orléans, France; Tallinn University of Technology, ESTONIA

## Abstract

Major intrinsic proteins (MIP) are characterized by a transmembrane pore-type architecture that facilitates transport across biomembranes of water and a variety of low molecular weight solutes. They are found in all parts of life, with remarkable protein diversity. Very little is known about MIP from fungi. And yet, it can legitimately be stated that MIP are pivotal molecular components in the privileged relationships fungi enjoy with plants or soil fauna in various environments. To date, MIP have never been studied in a mycoparasitism situation. In this study, the diversity, expression and functional prediction of MIP from the genus *Trichoderma* were investigated. *Trichoderma* spp. genomes have at least seven aquaporin genes. Based on a phylogenetic analysis of the translated sequences, members were assigned to the AQP, AQGP and XIP subfamilies. In *in vitro* and *in planta* assays with *T*. *harzianum* strain *Ths97*, expression analyses showed that four genes were constitutively expressed. In a mycoparasitic context with *Fusarium solani*, the causative agent of fusarium dieback on olive tree roots, these genes were up-regulated. This response is of particular interest in analyzing the MIP promoter *cis*-regulatory motifs, most of which are involved in various carbon and nitrogen metabolisms. Structural analyses provide new insights into the possible role of structural checkpoints by which these members transport water, H_2_O_2_, glycerol and, more generally, linear polyols across the membranes. Taken together, these results provide the first evidence that MIP may play a key role in *Trichoderma* mycoparasitism lifestyle.

## Introduction

Most crop farmers are confronted with the need to control various diseases (physiological or parasitic), while trying to meet strong consumer demands to use environment-friendly farming methods. One option is to use members of the fungus genus *Trichoderma*, most of which are now known to be effective antagonists of a broad array of soil-borne pathogens [1]. We recently showed that a strain of *T*. *harzianum* (*Ths97*), isolated from Tunisian farmlands, expressed antagonist activities against a strain of *Fusarium solani* (*Fso14*), which causes severe dieback of olive roots in Tunisia [2]. Fusarium root rot diseases are steadily expanding worldwide in nurseries and young olive groves, and disease control is still limited to systemic fungicide treatments and prophylactic actions. By analogy with different tripartite pathosystems that include some *Trichoderma* spp. as mycopesticides, *Ths97* is thought to act as a necrotrophic myco-hyperparasite, stopping *Fso14* growth in *in vitro* through the development of contact structures, namely helicoidal structures around its host, papilla-like structures and the collapse of several *Fso14* septa. Furthermore, on olive trees, *Ths97* develops substantial protective activity against *Fusarium* root infestation. This bioprotection is correlated with the up-regulation of an array of plant defense-related pathways by *Ths97*.

*Trichoderma* spp. occur as ubiquitous common agents in most soils, and in a few cases, they are also competitive saprotrophs, opportunistic parasites of other organisms (animals, plants or fungi), and possibly endophytes/symbionts of plants [3]. In a competitive context within an rhizospheric ecosystem, like other chemo-heterotroph mycoparasites (whether or not they are classified as a biological control agent or BCA), *Trichoderma* spp. weave an intimate network of nutritional links with their close partners, most relevantly here the plant [the olive tree] and its prey [the phytopathogen *F*. *solani*]. Even today, most studies on mycoparasites (*lato sensu*) focus exclusively on the mechanisms of attack and/or self-defense in plants [4]. Feed mechanisms are rarely mentioned or only very cursorily. Yet they are crucial to the relationship between myco-hyperparasites and other living organisms. Some aspects of this physiological pathway need to be more fully understood.

Parasite growth depends on the retrieval of a countless number and variety of nutrients from host organisms. They are mainly water, inorganic solutes, and a plethora of nitrogen and carbon organic precursors, such as carbohydrates, amino acids, fatty acids, and nucleosides. Internalizing external food, when it occurs without membrane deformation (*ie* endocytosis), is made possible by an abundant arsenal and diverse protein groups of plasma membrane transporters. This group includes the major intrinsic proteins (MIP) [5]. MIP are a large transporter superfamily generically designated as “aquaporins” (AQP). They facilitate the selective bidirectional transport of water and small uncharged molecules across biological membranes [6]. Structurally, AQP share classic folded topology and channel architecture lending them an hourglass shape. The overall three-dimensional design of the integral membrane region has a two-fold symmetry consisting of six transmembrane α-helices with five internal loops. A seventh pseudo-transmembrane helix is formed by two smaller hemi-helical segments (in the middle of loop B and loop E segments) that project opposing “NPA” boxes (Asn-Pro-Ala) at the center of the structure. The pore formed by the packing of these seven helices displays this hourglass aspect, in which the narrow constriction determines transport selectivity based on solute size and hydrophobicity [7]. A second major determinant for substrate specificity is located in the outer channel vestibule, and is referred to as the ar/R (aromatic/arginine) selectivity region [8, 9]. This feature consists of a tetrad of amino acid residues, one from each of the transmembrane helices 2 [H2] and 5 [H5], and two from the inter-helical loop containing the second “NPA” box [LE1 and LE2]. “NPA” boxes and the ar/R filter regulate the single-file conductance of water and molecules by acting as a cation- and proton-excluding selectivity filter. These physicochemical and thermodynamic contexts determine which molecules can cross the pore.

With an increasing number of genome sequences available, MIP genes have now been fully described across all living organisms, except for some thermophilic Archaea and intracellular bacteria [10]. Despite its overall diversity, the MIP family can be functionally divided into two major phylogenetic divisions, separating the water-selective AQP (*i*.*e*. the water-specific and the orthodox AQP) from the glycerol facilitators or aquaglyceroporin (GlFp) [11]. In fungi, MIP nomenclature is established on that of yeasts, and resembles that of vertebrates [12, 13]. Phylogenetic analysis finds three main groups with possible subdivisions: classical aquaporins (AQP), fungal XIP, and aquaglyceroporins (AQGP) subdivided into Fps-like AQGP, Yfl054-like AQGP and "other" aquaglyceroporins [12, 14, 15, 16].

While aquaporins have been the subject of intensive study mostly in vertebrates and plants concerning their transport specificity and their direct or indirect involvement in controlling homeostasis, their precise role in various challenged environments is still not entirely understood in most eukaryotes. This is particularly true for fungi for which MIP structure, functions and regulation are less studied, beyond several closely related models of *Saccharomyces cerevisiae* yeasts [12], two Basidiomyceta and ectomycorrhiza fungus *Glomus intraradices* [16, 17] and *Laccaria bicolor* [15], and the Ascomyceta *Aspergillus glaucus* [18]. Even so, the general lack of fungus MIP data is surprising, given the large number of fungus species and their diverse physiology and ecology niches that are always connected with water and a broad range of solutes. Remarkably, MIP from fungal mycoparasites have never been comprehensively and specifically explored.

In this study, the tripartite myco-phytopathosystem [*T*. *harzianum Ths97 –F*. *solani Fso14 –*olive trees] was used to gain insight into the molecular mechanisms involved in cell uptake of essential nutrients, by focusing specifically on the MIP route. We first investigated MIP diversity in the genus *Trichoderma*, and monitored the transcriptional expression patterns of these MIP in a situation of mycoparasitism involving the *T*. *harzianum Ths97* strain and *F*. *solani Fso14* strain, both *in vitro* and in olive trees (either preventively in primed plants or curatively in diseased plants). Second, we depicted the protein structure of the MIP expressed by modeling, and hypothesized its ability to transport water, H_2_O_2_ and glycerol. In addition, the possibility that particular solutes such as small carbohydrates may be transported across these MIP is discussed. In brief, our data provide the first comprehensive information concerning the MIP superfamily in the Ascomyceta genus *Trichoderma* and their potential involvement in a mycoparasitism context. We go on to discuss our findings with a special focus on the trophic behaviors that *Trichoderma* sets up in its habitat, which remain almost unknown in a situation of mycoparasitism.

## Materials and methods

### Fungal strains and plant material

Both the *Trichoderma harzianum* strain *Ths97* and the soil-borne *Fusarium solani* strain *Fso14* (accession number KU863548) were isolated from private Tunisian farmlands, with the permission and the help of the owner of the land, and registered at the "Institut de l’Olivier" (University of Sfax, Tunisia) [19]. Fungi were grown on PDA plates at 25°C and 27°C for *Fso14* and *Ths97*, respectively. The cultivar *Olea europaea* cv. *Chemlali* obtained from herbaceous cuttings of two-year-old plants were used for assays because of their high susceptibility to *Fso14* [20, 2]. Plants were planted in plastic bags containing autoclave-sterilized sandy clay soil, and kept in a controled growth chamber with the following growth parameters: 16h photoperiod, 26/23°C (day/night), relative humidity around 70%, and regular irrigation.

Root inoculations were performed for 1 hour by placing the roots in the conidia suspensions (S2 Fig). After inoculation, plants were replanted in plastic bags containing new soil. For the confrontation assays, *Ths97* and *Fso14* were inoculated successively with 6 days apart. The preventive assay corresponded to plants inoculated with *Ths97* in the first step, and the curative assay to plants inoculated with *Ths97* in the second step. The number of biological repetitions was: *n* = 6 for water control plants, *n* = 9 each for *Ths97* and *Fso14* infested plants, and *n* = 18 each for curative and preventive treatments. After eight weeks of infestation, roots were carefully harvested and randomly pooled in three samples in terms of biological conditions, rapidly frozen in liquid nitrogen and stored at -80°C until needed for molecular analyses. Concerning the *in vitro* confrontation tests, two mycelial plugs (8 mm diameter) were cut from the edge of actively growing cultures of *Ths97* and *Fso14* respectively, and placed 4-cm apart in a new PDA plate (S3 Fig). The paired cultures and control cultures (*Fso14* alone) were incubated at 27°C in the dark and sealed with Parafilm. The biological repetitions were done in triplicate, and each zone of interest was carefully harvested, rapidly frozen in liquid nitrogen and stored at -80°C until needed for molecular analyses. Statistical analyses of *in planta* and *in vitro* dual tests were carried out using rank-based non-parametric and ANOVA parametric methods, respectively. These analyses are detailed in [2]. All experiments for this study with the strains were done at the "Institut de l’Olivier" under the supervision of Dr TRIKI Mohamed Ali.

### Bioinformatic analysis

Protein sequences homologous to MIP transporters from *Trichoderma* spp. were retrieved at the Joint Genome Institute (http://genome.jgi-psf.org/). Some new sequences were also identified by tBLASTn searches against the NCBI GenBank GSS databases (http://www.ncbi.nlm.nih.gov/). These investigations were conducted using keyword queries (IPR000425; Major Intrinsic Protein; Aquaporin) and tblastn searches (with conservative criteria requiring a cutoff of *E*-value of 1.0^−5^). For all *in silico* analyses on *T*. *harzianum*, *T*. *harzianum* strain CBS 226.95 v1.0 (from JGI) was used as reference. Protein names and accession numbers are listed in S1 Table. During retrieval, each MIP member was verified by predicting the transmembrane topology with Interproscan from EMBL (http://www.ebi.ac.uk/Tools/pfa/iprscan/). Molecular modeling of MIP from *T*. *harzianum* was performed with the I-TASSER (Iterative Threading ASSEmbly Refinement) program suite [21, 22, 23]. Electrostatic potentials were established in a PARSE forcefield [24] using the Adaptive Poisson-Boltzmann Solver [25] in PyMOL [26], which was used to analyze and illustrate the molecular models. Structural alignment was generated with mulPBA [27]. MOLE-2 was used to define the central pores in terms of radius and polarity. Amino acid sequences were aligned using MUSCLE [28]. Phylogenetically informative positions were selected using Gblocks [29], and maximum likelihood phylogenetic reconstructions were made with PhyML (v3.0) [30] using the WAG substitution model, bootstrap supports with 500 replicates and default parameters. Tree was carried out using maximum likelihood and the phylogenetic tree was visualized with TreeDyn [31]. Theoretical isoelectric point (pI) and molecular weight (Mw) were calculated with the Compute pI/Mw tool (expasy.org/compute_pi/). Putative transcription factor binding sites (TFBSs) were analyzed on MIP genes from *T*. *harzianum* that were expressed in our biological conditions. Promoters were retrieved by searching the JGI database on sequences from *Trichoderma harzianum* CBS 226.95 that corresponded to the 1.5Kb of the genomic sequence upstream of the initiation codon. TFBSs were detected with the Promoter Database of *Saccharomyces cerevisiae*, SCPD (http://rulai.cshl.edu/SCPD/; [32]), and the putative biological processes (GO) were identified with SCPD and the Universal Protein Resource Uniprot (http://www.uniprot.org/).

### RNA isolation and quantitative real-time PCR (qRT-PCR) analysis

Total RNA was extracted as previously described by [33]. Mycelia from *in vitro* cultures and infected root tissues were ground to a fine powder in liquid nitrogen and transferred to 1 ml of lysis CTAB extraction buffer (bromide cetyltrimethylammonium). The homogenate was incubated for 5 min at 65°C, and treated twice with 1 volume of chloroform:isoamyl alcohol (24:1). The supernatant was collected and treated overnight in 2M of LiCl at -20°C. The precipitate was collected by centrifugation (16,000g for 45 min) and washed with 80% ethanol. The pellet was dissolved in 25 μl of water (DEPC) and then treated with 1U of RNase-free RQ1 DNase (Promega, Madison, WI, U.S.A.) for 30 min to remove contaminating genomic DNA. After two chloroform:isoamyl alcohol (24:1) washes, total RNA was precipitated with 100% ethanol (2V) for 2 hours at -20°C. After centrifugation at 16,000g for 30 min, the pellet was washed with 80% ethanol, dissolved in 50 μl of water (DEPC), and stored at -80°C for later analysis. RNA concentrations were determined by spectrophotometry at OD 260/280 (spectrophotometer ND-1000, Nanodrop, France), and quality was checked by using 2% TAE/agarose electrophoresis. Two μg of total RNA were reverse-transcribed into cDNA with Oligo-dT using the SuperScript^®^ III First-Strand Synthesis System for RT-PCR (Invitrogen). cDNA was diluted 10-fold with sterile water, and used as a template for qPCR. The abundance of MIP-related transcripts was determined by real-time qPCR with a MyiQ instrument (Bio-Rad). MIP gene expression levels were calculated by the 2^-ΔΔ*C*T^ method [34]. PCR amplifications were done in 15 μL of PCR reaction using MESA GREEN qPCR MasterMix Plus (Eurogentec) from 2 μl of cDNA template. Cycle parameters were 94°C for 30s, followed by 35 cycles at 94°C for 15s, at 58°C for 15s, and 72°C for 20s. PCR reactions were ended by generating a dissociation curve to confirm the amplification of PCR single bands. Geometric mean of *C*_t_ related to genes encoding to *tubulin* (*Th*, protein ID: 516507; *Fs*, 98894; JGI; [35]), *18SrRNA* (*Th*, sequence ID: KT897696.1; *Fs*, JQ837837.1; NCBI) and according to the strain, genes encoding actin (*Fs*, protein ID: 63567; JGI) or *elongation factor 1-alpha* (*Th*, protein ID: 146236; JGI; [36]) were used as internal references to normalize MIP expression for their stable constitutive expressions during fungus development and infestation. Specific primer pairs for each MIP member were designed in consensus zones after alignment of MIP sequences retrieved from *Trichoderma* spp. or *Fusarium* spp. with Primer3plus application (http://www.bioinformatics.nl/primer3plus). Specific amplification of only one desired band was observed using each primer combination for qRT-PCR analysis. Primer pairs are listed in S2 Table. All PCR technical samples were assayed in triplicate, and reactions were carried out with three biological replicates. For statistical analysis, data were analyzed using a parametric method on STATISTIX V8 software where aquaporin steady-state gene expression levels were computed by a one-way analysis of variance (ANOVA) followed by a Tukey’s honest significant difference (HSD) *post hoc* test (*p* < 0.05).

## Results and discussion

### Originality of the topic

In major ongoing research in molecular plant pathology, the characterization of virulence factors that underpin host-pathogen interactions is still a topical issue. Pathogens deploy an array of effectors that intrinsically constitute their "cell architecture" or which are secreted into the surrounding environment to interfere with host cell processes. By extension, during a situation of pathogenicity, all solute transporters may be regarded as virulence factors, since they are involved in controlling the entry into the cell of molecules with nutritive value, notably when they originate from the host prey. Because MIP play major roles in numerous physiological processes, it is meaningful to consider these channels as pathogenic factors. The genus *Trichoderma* offers us an opportunity to study this subfamily in a tripartite mycoparasitic context [*T*. *harzianum*/*F*. *solani*/olive trees] [2].

### MIP diversity

In order to characterize the MIP family from *Trichoderma*, the genome databases NCBI and JGI were searched using the previously described MIP translated sequences from *Trichoderma virens* and *Nectria haemotococca* (asexual name of *Fusarium solani*) [15]. The different *Trichoderma* strains encode six to eight predicted MIP, while *Fusarium* strains encode six predicted MIP except for *F*. *solani* with five members, and *F*. *oxysporum* and *F*. *oxysporum* f. sp. *lycopersici* with seven members each (S1 Table).

**Fig 1:**
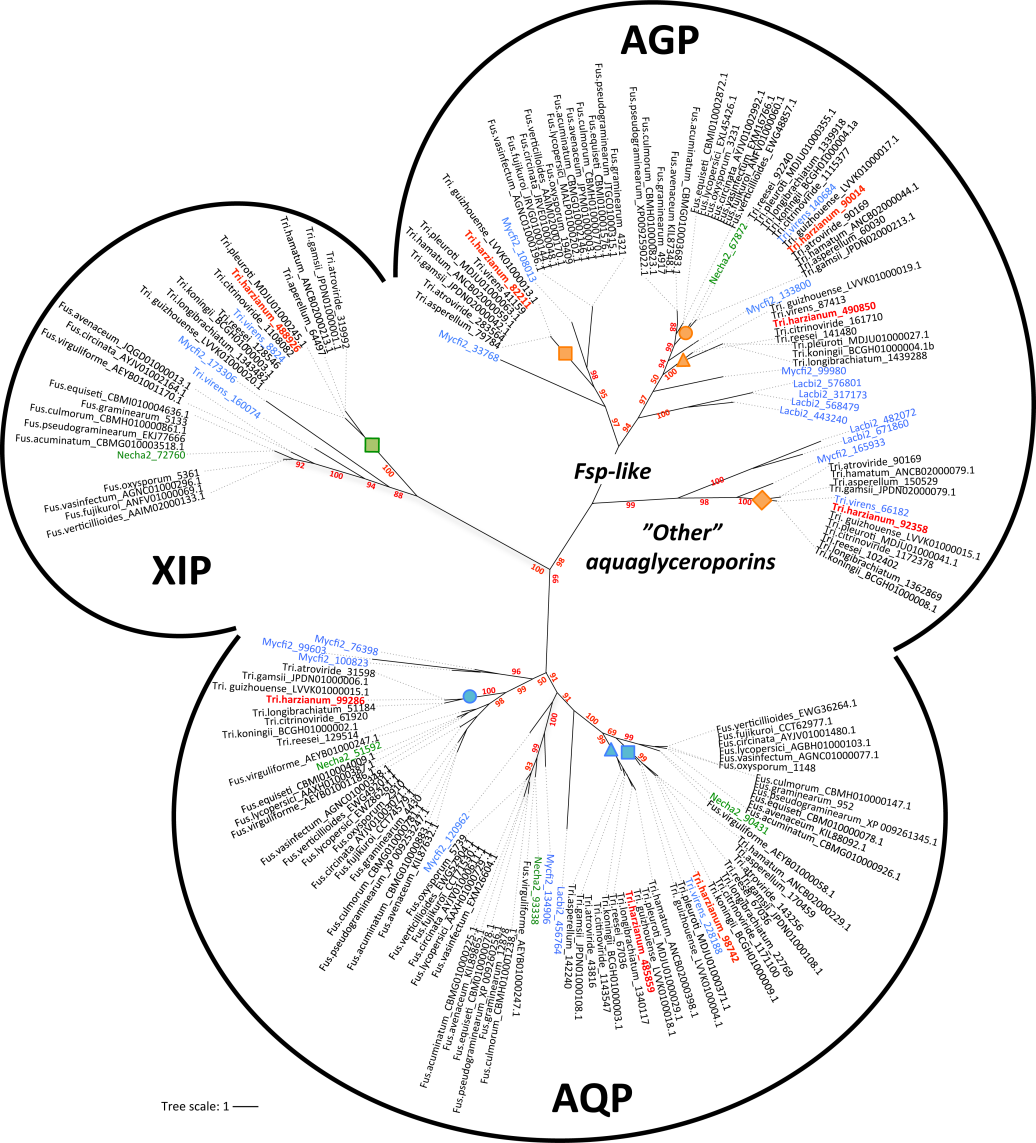
Unrooted phylogeny of MIP protein sequences from genera *Trichoderma* and *Fusarium* genus. AQP, classical aquaporins; AGP, aquaglyceroporins; XIP, X-intrinsic protein. The bootstrap values indicated at the nodes are based on 500 bootstrap replicates. Branch values lower than 50% are hidden. The distance scale denotes the evolutionary distance expressed in number of amino acid substitutions per site. MIP sequences from *T*. *harzianum* (CBS 226.95 v1.0 as reference, JGI) are highlighted in red. MIP sequences from *F*. *solani* (*Nehca2* for *Nectria haematococcae*, JGI) are highlighted in green. The reference sequences used to give the MIP sub-group nomenclatures are highlighted in blue (Lacbi2, *Laccaria bicolor* V2; Mycfi2, *Mycosphaerella fijiensis* V2; Tri.virens, *Trichoderma virens* V2; JGI). Accession numbers of proteins are attached after each species name; both are listed in S1 Table. Protein sequences are given in S1 Fig. Orange, blue and green squares, circles and triangles indicate nodes that include specific *T*. *harzianum* MIP members. This code refers to Fig 2 and S1 Table.

*T*. *harzianum* and *F*. *solani* (*Nehca2*) exhibited eight and five predicted MIP, respectively. A random analysis of this MIP family from diverse fungi (JGI) showed an average of five MIP members, placing *Trichoderma* among those species that share the broadest range. Phylogenetic relationships among the *Trichoderma* and *Fusarium* MIP proteins were analyzed with classified orthologs from *Laccaria bicolor* and *Mycosphaerella fijiensis* [15] as a reference. Sequences fall into three major clades: the classical aquaporins (AQP), aquaglyceroporins (AQGP) and X-intrinsic proteins (XIP) (Fig 1). Specifically, *Trichoderma* shows three classical AQP, three AQGP (two Fps-like and one "other" AQGP) and a single XIP. Amino acid conservation ranges from 40% to 54% sequence identity in AQP, from 40% to 54% sequence identity within AQGP, and from 76% to 87% sequence identity in XIP. By comparison, *Fusarium* exhibits three classic AQP, one AQGP (Fps-like) and likewise a single XIP. However, unlike *Fusarium*, AQP present in *Trichoderma* were split into three sub-groups, and AQGP into four sub-groups with three Fps-like and one "Other aquaglyceroporin" branches. All the XIP coalesced into a major clade, which can be divided into two branches. Although fewer subgroups are met in fungi than in plants, the emergence of a structural diversity is highlighted. Moreover, whatever the number of MIP members from each species, there is invariantly a genus-dependent subfamily distribution. Despite the common lineage of these two fungi (class of Sordariomycetes), these MIP differences in each subgroup may result from independent rounds of gene events such as duplications, but without excluding possible gene losses. For the *Trichoderma* genus, however, the limited number of differences between MIP sequences has not provided a clear-cut answer to the question of MIP expansion. At least one duplicated event seems to have occurred in *T*. *harzianum* and concerns the aquaglyceroporin 82211, absent in the ancestral species *T*. *reesei*. Gene duplication plays a key role in increasing genetic variability (driving an increase in the sizes of gene families, and *in fine*, the genome expansion of species), but most importantly, these genomic events create novel genes, which may confer potential new adaptation abilities. Here, such a relative conservation in a MIP subfamily in the *Trichoderma* genus suggests that each MIP member is devolved to transporting particular solutes that are pivotal in the full cycle of fungus development.

Additionally, insofar as these subfamilies (AQP, AGP and XIP) are expected to transport different solutes [16], the strong diversity and the large number of AQGP specifically observed in *Trichoderma* probably reflect the divergences in the adaptation of this fungi to contrasting niches and/or infection processes in a specific host range of organisms completely different from that for *Fusarium*. This differentiates *Trichoderma* from *Fusarium* in their respective mycoparasitic and necrotrophic lifestyles. While still hypothetical, it is nevertheless possible that a versatile arsenal of aquaglyceroporins may help the mycoparasite extract particular molecules at the hyphae of a broad range of potential host prey. Some examples in an amplified spectrum of genes have also been found for virulence factors (chitinases, hydrophobins, etc) in certain BCAs, which seem correlated with their strong mycoparasitic abilities [37, 38]. With the availability of the MIP gene sequences, this work lays a firm phylogenetic foundation from which to investigate this possibility by means of respective knock-out strains, and to assess possible gene regulatory network resetting linked to MIP.

## MIP structure

**Fig 2:**
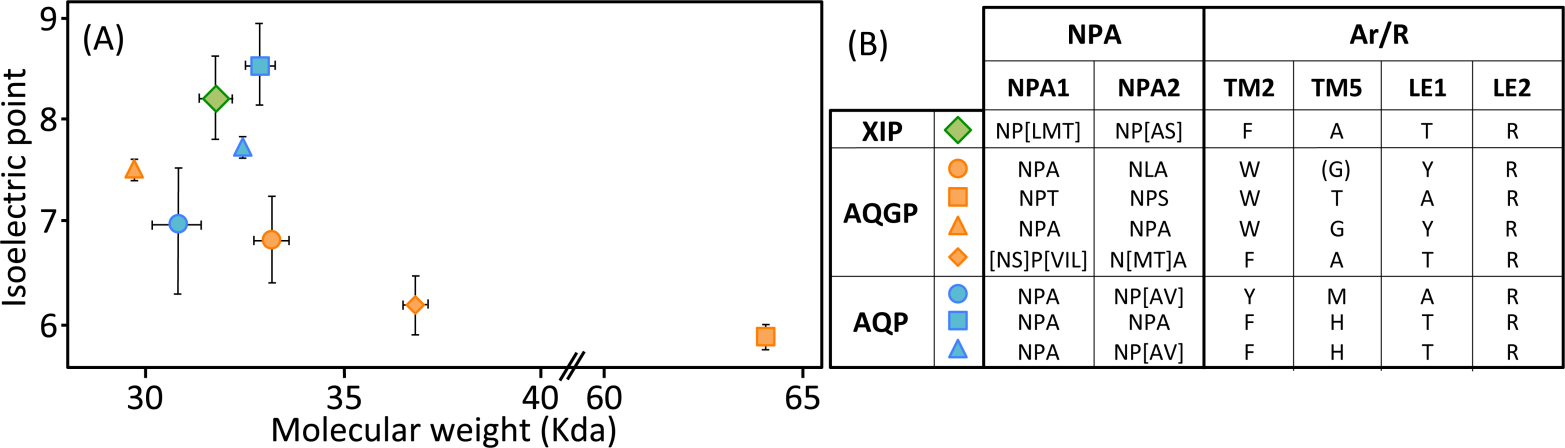
Biochemical features of *Trichoderma* MIP. (**A**) Relationship between isoelectric point and molecular weight for *Trichoderma* spp. MIP clusters. Plot showing isoelectric point versus molecular weight for XIP (X), aquaglyceroporins (X) and aquaporins (X). Subgroups are detailed in the phylogeny in Fig 1 and in the S1 Table. Means ± SE according to number of MIP members from each subgroup. (**B**) Amino acid diversity in NPA boxes and Aromatic/arginine selectivity filters in the different MIP subgroups from *Trichoderma* spp. Exact ar/R locations on MIP proteins are detailed in Figs 3A, 4A and 5A.

The MIP protein family in fungi contains a large number of highly divergent proteins. Apart from being assessed by their sequence identity, MIP diversity can be monitored not only through their biochemical features such as isoelectric points (pI) and molecular weights (MW), but also and most importantly, by modeling their three-dimensional profiles. (Fig 2; S1 Table). Except for the AQGP subgroup with Fps-like and "Other", MIP in *Trichoderma* spp. show a mean of 303 amino acids and a mean MW of 32 kDa. These features cover expected value ranges [39]. An analysis of their overall structure shows that most AQP are neutral or basic, the XIP are basic, and the majority of AQGP are neutral or acidic (Fig 2). Their distributions are in line with what has been observed for a broad range of MIP from other fungi [40].

**Fig 3:**
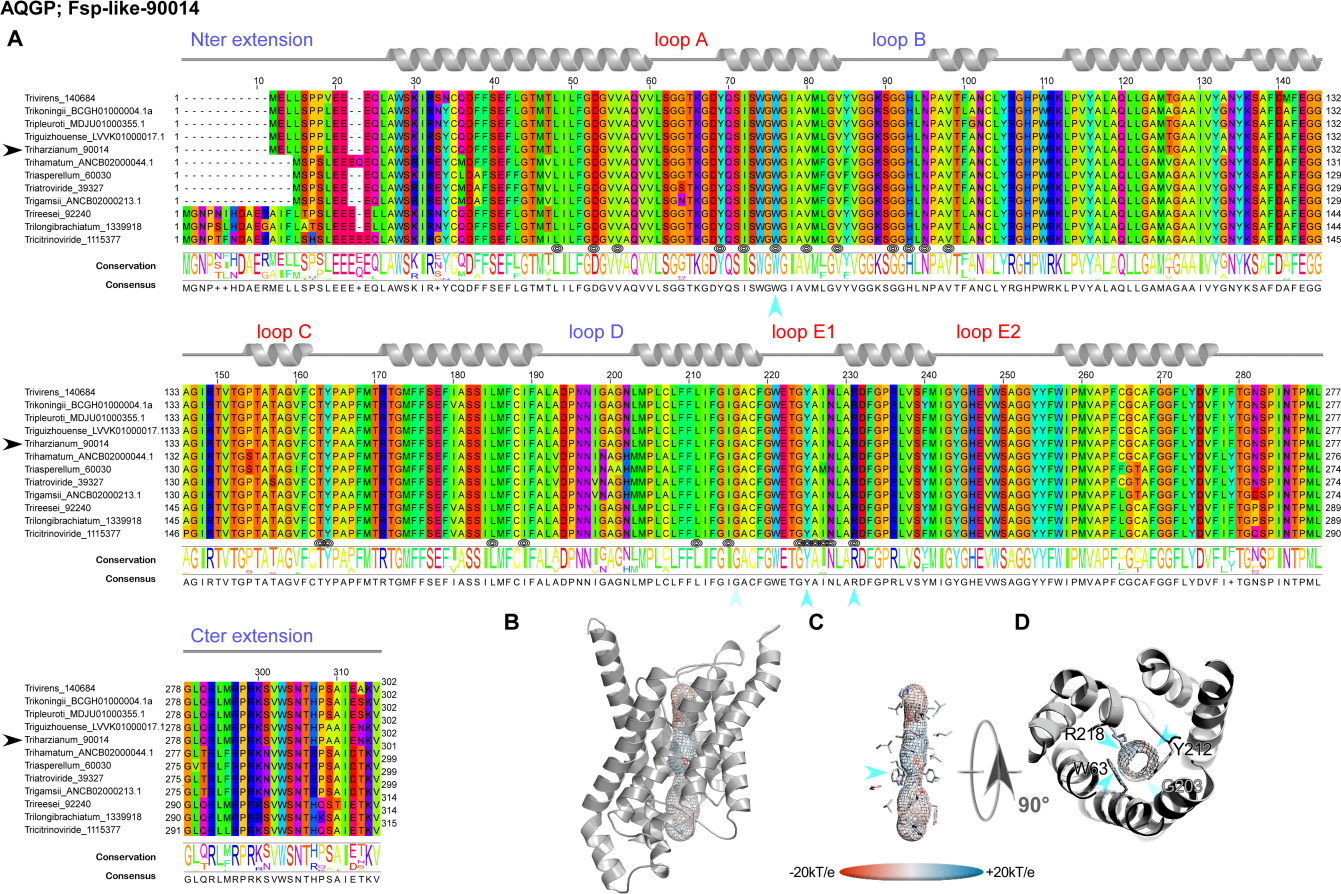
Structural analysis of the expressed fungal Fsp-like-90014 MIP. **(A)** Multiple sequence alignments (MSA) were generated from MIP homologs of different groups by group from various fungi computed with Muscle WS in Jalview, and colored by the Taylor color code. Homologous *T*. *harzianum* strains CBS 226.95 of the expressed members from *Ths97* are indicated by a black arrow before their names. Topology of each type is indicated by a ribbon diagram above the sequences on which the different segments are labeled in blue for those in the inner compartment, and red for the outer compartment. The positions of the residues exposed to the light of the channel are designated by a target symbol formed of three black circles under the MSA. The conservation and consensus sequence are given and marked by blue arrows to indicate the positions at the constriction site. (**B)** Models out of an I-Tasser computation (after different runs to improve the confidence range) are shown in PyMOL scenes. The C-score (estimating the quality of the prediction) is positive for this model used (Cscore = 1.18), suggesting a good level of confidence in all the predictions (the normal range of C-scores being between −5 and 2). The pore established with "MOLE- 2" is materialized by a grid on which the electrostatic potential calculated by APBS with the PARSE forcefield is reported to compare the physicochemical nature of the channels. (**C**) Focus on the residues of the pore. A blue arrow indicates the ar/R region. (**D**) Sidechains of the amino acids constricting the channel after both NPA motifs.

**Fig 4:**
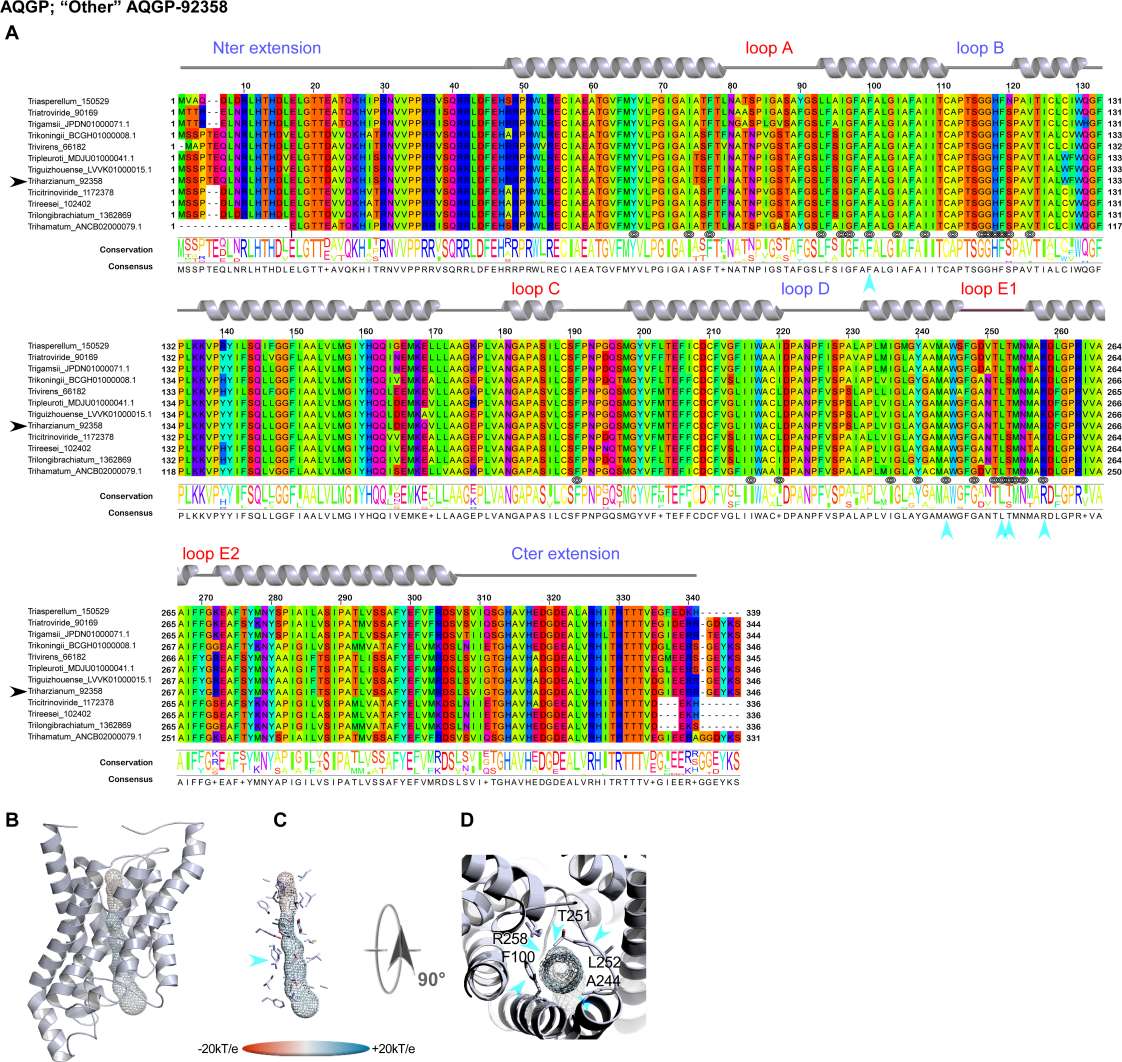
Structural analysis of the expressed fungal "other AQGP"-92358 MIP. **(A)** Multiple sequence alignments (**B)** Models out of an I-Tasser computation. The C-score (0.51) is positive, suggesting a good level of confidence in all the predictions. The pore is materialized by a grid on which the electrostatic potential is reported to compare the physicochemical nature of the channels. (**C**) Focus on the residues of the pore. A blue arrow indicates the ar/R region. (**D**) Sidechains of the amino acids constricting the channel after both NPA motifs. Technical procedures for each item are detailed in the Fig 3 caption.

**Fig 5:**
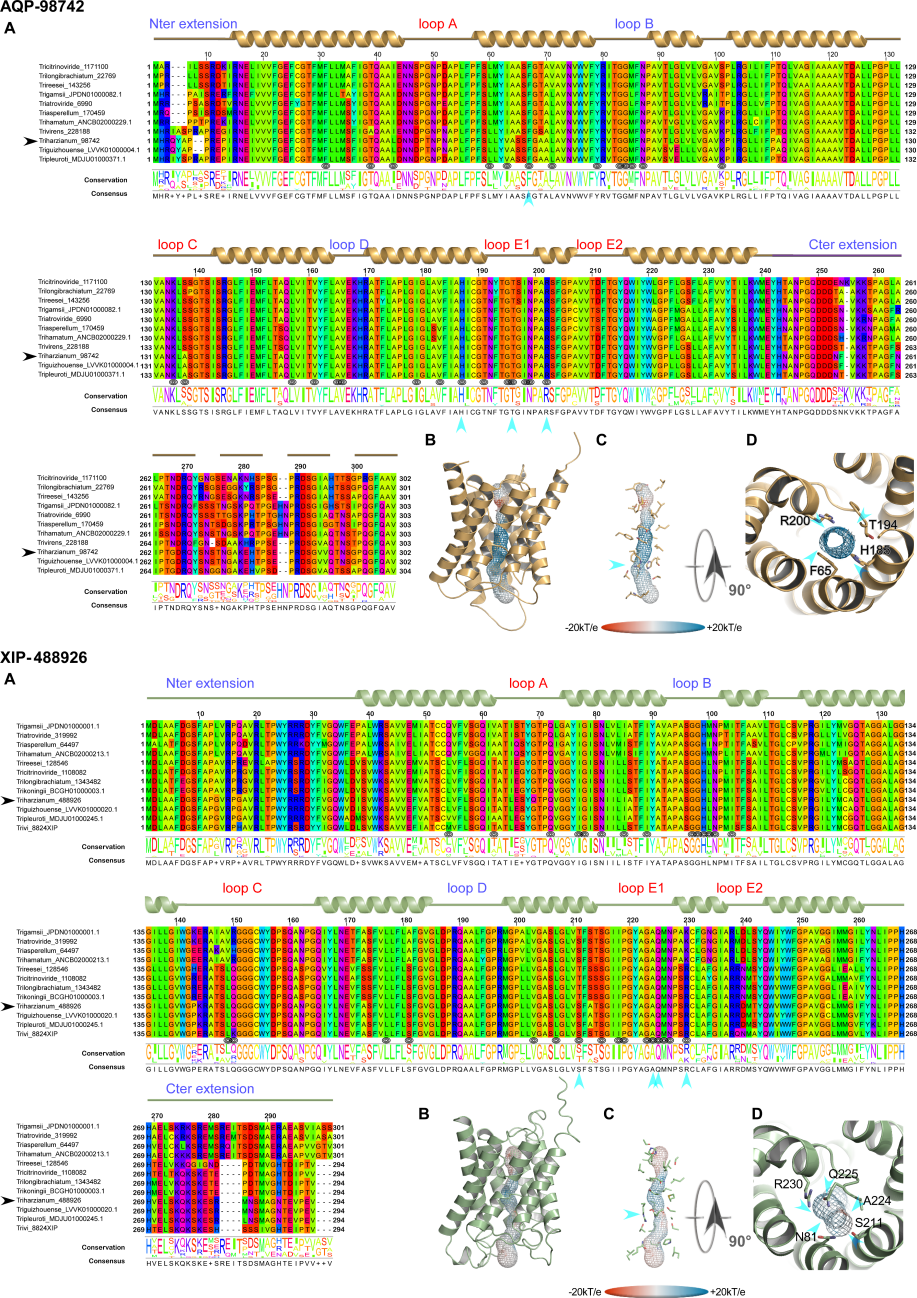
Structural analysis of the expressed fungal AQP-98742 and XIP-488926 MIPs. (**A**) Multiple sequence alignments (**B**) Models out of an I-Tasser computation. The C-score are positive for all two models used (Cscore = 1.39 for AQP-98742; Cscore = 1.26 for XIP-488926), suggesting a good level of confidence in all the predictions. The pore is materialized by a grid on which the electrostatic potential is reported to compare the physicochemical nature of the channels. (**C**) Focus on the residues of the pore. A blue arrow indicates the ar/R region. (**D**) Sidechains of the amino acids constricting the channel after both NPA motifs. Technical procedures for each item are detailed in the Fig 3 caption.

However, this analysis may be too simplistic, as these distributions do not reveal subtle features, especially as regards to potential sites of regulation such as loops and specific residues or motifs inside the pore. Further molecular structure analysis by modeling shows that the central channel polarization seems conserved, with almost the same distribution of charges along the *z*-axis. The positive charge of the guanidinium group of the characteristic arginine in the constriction region is strongly expressed, and radiates over a long portion of the light of the pore (Figs 3B, 3C, 3D, 4B, 4C, 4D, 5B, 5C and 5D) in the absence of an immediate counterion. In fact, most of the differences in size and charge of the MIP mentioned here stem from the polymorphism of their amino and carboxy terminal extensions, whose role has not yet been completely characterized (Figs 3A, 4A and 5A). We focused our interest on the MIP that are constitutively expressed (*ie* Fsp-like-90014 Fig 3; "Other AQGP"-92358 members Fig 4; AQP-98742, Fig 5; XIP-488926, Fig 5; *cf* section “MIP expression”), and inspection of the alignments by phylogenetic group shows that, aside from those variable extensions at both ends, we are facing two groups of MIP in terms of their putative transport capabilities. On one hand, we have what resembles water -and by extension H_2_O_2_- facilitators in the case of the AQP-98742 member and the XIP-488926 member, and on the other hand, we have probable glycerol facilitators in the case of the "Other AQGP"-92358 member and the Fsp-like-90014 member, whose family is also known to group glycerol facilitators regulated by osmotic changes [16]. This segregation is confirmed on a Newick tree when comparing the four models on multiple structural alignments with MulPBA [27]. This could be extended to the other members of each subfamily or group considered here because of their intra-proximity. The primary difference justifying this segregation is located at the principal constriction site in the central pore, the so-called ar/R filter, which is slightly smaller in the water-specific AQP and composed of four residues, and slightly larger in the aquaglyceroporin and composed of four residues of which one is small (alanine) or by only three residues (the fourth is absent, and instead a glycine is found in its position). In our case, the constriction site is composed of F65, H185, T194 and R200 for the AQP-98742 member, of N81, S211, Q225 and R230 for the XIP member, of F100, A244, T251 and R258 for the "other AQGP"-92358 member, and W63, Y212, and R218 for the Fsp-like-90014 member (Figs 2, 3A, 4A and 5A). The associated diameters measured at this site on our models with the MOLE 2.0 approach were 1.28Å, 1.8Å, 3.78Å and 2.54Å, respectively, which is consistent with the reasonable assumption that the aperture in the glycerol transporters will be larger than in strict water transporters.

The second difference arises from the extracellular loops (A, C and E), which present variable lengths. Loop A with 14 residues (D42-P55) is prominent as expected for the AQP-98742 member. This is also found on the "other AQGP" member, where it measures 13 residues (N80-S92); loop A is found to be slightly shorter with 10 residues (L65-G74) for the XIP, and is substantially halved with 7 residues (S49-D55) for the Fsp-like-90014 member. This criterion does not seem to be discriminating in terms of molecules to be transported. Conversely and more remarkably, loops C and E seem to permit a distinction in the nature of the transport ensured by the MIP, suggesting a possible coupling with a third-party effector, as it could provide an interacting site for one. We note that both putative glycerol facilitators share a common topology concerning their long loop C, which fits the model of an alpha hairpin as found in the GlFp, for which the archetype namely the *E*. *coli* Glycerol Facilitator structure was released [41]. The second alpha helix of the hairpin is mostly hydrophobic and ends with a cysteine, which is also found in the XIP member at the same position near the pore entry. In both putative glycerol transporters, this segment is 20 residues longer than its homolog in the AQP-98742 fold: loop C is 19 residues long in the AQP member, 24 residues in the XIP member, 38 residues in the "other AQGP" member, and 36 residues in the Fsp-like-90014 member. On the intracellular side, we note a last subtle but still remarkable difference between the two GlFp candidates expressed concerning the net charge of loop B. In the first segment of this long loop, prior to the short NPA helix and at the very beginning of the loop, a lysine conserved in the Fsp-like subgroup (K76) is found instead of a conserved threonine (T114) as in the "other AQGP" members. *Post hoc*, the characteristic asparagine residue of the NPA motif is replaced by a serine in most of the "Other AQGP" members. In the second part of this loop, a conserved arginine (R93) is present in the Fsp-members, while this position is occupied by a glutamine (Q131) in most of the "other AQGP" members. This loop ends in both groups in a basic motif of two successive lysines in the "other AQGP" and an arginine followed by a lysine in the Fsp-members. To sum up, this loop is more basic in the Fsp candidates in addition to the presence of a supernumerary and conditional positive charge of a histidine (H95 instead of the neutral F133). This could have implications in how the two kinds of pores function, for example in their ability to interact with possible regulators, or in their ability to favor one circulating direction for the polyols they can tunnel across the membrane. The impact of such a subtle difference will need to be addressed in further investigations.

**Fig 6:**
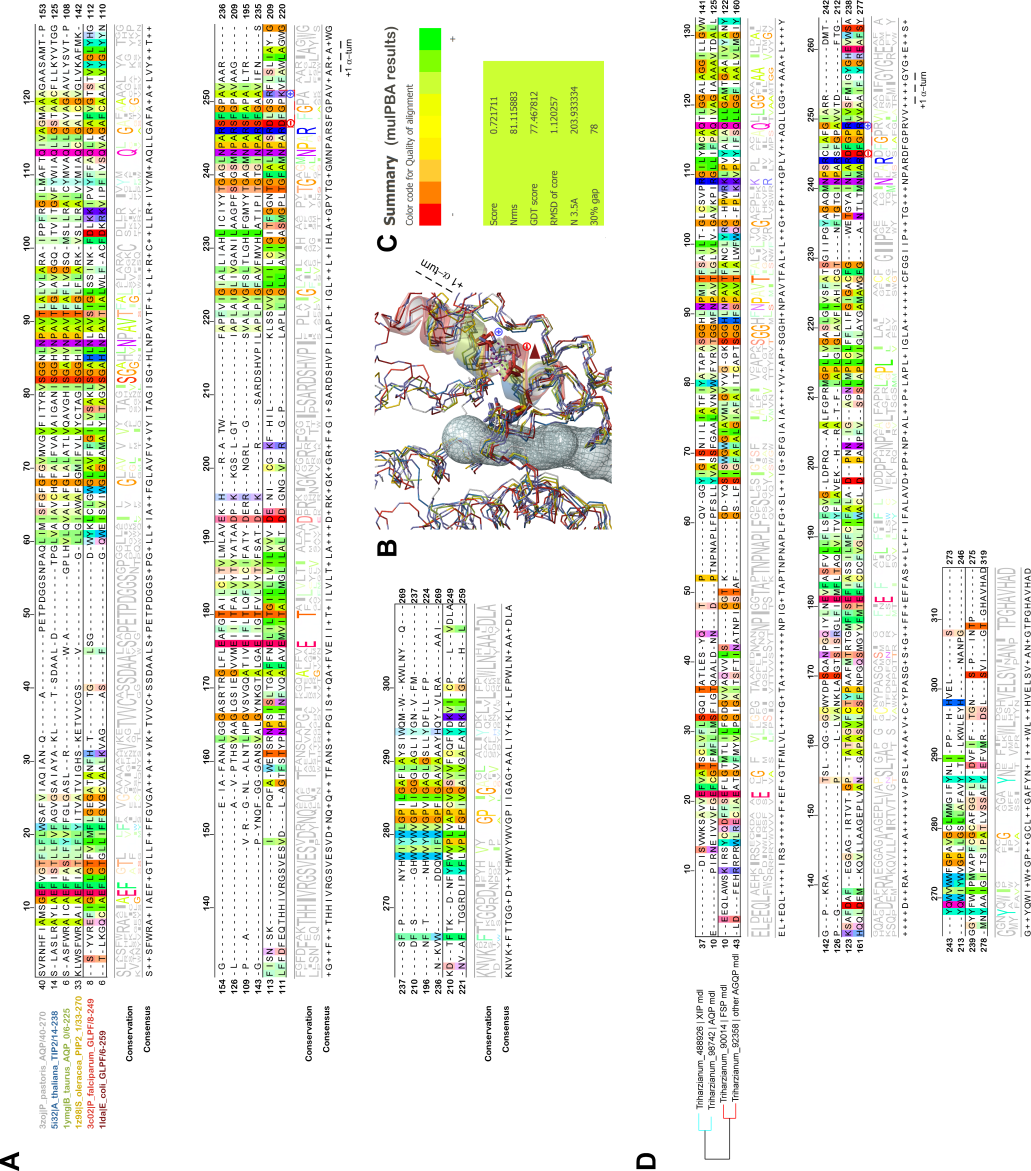
Structural alignments of MIP to highlight noticeable differences in glycerol facilitators *versus* standard AQP. **(A)** Structural alignment of different MIP based on the coordinates of resolved structures made with MulPBA on a narrow but still representative sample of MIP of different classes from different kingdoms. The name of the proteins and their relative pdb code is written with distinctive colors on the left of the alignment, itself colored by the Taylor color code in Jalview. A conservation threshold of 50% is applied to highlight the conservation by groups. From this comparison emerges the particular meaning of the conserved GlFp motif NPARD: the conserved negatively-charged residue aspartate makes a salt bridge with an equally conserved residue at exactly one α-turn from it. This bridge quenches both charges by mutual neutralization, allowing their presence in a quite hydrophobic environment for folding purposes (first quarter of α-6). **(B)** PyMOL scene of the superimposition results from mulPBA displayed as a wireframe diagram of the main chain colored with respect to the sequence name coloring. The channel is shown as a transparent volume to materialize the localization. The sidechain of the conserved arginine from the NPAR motif is shown as sticks, as also are both charged residues occurring only in the GlFp proteins (light and dark red). A red arrow shows the relative displacement (concomitant with this type of electrostatic bridge within the short α-helix of loop E) responsible, at least in part, for a larger pore aperture at its constriction site. Only the NPA α-helices are shown as transparent colored coils (**C)** Summary of the superimposition score from mulPBA. **(D)** Structural alignment of MIP from the *T*. *harzianum* strain CBS 226.95 homologous to those expressed from *Ths97* and based on the coordinates of good quality I-Tasser homology models. The MSA is colored by the Taylor color code in Jalview. On the left, the Newick tree established by mulPBA is given showing the relative proximity of both XIP-488926 and AQP-98742 members on one side, and both "other AQGP"-92358 and Fsp-like 90014 members on the other side. Models are consistent with previous data obtained on experimental structures. A conservation threshold of 50% is also applied to highlight the conservation by groups.

Even more interesting, in both GlFp-like proteins we found an internal salt bridge between the conserved aspartate next to the ar/R filter arginine with another arginine on the helical turn immediately following, possibly helping to regulate aperture size by tilting the short NPA helix (Fig 6). This also occurs in generic GlFp, where this hemihelix is also one turn longer than its homolog from the AQP-98742 member. This is currently apparent in two available structures, an aquaglyceroporin from *Plasmodium falciparum* (pdb code 3C02) and the first in the series of the *E*. *coli* glycerol facilitator (pdb code 1FX8). It implies translocation of the arginine of the filter, and its Cbeta moves about 1Å away from its canonical position on classical aquaporins. This *in silico* data can provide intuitive insight into the potential permeability properties of the channel in transporting not only polyhydroxyl alcohols (or polyols such as glycerol), but also more voluminous polyols such as erythritol, arabitol, sorbitol and mannitol as observed for *pf*AQP and *Ap*AQP2, two multifunctional aquaglyceroporin channels from *Plasmodium falciparum* and *Acyrthosiphon pisum*, respectively [42, 43]. These polyol transporters, alongside specific sucrose transporters, could be expected to feed the fungal carbohydrate metabolism, which provides energy for hyphal growth and supplies carbon skeleton to other metabolisms. However, and again most importantly, they may participate in the continuous process of generating hydrostatic pressure used by the pathogenic hyphae to break the hyphae cell wall surface of its host and penetrate it. Because polyols make a major contribution to the osmotic ballast, water and polyols are two interplaying components essential for hyphae integrity when fungi move in fluctuating environments [42, 15, 16].

Finally, we used the Glycopred prediction method to examine the differences in terms of numbers of potential glycosylation sites in these external loops. All of them are potentially glycosylated except for loop A of the FSP member, loop C of the AQP and loop E of the XIP and both putative glycerol facilitators. Most of the sites are far from the central pore. In the putative glycerol facilitators, glycosylation sites are found in the descending hydrophobic helix of loop C.

To conclude, on the basis of these structural and possible functional considerations, elucidating the physiological role of MIP in *Trichoderma* spp. through in-depth functional studies with MIP variants in key residues will answer these important unanswered questions. However, this approach will not be applied on *Ths97*: systematic of *T*. *harzianum* appears to be complex with many cryptic species, making it quite difficult to work with. Mutagenesis technologies require double cross-over homologous recombination around 1,5kb up- and downstream of the target gene, and therefore a thorough knowledge of intergenic regions, which are highly diversified and complex between *T*. *harzianum* spp. in contrast to the transcribed regions, which are highly conserved as shown by *MIP* genes. Thus, our hypothesis will need to be confirmed in the future by mutagenesis of *MIP* from *Trichoderma* species whose genomes are sequenced.

## MIP expression

**Fig 7:**
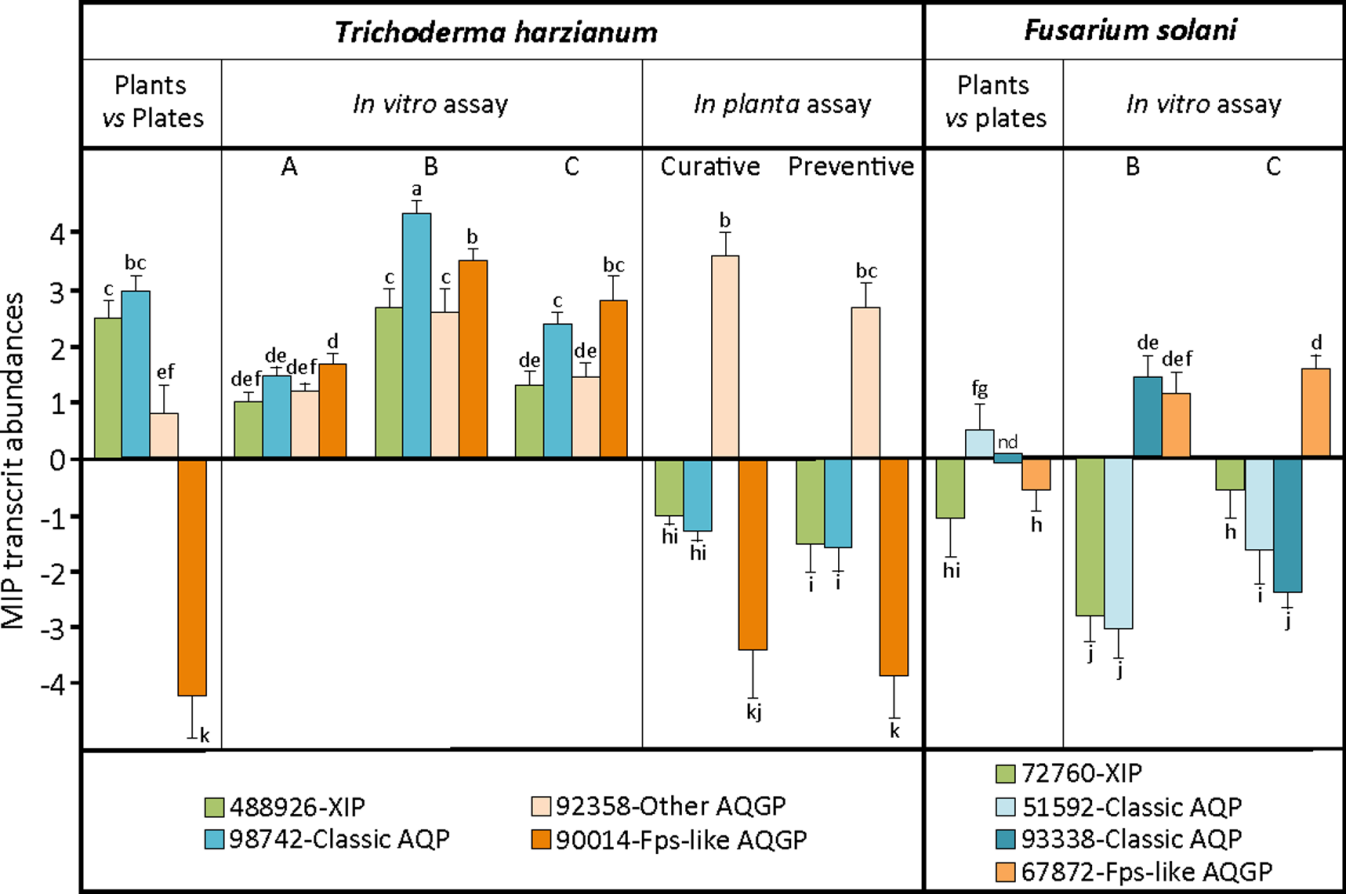
Relative transcription ratios of the MIP genes from *T*. *harzianum* and *F*. *solani*. Relative expression levels of MIP genes from *T*. *harzianum* (*Ths97*) and *F*. *solani* (*Fso14*) cultivated separately or together in artificial culture (after 6 days of inoculation) or on roots of olive trees (after 8 weeks of inoculation). **Plants *vs* Plates**: constitutive steady state level of MIP expression from *T*. *harzianum* and *F*. *solani* cultivated separately in plants or on PDA medium. ***In vitro* assay**: (A) *T*. *harzianum* individually, (B) area of confrontation between hyphae, (C) area of overlap of *T*. *harzianum* on *F*. *solani* (detailed in S2 Fig). ***In planta* assay**: inoculated separately in roots or in curative and preventive contexts (root symptoms detailed in S3 Fig). Transcript levels for each gene were estimated using real-time qRT-PCR analyses, and normalized by the expression of three housekeeping genes specific for each fungal strain. Relative transcript abundance rates were obtained by the *E*^-ΔΔC*t*^ method with transcript abundances from individual *in vitro* culture or *in planta* inoculation. Data correspond to means of three independent biological experiments. Bars represent the biological standard error. Data not sharing the same letter are significantly different (Tukey *post-hoc* test after one-way analysis of variance (ANOVA), *p* < 0.05). Nd, transcript non-detectable significantly.

The transcriptome of *Trichoderma* is still the subject of several molecular studies, leading to the identification of pathways involved in the different aspects of biocontrol mechanisms [1, 44, 45, 46]. From these studies, however, no MIP information has yet been provided. To complete the *in silico* identification of candidates for MIP channels, their expression profile was addressed at transcript level using real-time quantitative reverse transcription-polymerase chain reaction (qRT-PCR) with MIP gene-specific primers. Molecular analysis is aimed primarily at *Trichoderma* under non-mycoparasitic conditions (mycelial growth or infestation without its host *F*. *solani*, corresponding to the control samples) or under mycoparasitic conditions in the presence of *F*. *solani* (corresponding to the assays). Additionally, two different biological contexts were studied: on “artificial substrates” with PDA on Petri dishes (*in vitro*), and in roots from young olive trees (*in planta*). Similarly, MIP expressions from *F*. *solani* were studied in the same biological conditions. Results demonstrated that of the eight MIP genes present in the genome of *T*. *harzianum*, only four were transcribed with significant differential modulation during mycelial growth on an artificial medium and on olive roots (Fig 7). In detail, the steady-state level of transcript abundance of AQP-98742, AQGP-92358 and XIP-488926 was higher during the mycelial growth *in planta* than *in vitro*, while AQGP-90014 was slightly less abundant. These diverging expressions between “growth environments” are not surprising and have already been mentioned [47]. They could result from the presence of various chemical factors in plant tissues that may be lacking in artificial substrates. Similarly, these contrasting expressions could be linked to a subtle difference observed between the net charges of their respective loop B that would determine a specific ability to favor one circulating direction of particular solutes across the membrane.

Concerning the confrontation situations, and irrespective of the biological contexts (*i*.*e*. preventive or curative), the expressions of these four MIP were significantly modulated by the presence of *F*. *solani*. It is of note that the onset of a differential expression of MIP genes is a rather early event during the interaction with host prey: it occurs during the first stage of mycelial growth, when *T*. *harzianum* is in physical contact with its prey. MIP transcript abundance then increased considerably over the contact area between *T*. *harzianum* and *F*. *solani*.

Unexpectedly, MIP patterns contrasted sharply between the artificial and *in planta* dual culture contexts, except for AQGP-92358, which remained up-regulated. AQP-98742, AQGP-90014 and XIP-488926 were up-regulated in artificial substrates, but down-regulated *in planta*. Very few functional studies have been carried out on fungal MIP. However, AQP-98742 belongs to a MIP cluster that comprises MIP with putative water channels [12, 14, 47]. AQGP-90014 belongs to a "facultative Fsp-like aquaglyceroporin" cluster including MIP with putative water, glycerol and small neutral molecule transport channels [13, 17, 48], whereas AQGP-92358 belongs to the "other-aquaglyceroporin" cluster made up of MIP that present glycerol and small neutral molecule transport capacities [13, 15]. Concerning XIP, no biochemical validation has been reported in fungi. However, three inputs can be exploited to gain a better understanding of this unorthodox cluster: (i) the MIP JQ412059 from *Glomus intraradices*, a relatively proximate phylogenetic homolog of fungal XIP [16], exhibits a water transport channel [49], (ii) its transcriptional kinetics parallel that of AQP-98742 (Fig 7), and (iii) three-dimensional structure analysis suggests a tighter channel, particularly at the constriction zone approaching the level seen in the AQP-98742 channel (Fig 5). This would indicate a plausible ability to channel water and possibly other small polar molecules like H_2_O_2_, but not glycerol as previously observed for certain XIP from plants [50, 51, 52]. Despite these putative biochemical extrapolations, and because evidence of how MIP take part in fungal lifestyles is still scant and speculative, further interpretations concerning the putative involvement of each member during mycoparasitism of *Ths97* would be premature. However, data do suggest that *F*. *solani* has a direct influence on *Ths97* genome reprogramming, and this is significant when we read the MIP expression from the ‘*in planta*’ biological context. This takes place invariantly whether *Ths97* has colonized healthy plant tissues prior to a *F*. *solani* infestation (preventive treatment) or an infested fusarium environment (curative treatment). The *in vitro* and *in planta* environments are not comparable, and it is difficult to envisage how *F*. *solani* can directly up- or down-regulate some *Ths97* genes in specific environments, unless we consider the possibility that *Ths97* displays a direct mycoparasitic activity on *F*. *solani*. The interaction of *T*. *harzianum* with *F*. *solani* is described as mycoparasitic [35], and this feature was observed *in vitro* between *Ths97* and *Fso14* (S2 and S3 Figs) [2]. This overall adjustment may be supplemented by the release of cell-wall-degrading enzymes, known to be directly involved in the mycoparasitism interaction, and whose production is influenced by various ambient factors [53, 54, 55]. These fine and complex molecular adjustments generate specific metabolized-products (*i*.*e*. oligomers) that may themselves become secondary inducers of cell responses for *Trichoderma* [56, 57]. This would explain the differential expression patterns of transcripts encoding MIP proteins observed during the different biological contexts and stages of confrontation.

Two other interesting scenarios should be considered. The first one is that the biochemical environments of the intercellular space change fundamentally. This event is mainly due to the virulent activity of *F*. *solani* and also its ability to secrete an arsenal of hydrolytic enzymes [58, 59]. Certain particular plant residues generated by *F*. *solani* could interfere here with *Ths97* cellular responses. Such residues are inevitably absent in the *in vitro* context, but could possibly be produced when *F*. *solani* infests its plant host. In the second scenario, although no information is available about competition and defense reactions of *F*. *solani* as a host, *F*. *solani* would is be able to develop a differential toxicogenic activity *in planta* compared with the *in vitro* context (like *Ths97*, *F*. *solani* senses and responds differentially to contrasting environments) [60, 61], and specific secreted mycotoxins (possibly in a "growth medium"-dependent manner) could affect certain gene responses in *Ths97* without, however, upsetting its mycoparasitic behavior. To the best of our knowledge, there is no evidence to support these two last suppositions, but whatever the case, the transport machinery reprogramming for *Ths97* is governed by environmental changes, probably due to the presence of exudates released from the host mycelium (*F*. *solani*), whose priority remains to meet nutritional needs. As for *F*. *solani* MIP expression patterns, four MIP out of the six in its genome were transcribed and differentially modulated. Interestingly, none of them was significantly detectable in infested plants treated with *Ths97*. This result provides new evidence suggesting the ability of the beneficial partner to drastically reduce the population of its host target.

### MIP regulation

In line with previous findings, we showed here that *Ths97* seems able to sense the presence of its host prey and respond by modulating a set of genes that could be involved in its mycoparasitism. In our work on MIP, we are aware that correlations alone do not allow a causal link to be established. In addition, the transcriptional level does not represent what happens at the protein level. However, there are good indications that MIP transcript regulations may imply assigned functions of isoforms in mycelia trans-cellular solute flows. Thus whatever the biological contexts, we can intuitively expect the expression of a broad range of genes to depend preponderantly on solute sources (carbon, nitrogen, minerals, etc) available in the environment. One of the major challenges facing biologists is to unravel the complex networks that govern these gene expressions. One clue could come from the establishment of the existence of metabolons. Transcriptional regulation relies to a large extent on molecular mechanisms that allow nucleic acid binding proteins or transcription factors (TF) to recognize specific sets of nucleic acids in DNA, known as transcription factor binding sites (TFBSs) or *cis*-regulatory sequences. Identifying these regulatory elements in non-coding regions is an interesting key step in understanding gene regulation and ultimately in inferring regulatory networks.

We scanned 1.5kb upstream of the start codon of the four expressed MIP using the yeast *Saccharomyces cerevisiae* dedicated promoter database SCPD [32]. Conscious of the limitations inherent in such a systematic analysis on TFBSs, which are usually very short and statistically often highly degenerate, the fact remains that results showed an over-representation of motifs targeted by TFs known to be involved in various carbon, nitrogen sulfur and phosphate metabolic processes (Tables 1 and S3). Between 62% and 81% of motifs constituting the four MIP promoters are in promoters of genes encoding proteins involved in carbohydrate, fatty acid and sterol, amino acid or nucleotide metabolisms. Unexpectedly, motifs involved in the cellular responses to stress (osmosensing and ion homeostasis pathways, drug metabolization and exportation, oxidative stress) were poorly represented (<0.05%). This contrasts notably with plant MIP promoters, which contain a large number of TFBSs related to cellular responses to abiotic and biotic stress [62, 63, 64]. The remaining motifs control mRNA transcription, cell growth and division, and DNA synthesis (meiosis process) (S3 Table). We hypothesize that this *cis*-element provides indications about the potential involvement of these MIP in establishing a trophic relationship that *Trichoderma* creates with its surroundings, and especially here with *F*. *solani*, with which *Ths97* initiates a competitive relationship. This functional trend corroborates previous findings where functional annotations of different wide-transcript libraries linked to a mycoparasitic context indicated a substantial over-represented category related to various metabolic processes [35, 53, 55, 65]. Finally, if *F*. *solani* really influences *Ths97* genes expression in some way, then it would be relevant to identify the TFs network from *Ths97*, which could be in direct relation with certain virulence effectors secreted by *F*. *solani* during its plant tissue infestation phase, or related metabolized products in the case of effectors with intrinsic hydrolase activities. To further test our hypothesis of a plausible involvement of MIP in the competition machinery of *Trichoderma* against various pathogens, and the establishment of its trophic network, suppression of MIP gene function within non-encoding and encoding regions would have to be addressed in future experiments.

**Table 1:** Proportion of putative transcription factor binding sites (TFBSs) on the 1.5kb promoter region of the expressed MIP genes from *Trichoderma harzianum*. MIP promoter sequences from *T*. *harzianum* CBS 226.95 v1.0 (JGI) were used as reference. TFBSs were detected with the "Promoter Database of *Saccharomyces cerevisiae*" (http://rulai.cshl.edu/SCPD/), and biological processes (GO) analyzed using «Uniprot» (http://www.uniprot.org/). TFBS nucleotide sites on 1.5kb of each promoter are detailed in the S3 Table.

Cis-motif	UniProt identifier	XIP 488926	AQP 98742	AQGP 92358	AQGP 90014	
ADR1	P07248	4 (3,8%)	2 (1,8%)	4 (3,3%)	4 (3,6%)	Carbon and nitrogen metabolic processes
BAS2	P07269	1 (0,9%)	7 (6,1%)	1 (0,8%)	0	
CSRE	-	1 (0,9%)	1 (0,9%)	1 (0,8%)	0	
GAL4	P04386	2 (1,9%)	0	0	0	
GCN4	P03069	20 (14,4%)	12 (10,5%)	16 (13%)	9 (8,1%)	
GCR1	P07261	9 (8,7%)	12 (10,5%)	18 (14,6%)	21 (18,9%)	
LEU3	P08638	1 (0,9%)	1 (0,9%)	0	0	
MCM1	P11746	1 (0,9%)	2 (1,8%)	2 (1,6%)	2 (1,8%)	
MIG1	P27705	1 (0,9%)	1 (0,9%)	6 (4,9%)	3 (2,7%)	
PUT3	P25502	4 (3,8%)	0	0	0	
CAR1 Repressor	P39001	15 (14,4%)	12 (10,5%)	19 (15,4%)	13 (11,7%)	
RAP1	P11938	2 (1,9%)	1 (0,9%)	1 (0,8%)	0	
STE12	Q03063	4 (3,8%)	4 (3,5%)	6 (4,9%)	8 (7,2%)	
PHO2	P07269	12 (11,9%)	12 (10,5%)	13 (10,6%)	8 (7,2%)	Phosphate transport and metabolic processes
PHO4	P07270	4 (3,8%)	4 (3,5%)	13 (10,6%)	4 (3,6%)	
		**81/104 (77%)**	**71/114 (62%)**	**100/123 (81%)**	**73/114 (64%)**	
PDR1-PDR3	P33302	0	0	5 (4,1%)	0	Cellular cation homeostasis / xenobiotic transport
ROX1	P25042	2 (1,9%)	1 (0,9%)	0	1 (0,9%)	Cellular response to osmotic stress
RML1	Q12224	0	1 (0,9%)	0	0	Cellular response to stress
SMP1	P38128	1 (0,9%)	1 (0,9%)	0	0	Cellular response to stress
XBP1	P39001	1 (0,9%)	1 (0,9%)	1 (0,8%)	3 (2,7%)	Cellular response to oxidative stress
		**4/104 (0,04%)**	**4/107 (0,04%)**	**6/123 (0,05%)**	**4/114 (0,04%)**	

## Conclusion

Our present results bring us nearer to understanding one molecular mechanism potentially involved in the mycoparasitic process of *T*. *harzianum* (with the example of *Ths97* here) with the involvement of the MIP family. Modulated transcript abundance of members belonging to the three sub-classes representative of the fungal MIP family suggests the importance of transporting certain specific solutes during hyphae development and possibly self-defenses. However, owing to the complexity of the underlying mycoparasitism mechanisms, an in-depth understanding of the functional characterization of the MIP genes reported here is essential, and this will be improved by future studies of their subcellular localization, post-translational regulation and precise roles in signaling and solute transporting processes in such "myco-phytoparasitic" tripartite interactions. Part of this effort will require focusing on key residues shown in this study to be responsible for the specialization of the two GlFp and subsequently testing these by mutagenesis approaches. Lastly, if we consider -by definition- that MIP are virulent factors in this (myco)parasitic interaction, the manipulation of candidate MIP genes linked to virulence activity remains a pertinent approach to improve the *T*. *harzianum* strain.

## Supporting information

S1 Fig Detail of all MIP protein sequences used in this work. (PDF)

S2 Fig Symptoms of fusarium root rot disease on root system from olive trees. Preventive treatment: *Ths97*-treated plants subject to *Fso14* infestation; Curative treatment: *Fso14* infested plants treated with *Ths97*. Dual inoculation contexts were set up with a 10-day delay between each fungal inoculation. Fungi were inoculated on roots.
(PDF)

S3 Fig Culture of *F*. *solani* (*Fso14* strain) and *T*. *harzianum* (*Ths97* strain) cultivated separately, or together in a dual growth context related to a mycoparasitic situation. Mycelial were grown in Petri dishes on PDA medium. Slides show 6 days of growth at 27°C. Letters A, B, and C on dual culture assay correspond to area sampled for molecular experiments, with (A) *Ths97* individually, (B) area of confrontation between mycelia, and (C) area of overlap of *Ths97* on *Fso14*.
(PDF)

S1 Table Features of the non-redundant representative fungal MIP proteins from *Trichoderma* and *Fusarium* species used in the phylogenetic analysis. Reference species for MIP nomenclature: *Mycosphaerella fijiensis* (Mycfi) and *Laccaria bicolor* (Lacbi). AQP, aquaporins; AQGP, aquaglyceroporins; XIP, X-intrinsic proteins.
(PDF)

S2 Table Primers used for qPCR amplification. (PDF)

S3 Table Detail of the TFBS nucleotide sites found on 1.5kb of each promoter of the four expressed MIP. (PDF)
