# Supplementary material for: MIP diversity from Trichoderma: Structural considerations and transcriptional modulation during mycoparasitic association with Fusarium solani olive trees
Source: PLoS One. 2018 Mar 15;13(3):e0193760. doi: 10.1371/journal.pone.0193760 (PMC5854309; doi:10.1371/journal.pone.0193760)
Supplement: S1 Fig — (PDF) [file pone.0193760.s001.pdf]

S1 Fig. Detail of all MIP protein sequences used in this work.

>Triasperellum_64497
MESSEEKAPPASGDRAATQARAASAVLSVPNSSTPPTATNKMALATFDGSFAPLVRPQDVRLTPWYRRKDYYMGQ
WFEPALWRSAIVELIATCCQVFVSGQIAATIQSYGTPQIGAYIGISNLVMISTFIYAVAPASGGHINPTITFASV
LTGLCPVPRGILYMIGQTAGGALAGGILLGIWGEERAKAVHGGGCWYDPSQANPGQIYLNETFASFVLLFLAFGV
GLDPRQAALFGPRLGPVLVGASLGLVSFSTSGIIPGYAGAQMNPAKCLGNGIARLDLSYQWIYWFGPAVGGIMMG
IFYNLIPPHHVELCKLKSREMSRQITSDSMAERAEAPVVGTV*
>Triasperellum_60030
MSPSLEEEQLAWSKIREYCLDAFSEFFGTMVLILFGDGVVAQVVLSGGTKGDYQSISWGWGIAVMFGVFVGGKSG
GHLNPAVTFANCLYRGHPWRKLPVYALAQLLGAMAGAAIVYGNYKSAFDAFEGGAGIRTVTGSTATAGIFCTYPA
PFMTRTGMFFSEFIASSILMFCIFALVDPNNINAAHMMPLALFFLIFGIGACFGWETGYAMNLARDFGPRLVSYM
IGYGHEVWSAGGYYFWIPMVAPFFGTAFGGFLYDVFLYTGNSPINTPMLGVTRLFRPRKNVWSNTRPSAIDTKV*
>Triasperellum_79784
MDQSSDTNEAVSSSRRGVSENQEDQDYGSLRRRSRASRRSTLRSTAPITTAGQGTQFNLAGPSDSQQLRSAHEPF
VHPGYGDLNPSYEQPNNAKPIWSLAKPLPRVVRPGMVPTKNELLENCVNAELPAENSQKLGLDIDPNELEKGRID
KSADVRKMAAQVTDARVQRENNFIKTILASDAESTTGEIPQLVKTRSSQRRPTITRPSVQSPLYTVEEGESEHAS
TASKKRLSRESQRLDEGEGEIDLGPDELIEGNRSLETLRLDQEAYPEDLHPLVQNLVEDEIHNNNHTVWSVVRTHH
REALAESLAVFIQLTVGFCADIAVTVANAGNPNTTAWAWGFATMIGIYISGGVSGAHLNPTITIMLWFYRGFPKR
KMPEYFLAQFIGAFCACFAAYGVYYVSIQHYLSTSTGTDQDIMNCFVTNQRSAYINVPTALFNEFVGTMCLTVVV
LALGDDQNAPPGAGMNSLIIGLVITCLTISFANQTGAALNPSRDFGPRLALLALGYGSELFTNPYWFYGPFAGTL
MGSFTGAFLYDFFIFTGGESPVNYPLDRTQRALRKSRMKWRRRLHLTPKEGEDKIV*
>Triasperellum_150529
MVAQDLDRLHTHDLELGTTEATQKHIPRNVVPPRRVSQRRLDFEHSRPRWLRECIAEATGVFMYVLPGIGAIATF
TLNATSPIGASAYGSLLAIGFAFALGIAFAIITCAPTSGGHFNPAITICLCIWQGFPLKKVPRYILSQIFGGFIA
ALVLMGIYHQQIGEMKELLLAAGKPLVANGAPASILCSFPNPGQSMGYVFLTEFICDCFVGFIIWAAIDPANPFI
SPALAPLMIGMGYAVMAWSFGDVTLTMNMARDLGPRIVAAIFFGKEAFTYMNYSPIAILASIPATLVSSAFYEFV
FRDSVSVIQSGHAVHEDGDEALARHITRTTTVEGFEDKH*
>Triasperellum_142240
MANRIHTLSVRYEKALVIGVGEFCGTFMFLLLSFMGAQVALDNNPTGQRPDPATLLYIASSFGTALAVNVWVFYR
VTGGMFNPAVTLGLVLVGAVKPLRALIIVPTQIVAGIAAAAVVSGLLPGHLDVTNSLGSGTNTAQGLFIEMFLTA
QLVLTVYFLAVEKHRATFLAPVGIGVSVFIAHMAGTNFTGTGINPARSFGPAVVTGRFQAYHWIYWLGPCLGALL
SFAVYTLLKMLEYQIANPGQDAGDPETANEEALAAAEMGQEGLAMVRSRTRSGSAPSDLRFKRDSAAMRRSSAPD
GSAVSAADAV*
>Triasperellum_170459
MRQPSISRDSMRNELVVVFGEFCGTFMFLLMSYIGTQAAIDNNSPGNPDAPLFPFSLMYIAASFGTALAVNVWVF
YRVTGGMFNPAVTLGLVLVGAVKPLRGLLILPTQIVAGIAAAAVTDALLPGPLLVANKLSSGTSISRGLFIEMFL
TSQLVITVYFLAVEKHRATFLAPLGIGLSVFIAHLCGTNFTGTGINPARSFGPCVVTSFTGYQWIYWAGPFMGAL
LAFAVYSILKWMEYHNANPGQDDDS

>Triatroviride_319992
MDLAAFDGSFAPLVRPQAVRLTPWYRRRDYFVGQWFEPALWRSAVVELIATCCQVFVSGQIVATISTYGTPQLGA
YIGISNLVLIATFIYAVAPASGGHMNPTITFAAVLTGLCSVPRGLLYLVGQTAGGALGGGILLGIWGKERAIAVR
GGGCWYDPSQANPGQIYLNETFASFVLLFLAFGVGLDPRQAALFGPRMGPALVGASLGLVSFSTSGIIPGYAGAQ
MNPAKCFGNGIARMDLSYQWIYWFGPAVAGIMMGILYNLIPPHHAELCKRKSREMSREMTSDSMAERAEASVIAS
A*
>Triatroviride_283564
MCHEAIGGSLWEDRLPHAGIDETISSSRQNVRGSQEDTERGSLRRRSRTSRRSTFRSVAPLTTAGQGTQFNLAGP
SESQQLRSAHEPFVHPGYGDLNPSYEQPNNTKPIWSLAKPLPRVVRPGMVPTKNELLESCINAELPAENSQKLGL
DVDPNELEKGRIDKSADVRKMAAQVTDARVQRENNFIKTILASDAESASGDVAQLVQTRSSQRRATITRPPVQSP
LDTVEESVSDHLSTASKKRRSRESQRLDEAEGEIDLGPDELIEGNRSLETLRIDQEAYPEDLHPLVQNLVEDEIH
NNHTVWSVVRTHHREALAESLAVFIQLTVGFCADIAVTVANAGNPNTTAWAWGFASMMGIYISGGVSGAHLNPTI
TVMLWFFRGFPKRKMPEYFLAQFIGAFCACFAAYGVYYVSIQHYVSSGTGTDQEIMNCFVTNQRNPYINVPTALF
TEFVGTMCLTVVVLALGDDQNAPPGAGMNSLIIGLVIVCLTISFANQTGAALNPSRDFGPRLALLALGYGSELFT
NPYWFYGPFAGTLMGSFTGAFLYDFFIFTGGESPVNYPLDRTQRALRKSGMKWRRRLHLTPKEEEDKVV*
>Triatroviride_39327
MSPSLEEEQLAWSRIREYCMDAFSEFFGTMVLILFGDGVVAQVVLSGSTKGDYQSISWGWGIAVMFGVFVGGKSG
GHLNPAVTFANCLYRGHPWRKLPVYALAQLLGAMVGAAIVYGNYKSAFDAFEGGAGIRTVTGPTASAGVFCTYPA
PFMTRTGMFFSEFIASSILMFCIFALVDPNNVNAGHMMPLALFFLIFGIGACFGWETGYAINLARDFGPRLVSYM
IGYGHEVWSAGGYYFWIPMVAPFCGTAFGGFLYDVFLYTGNSPINTPMLGLTRLFRPRKNVWSNTRPSAIDTKV*
>Triatroviride_90169

MTTRELNRLHTHDLELGTTEATQKHVPRNVVPPRRISQRRLDFEHRRPRWLRECIAEATGVFMYVLPGIGAIATF
TLNAASPIGASAFGSLFAIGFAFALGIAFAIITCAPTSGGHFSPAVTICLCIWQGFPLKKVPYYIFSQLVGGFLA
ALVLMGIYHQQINEMKELLLAAGKPLVANGAPASILCSFPNPDQSMGYVFFTEFICDCFVGFIIWAAIDPANPFI
SPAVAPLMIGLAYAAMAWGFGDVTLTMNMARDLGPRIVAAIFFGKEAFSYKNYSPIAILVSIPATMVSSAFYEFV
FRDSVSVIQSGHAVHEDGDEALVRHITRTTTVEGIDERRGDYKS*

>Triatroviride_6990

MRRPSASRDTVRNELVVVFGEFYGTFMFLLMSYIGTQAAIDNNSPGNPEAPLFPFSLMYIAASFGTALAVNVWVF
YRVTGGLFNPAVTLGLVLVRAITPLRGLLVFPTQIVAGIAAAAVTDALLPGPLLVANKLSSGTSISRGLFIEMFL
TAQLVITVYFLAVEKHRATFLAPLGIGLAVFIAHICGTNFTGTGINPARSFGPCVVTSFTGYQWIYWAGPFMGAL
LAFAVYSILKWLEYHNANPGQDDDSNVKRNPAGFAITSNDRQYSSSTTGSKPHTPTGHNPRDSGIAQTNSAPQGF
QAV*

>Triatroviride_31598

MDSLRIFKQRSVSGDNNRNRDRFHPSTIQKHLIASAGEFVGTFFFLWFAYAGSMQYIKQATLSPPSGGISDTTVF
FIAHVYSFSLLVNVWAFYRISGGLFNPAVTLGMCLAGTLPWVRAAFLVPAQIIASMCAGGLARCMFPGDLAVANS
VLSSDTSIVRGLFIEMFFTAFLVFVVLMLSAERSKDTFIAPIGIGLALFVAMLAGTSYTGASLNPVRSFGCAVAT
PSFPGYEWIYWLGPFMGALVAAGFYRFVKLSHYEEANPNREETDHPTGDQP*

>Triatroviride_43816

MPANRIHTLSKRYEKALVIGVGEFCGTFMFLLLSFMGAQVALENNPAADGHKLEPATLLYIASSFGTALAVNVWV
FYRVTGGMFNPAVTLGLVLVGAVKPLRALIIILPTQIIAGIAAAATVSALLPGHLDVTNSLGGGTSTAQGLFIEMF
LTAQLVLTVYFLAVEKHRATFLAPVGIGVSVFIAHLAGTNFTGTGINPVRSLGPAVVTGRFEGYHWIYWLGPCLG
ALLSFAVYSLLKALEYQIANPGQDAGDPETANEEALAVEEMGTEGLAIVRSRTRSGSAASEARYKRDVAMRRGSA
VDGSAVSAADAV*

>Tricitrinoviride_1108082

MDLATFDGSFAPAVRPRGVRLTPWYRSRDYFVGQWLDVSVWKSAVVEMIATSCLVFISGQITATIEGYGTPQVGG
YIGISNIILLSTFIYATAPASGGHLNPLITFSAILTGLCSVPRGILYLSGQTLGGALAGGLLLGVWGRERATSLQ
GGGCWYDPSQASPGQVYLNEAFSSFVLLFLSFGVGLDPRQAALFGPRMGPLLVGASLGLVTFSSSGIIPGYAGAQ
MNPSRCLAYGIARRNMTYQWVWWFGPAVGALMMAVFYNLIPPHHTELTKQKSKETEPDTMAGHTDIPVV*

>Tricitrinoviride_161710

MREPFSEFFGVLILVLFGDGSIAQVVLGQGAKGDWQNINWGWALGLMLGVYSAGVSGAHLNPAVTLANCVFRKFP
WKKLPVYVLAQLLGAMVASLIVYGNYKSAIDVYEGGHGVRTVGLSTSTAGIFATYPEPFLTKTGQFFDEFTGSAI
LLFCLYALQDDGNIGAGNFLPLGLFFVFYGIGACFGSNTGYAVNPARDLGPRIMTWAVGYGHQVWTAGDYYFWVP
VIAPFLGCLFGGFLYDTFIYTGDSPINAPYMGFTRVLGKRVKAGKPVMV*

>Tricitrinoviride_1115377

MGNPTFNDAERAIFLSHSLEEEEEQLAWSKIRGYCQDFFSEFLGTMILILFGDGVVAQVVLSGGTKGDYQSISWG
WGIAVMLGVYVGGKSGGHLNPAVTLANCLYRGHPWRKLPVYALAQLLGAMAGAAIVYGNYKSAFDAFEGGPGIRT
VTGPTATAGVFCTYPAPFMTRTGMFFSEFVASSILMFCIFALADPNNIGAGNLMPLCLFFLIFGIGACFGWETGY
AINLARDFGPRLVSFMIGYGHEVWSAGGYYFWIPMVAPFLGCAFGGFLYDVFIFTGQSPINTPMLGLQRLLRPRK
SVWSNTHQSAIETKV*

>Tricitrinoviride_1172378

MSSPDLNRLHTHDLELGTTEVVQKHVTRNVVPPRRVSQRRLDFEHARPRWLRECIAEATGVFMYVLPGIGAIASF
TFNATNPVGSTAFGSLFSIGFAFALGIAFAIITCAPTSGGHFSPAVTIALCIWQGFPLKKVPYYIFSQLLGGFLA
ALVLMGIYHQQVVEMKEALLAAGEPLVANGAPASILCSFPNPDQTMGYVFMTEFFCDCFVSLIIWACLDPANPFV
SPAIALLVIGLAYGAMAWGFGANTLSMNTARDLGPRVVAAIFFGSEAFSYKNYAPIGILVSIPAMLVATAFYELV
MKDSLNIIETGHAVHADGEEALVRHITRTTTVDEKH*

>Tricitrinoviride_61920

MAFSGISKRLSASNGDGRSREHFHPSPMQQHLIAATGEFVGTFFFLWFGYAGSMQYVKLETLSPSSGGMPDTTVF
FLAHVYSFSLLVNVWAFYRISGGLFNPALTLGMCLAGTLPWMRAAFLVPAQIIASMCAGGLASAMFPGDIAAANS
LLSSDTSIARGLFIEMFFTAFLVFVVLMLAVERSKDTFIAPIGIGLALFVAMLAGTSFTGASLNPVRSFGCAVAT
PHFPGYEWIYWLGPFMGAVVAAGFYKFVKWSHYEEANPQRDATDYDHGQESQPAQHSEV*

>Tricitrinoviride_1143547

MPNRLSGSFKTALVVAVGEFCGTFMFLLLSFMGAQTALNTSGGLLDASTLLYIASSFGTALAVNVWVFYRVTGGM
FNPAVTLGLVLVGAVKPIRALYIFPSQLIAAIAAAAVTDGLLPGALGVTNGLGSGTSVVQGLFIEMFLTAQLVLT
VYFLAVEKHRATFLAPVGIGVSVFIAHMAGTNFTGTGINPVRSLGPAIVTGHYHGYHWIYWLGPCLGALLSFGVY
SLLKVLEYQIANPGQDAGDPEEANEEALAAAEMGTEGLAMFRSRSRSGSTAPGGVGLRKDLRRGSAADGGRPVSA
AEAV*

>Tricitrinoviride_1171100

MARILSSRDKIRNELIVVFGEFCGTFMFLLMAFIGTQAAIENNSPGNPDAPLFPFSLMYIAASFGTAVAVNVWVF
YRITGGMFNPAVTLGLVLVGAVSPLRGILIFPTQLVAGIAAAAVTDALLPGPLLVANKLSSGTSISRGLFIEMFL
TAQLVITVYFLAVEKHRATFLAPLGIGLAVFIAHICGTNYTGTSINPARSFGPAVVTDFTGYQWIYWLGPFLGSF
LAFVVYTILKWMEYHTANPGQDDDENKVKKSPAGLALPTNDRQYGNGSENAKNHSPSGPRDSGIAHTTSGPRGFA
AV*

>Trigamsii_JPDN01000001.1
MDLAAFDGSFAPLVRPQAVRLTPWYRRRDYFVGQWFEPALWRSAVVEMIATCCQVFVSGQIVATISTYGTPQLGA
YIGISNLVLIATFIYAVAPASGGHMNPMITFAAVLTGLCSVPRGILYMVGQTAGGALGGGILLGIWGKERAIAVR
GGGCWYDPSQANPGQIYLNETFASFVLLFLAFGVGLDPRQAALFGPRMGPALVGASLGLVTFSTSGIIPGYAGAQ
MNPAKCFGNGIARLDLSYQWIYWFGPAVGGIMMGILYNLIPPHHAELSKRKSREMSREITSDSMAERAEASVIAS
S*
>Trigamsii_ANCB02000213.1
MSPSLEEEQLAWSRIREYCMDAFSEFFGTMVLILFGDGVVAQVVLSGNTKGDYQSISWGWGIAVMFGVFVGGKSG
GHLNPAVTLANCLYRGHPWRKLPVYALAQLLGAMAGAAIVYGNYKSAFDAFEGGAGIRTVTGPTATAGVFCTYPA
AFMTRTGMFFSEFIASSILMFCIFALADPNNVNAGHMMPLALFFLIFGIGACFGWETGYAINLARDFGPRLVSYM
IGYGHEVWSAGGYYFWIPMVAPFLGTAFGGFLYDVFLYTGDSPINTPMLGLTRLFRPRKNVWSNTRPSAIDTKV*
>Trigamsii_JPDN01000042.1
MDQSPSPHRSSSNDGAGDTTSPTRPHAGSQEDQERGSLRRRSRTSRRSTFRSVAPLTTAGQGTQFNLAGPSESQQ
LRSAHEPFVHPGYGDLNPSYEQPNNAKPIWSLAKPLPRVVRPGMVPTKNELLESCINAELPAENSQKLGLDVDPN
ELEKGRIDKSSDVRKMAAQVTDARVQRENNFIKTILASDAESASGDVAQLVKTRSSQRRATITRPPVQSPLDTVE
EGVSELGSTTSKKRLSRESQRPDEAEGEVDLGPDELIEGSRSLETLRIDQEAYPEDLHPLVQNLVEDEIHNNHTV
WSVIRTHHREALAESLAVFIQLTVGFCADLAVTVANAGNPNTTAWAWGFASMMGIYISGGVSGAHLNPTITIMLW
FFRGFPKRKMPEYFLAQFIGAFCACFAAYGVYYVSIQHYLSSDTGTDQEIMNCFVTNQRYPYINVPTALFTEFVG
TMCLTVVVLALGDDQNAPPGAGMNSLIIGLVIVCLTISFANQTGAALNPSRDFGPRLALLALGYGSELFTNPYWF
YGPFAGTLMGSFTGAFLYDFFIFTGGESPVNYPLDRTQRAIRKSGIKWRRRLHLTPKEEEDKVV*
>Trigamsii_JPDN01000071.1
MTTRELNRLHTHDLELGTTEATQKHIPRNVVPPRRISQRRLDFEHRRPRWLRECIAEATGVFMYVLPGIGAIATF
TLNGASPLGVSAFGSLFAIGFAFALGIAFAIITCAPTSGGHFSPAVTICLCIWHGFPLKKVPYYIFSQLLGGFIA
ALVLMGIYHQQINEMKELLLAAGKPLVANGAPASILCSFPNPDQSMGYVFLTEFICDCFVGFIIWAAIDPANPFI
SPAIAPLMIGLAYAAMAWGFGDATLTMNTARDLGPRIVAAIFFGKEAFTYMNYSPIAILASIPATLVSSAFYEFV
FRDSVTIIQSGHAVHEDGDEALVRHLTRTTTVEGIEERRTEYKS*
>Trigamsii_JPDN01000006.1
MSGHNSLAKAAAVTLRSDDNRPNRDGFHPSTIQKHLIASVGEFVGTFFFLWFAYAGSMQYVKQATLSPPTGGLSD
TTVFFIAHVYGFSLLVNVWAFYRISGGLFNPAVTLGMCLAGTMPWARAAFLVPAQIIASMCAGGLARCMFPGDLA
VANSVLSRDTSIVRGLFIEMFFTAFLVFVVLMLSAERSKDTFIAPIGIGLALFVAMLAGTSYTGASLNPVRSFGC
AVATPSFPGYEWIYWLGPFMGALVAAGFYRFVKWSHYEEANPNREETDYPNGDQPQTAQHSSV*
>Trigamsii_JPDN01000108.1
MAANRAHALSRRYEKALVIFVGEFCGTFMFLLLSFMGAQVALDNNPATDGQKLDPATLLYIASSFGTALAVNVWV
FYRVTGGMFNPAVTLGLVLVGAVKPFRALIIVPTQIVAGIAAAAAVSALLPGHLDVTNSLGSGTNTAQGLFIEMF
LTAQLVLTVYFLAVEKHRATFLAPVGIGVSVFIAHLAGTNFTGTGINPARSFGPAVVTGRFRGYHWIYWLGPCLG
ALLSFAVYSLLKGLEYQIANPGQDAGDPETANEEALAVEEMGTEGLAIVRSRTRSGSAASETRLKRDAAMRRGSA
VDGGSVSAADAV*
>Trigamsii_JPDN01000082.1
MHRPAISRERFRNEFVVVFGEFCGTFMFLLTAYIGTQAAIDNNSPGNPDAPLFPFSLMYIAASFGTALAVNVWVF
YRVTGGLFNPAVTLGLVLVRAITPLRGLLIFPTQIVAGIAAAAVTDALLPGPLLVANKLSSGTSISRGLFIEMFL
TAQLVITVYFLAVEKHRATFLAPLGIGLAVFIAHICGTNFTGTGINPARSFGPCVVTSFTGYQWIYWAGPFMGAL
LAFAVYSILKWMEYHNANPGQDDDSNVKKSAAGFRLTSNDRQFSSSTNGSKPQTPGEHNPRDSGIGHSTSIPQGF
QAV*

>Triguizhouense_LVVK01000017.1
MELLSPPLEEEQLAWSKIRSYCQDFFSEFLGTMTLILFGDGVVAQVVLSGGTKGDYQSISWGWGIAVMLGVYVGG
KSGGHLNPAVTFANCLYRGHPWRKLPVYALAQLLGAMTGAAIVYANYKSAFDAFEGGAGIRTVTGPTATAGVFCT
YPAAFMTRTGMFFSEFIASSILMFCIFALADPNNIGAGNLMPLCLFFLIFGIGACFGWETGYAINLARDFGPRLV
SFMIGYGHEVWSAGGYYFWIPMVAPFCGCAFGGFLYDVFIFTGNSPINTPMLGLQRLMRPRKSVWSNTHPAAIEN
KV*
>Triguizhouense_LVVK01000019.1b
MREPFSEFFGVMILVLFGDGSVAQVVLGKGAKGDWNNINWGWALGVMLGVYCGGVSGAHLNPAVTLANCIFRKFP
WKKLPVYALAQLLGAMVASLIVYGNYKSAIDVFEGGQGIRTVGLDTSTAGIFCTYPAPFLTKTGQFFDEFIGSSI
LMFCLYALLDDGNVGAGNLTPLGLFFVIYGIGACFGSNTGYAINPARDLGPRIMSHAVGYGHQVWTAGDYYFWVP
VIAPFLGCTFGGFLYDTFIYTGDSPINAPYMGFTRSMGVRAKASRPTMV*
>Triguizhouense_LVVK01000013.1
MTSSRRVLNETREDQDRSSLRRRSRASRRSTFRSVAPITTAGQGTQFNLAGPTDNPQLRTAHEPFVHPGYSDLNP
SYEQPNNAKPIWSLAKPLPRVVRPGMVPTKNELLENCVNAELPAENSQNLGLDVDPNEIEKGRIEKSADLRKMGA
QVTDARQQRENNFIKTILAADAEAQSDGVAQLVKMRSSQRRATITRPSIQSPLYTVQEGLSEHTSERRKDSHESQ
RSGQVEGEFDPGPDELIEGNRSLETLRLDEDAYPEDLHPLVQELVEEEIHNNHTIWSVIRTHHREALAESLAVFV
QLTIGFCADLSVTVAKAGNPNTTDWAWGFATMIGIYISGGVSGAHLNPTITIMLWFFRGFPKRKMPEYFLAQFLG
AFCACFAAYGVYYVSIKHYLLTGVDDDIINCFVTSQRSSYINAPTALFNEFIGTMCLTIVVLALGDDQNAPPGAG

MNSLIIGLIITCLSMSFANQTGAALNPSRDFGPRLALLALGYTSELFTNPYWFYGPWAGTLLGSFMGAFLYDFFI
FTGGESPINYPLERTQRAFHKSGMKWRRRLHLTPKQEEERIV*
>Triguizhouense_LVVK01000015.1
MSSPTEQLNRLHTHDVELGTTDAVQKHISRNVVPPRRVSQRRLDFEHRRPRWLRECIAEATGVFMYVLPGIGAIT
SFTINATNPIGSTAFGSLFSIGFAFALGIAFAIITCAPTSGGHFSPAVTIALWFWQGFPLKKVPYYIFSQLLGGF
IAALVLMGIYHQQLDEMKAVLLAAGEPLVANGAPASVLCSFPNPGQSMGYVFMTEFFCDCFVGLIIWACLDPANP
FVSPSLAPLVIGLAYGAMAWGFGANTLTMNMARDFGPRVVAAIFYGREAFSYMNYAAIGIFTSIPATLVSSAFYE
FVMRDSLSVIGTGHAVHADGDEALVRHITRTTTVDGIEERRGEYKS*
>Triguizhouense LVVK01000015.1
MASSGIFKQHSGSGDNGRSRDGFHPSTIQKHLIASAGEFVGTFFFLWFAYAGSMQYVKQATLSPLSGGISDTTVF
FIAHVYSFSLLVNVWAFYRISGGLFNPAVTLGMCLAGTLPWARAAFLVPAQIIASMCAGGLARCMFPGDLAVANS
VLSSDTSIVRGLFIEMFFTAFLVFVVLMLAAERSKDTFIAPIGIGLALFVAMLAGTSYTGASLNPVRSFGCAVAT
PSFPGYEWIYWLGPFMGAVVAAGFYRFIKWSRYEEVNPNREETDHPNGDQPQPAQHSSV*
>Triguizhouense_LVVK01000004.1
MHRQYAPPREPIRNEIVVVFGEFCGTFMFLLMSFIGTQAALDNNDPTNPNAPLFPFSLLYVASSFGAALAVNVWV
FYRVTGGMFNPAVSVELLLVGAVKPIRGLLIFPAQIVAGIAAAAVTDALLPGPLLVANKLASGTSISRGLFIEMF
LTAQLVITVYFLAVEKHRATFLAPLGIGLAVFIAHICGTNFTGTGINPARSFGPAVVTDFTGYQWIYWVGPLLGS
LLAFAVYTILKWLEYHNANPGQDDDSFVKKTPAGFNIPTGDRQYSNSTNGAKEHTPSEPRDSGVVQTNSGPQGFQ
AV*
>Triguizhouense_LVVK01000020.1
MASLQEKTHPAGDESPPAARISTASDQRPATSKMDLAAFDGSFAPGVRPGAVRLTPWYRRRDYFVGQWVDISVWK
SAVVEFVATSCLVFLSGQITATLESYGTPQVGGYIGISNIILIATFIYATAPASGGHLNPMITFSAILTGLCSVP
RGILYMCAQTLGGALAGGILLGVWGPKRATSLQGGGCWYDPSQANPGQIYLNEVFASFVLLFLSFGVGLDPRQAA
LFGPRMGPLLVGASLGLVSFATSGIIPGYAGAQMNPSRCLAFGIARRDMTCQWVYWFGPAVGCLMMGVFYNLIPP
HHVELSKQKSKESRMNSMAGNTEVPEV*
>Triguizhouense_LVVK01000018.1
MAKPLRRFTERFKTALVIGLGEFCGTFMFLLLSFMGAQAALDNGPDGGKLDASTLLYIASSFGTALAVNVWVFYR
VTGGMFNPAVTLGLVLVGAVKPLRALIIILPMQIAAGIAAAATVSGLLPGPLSVTNSLGSGTSIVQGLFIEMFLTA
QLVLTVYFLAVEKHRATFLAPVGIGVSVFIAHMAGTNFTGTGINPVRSLGPAVVTGSFRGYHWIYWLGPCLGALL
SFTVYSLLKGLEYQIANPGQDAGDPETANEEAIAAAEMGQEGLAMVRSRSRSMSVPNDMMFKKEREAAMRRASVP
EESPMSAADVV*

>Trihamatum_ANCB02000213.1
MDLAAFDGSFAPLVRPQAVRLTPWYRRRDYFVGQWFEPALWRSAIVELIATCCQVFVGGQIAATIQSYGTPQVGA
YIGISNLVMISIFIYAVAPASGGHMNPLITFAAVLTGLCSVPRGMLYIIGQTAGGALAGGILLGIWGEERAIAVR
GGGCWYDPSQANPGQIYLNETFASFVLLFLAFGVGLDPRQAALYGPRMGPVLVGASLGLVSFSTSGIIPGYAGAQ
MNPAKCFGNGIARLDLSYQWIYWFGPAVAGIMMGIFYNLIPPHHAELCKKKSREMSREITSDSMAERAEAPVVGT
V
>Trihamatum_ANCB02000044.1
MSPSLEEEQEQLAWSRIREYCMDFFSEFLGTMVLILFGDGVVAQVVLSGGTKGDYQSISWGWGIAVMFGVFVGGK
SGGHLNPAVTFANCLYRGHPWRKLPVYALAQLLGAMVGAAIVYGNYKSAFDAFEGGAGIRTVTGSTATAGVFCTY
PAPFMTRTGMFFSEFIASSILMFCIFALVDPNNINAGHMMPLALFFLIFGIGACFGWETGYAINLARDFGPRLVS
YMIGYGHEVWSAGGYYFWIPMVAPFCGCAFGGFLYDVFLYTGNSPINTPMLGLTRLFRPRKNVWSNTRPSAIDTK
V*
>Trihamatum_ANCB02000079.1
ELGTTEATQKHIPRNVVPPRRISQRRLDFEHSRPRWLRECIAEATGVFMYVLPGIGAIATFTLNATSPIGASAYG
SLFAIGFAFALGIAFAIITCAPTSGGHFSPAVTICLCIWQGFPLKKVPYYIFSQLVGGFLAALVLMGIYHQQISE
MKELLVAAGKPLVANGAPASILCSFPNPGQSMGYVFFTEFICDCFVGFIIWAAIDPANPFVSPALAPLMIGLAYA
CMAWGFGDVTLTMNMARDLGPRIVAAIFFGKEAFTYMNYSPIAILVSIPATMVSSAFYEFVFRDSVSVIQSGHAV
HEDGDEALVRHITRTTTVEGIEERAGGDYKS*
>Trihamatum_ANCB02000059.1
MDQSSDPAHSSSNDGAGNPTSPIRQATDEAVSSSRRDISENQDDQDYGSLRRRSRASRRSTIRSTAPITTAGQGT
QFNLAGPSDSQQLRSAHEPFVHPGYGDLNPSYEQANNAKPIWSLAKPLPRVVRPGMVPTKNELLENCINAELPAE
NSQKLGLDVDPNELEKGRIDKSSDVRKMAAQVTDARVQRENNFIKTILASDAESASGDLASVVKTRSSQRRLTLQ
QPSVHSHLSTVQEGASETGSTVSKKKRPSRESQRLDEAEGEIDLGPDELIEGNRSLETLRLDQEAYPEDLHPLIQ
NLVEDEIHNNHTVWSVVRTHHREALAESLAVFIQLTIGFCADIAVTVANAGNPNTTAWAWGFATMIGIYISGGVS
GAHLNPTITMMLWFYRGFPKRKMPEYFLAQFLGAFCACFAAYGVYYVSIQHYLSSDTGTDQEIMNCFVTNQRSAF
ISVPTALFNEFVGTMCLTVVVLALGDDQNAPPGAGMNSLIIGLVITCLTISFANQTGAALNPSRDFGPRLALLAL
GYGSELFTNPYWFYGPFAGTLMGAFTGAFLYDFFIFTGGESPVNYPLDRTQRALRKSRMKWRRRLHLTPREGEDK
VV*
>Trihamatum_ANCB02000398.1
MARLSERYEKALVICLGEFCGTFMFLLLSFMGAQVALDNNPTGQKLDPATLLYIASSFGTALAVNVWVFYRVTGG
MFNPAVHEYPMSIKVTLGLALVGAVTPLRALIIVPTQIVAGIAAAAVVSGLLPGHLDVTNSLGSSTNTAQGLFIE

MFLTAQLVLAVYFLAVEKHRATFLAPVGIGVSVFIAHMAGTNFTGTGINPARSFGPAVVTGKFPRYHWIYWLGPC
LGALLSFVVYTLLKLLEYPIANPGQDAGDPETANEEALAAAQMGQQGLALVRSRTKSGSAPSDLRFKRDSTAMRG
GSVPEGSAVSAADAV*
>Trihamatum_ANCB02000229.1
MRRPPTPREKIRNELVVVFGEFCGTFMFLLMSYIGTQAAIDNNSPGNPDAPLFPFSLMYIAASFGTALAVNVWVF
YRVTGGMFNPAVTVGLVLVGAVKPLRGLCIFPTQIVAGIAAAAVTDALLPGPLLVANKLSSGTSISRGLFIEMFL
TSQLVITVYFLAVEKHRATFLAPLGIGLSVFIAHLCGTNFTGTGINPARSFGPAVVTDFTGYQWIYWVGPFLGAL
LAFVVYTILKWMEYHTANPGQDDDSHVKRNPAGLAISSNDRQYSNSTNGSKPQTPGEHNPRDSGIAQTNSTPQGF
QAV*

>Triharzianum_488926
MDLAAFDGSFAPGVRPGAVRLTPWYRRRDYFVGQWVDISVWKSAVVEFVATSCLVFLSGQITATLESYQTPQVGG
YIGISNIILIATFIYATAPASGGHLNPMITFSAILTGLCSVPRGILYMCAQTLGGALAGGILLGVWGPKRATSLQ
GGGCWYDPSQANPGQIYLNEVFASFVLLFLSFGVGLDPRQAALFGPRMGPLLVGASLGLVSFATSGIIPGYAGAQ
MNPSRCLAFGIARRDMTYQWVWWFGPAVGCLMMGIFYNLIPPHHVELSKQKSKESRMNSMAGNTEVPEV*
>Triharzianum_90014
MELLSPPLEEEQLAWSKIRSYCQDFFSEFLGTMTLILFGDGVVAQVVLSGGTKGDYQSISWGWGIAVMLGVYVGG
KSGGHLNPAVTFANCLYRGHPWRKLPVYALAQLLGAMTGAAIVYANYKSAFDAFEGGAGIRTVTGPTATAGVFCT
YPAAFMTRTGMFFSEFIASSILMFCIFALADPNNIGAGNLMPLCLFFLIFGIGACFGWETGYAINLARDFGPRLV
SFMIGYGHEVWSAGGYYFWIPMVAPFCGCAFGGFLYDVFIFTGNSPINTPMLGLQRLMRPRKSVWSNTHPAAIEN
KV*
>Triharzianum_490850
MREPFSEFFGVLILVLFGDGSVAQVVLGKGAKGDWNNINWGWALGVMLGVYCGGVSGAHLNPAVTLANCIFRKFP
WKKLPVYALAQLLGAMAASLIVYGNYKSAIDVFEGGQGIRTVGLDTSTAGIFCTYPAPFLTKTGQFFDECIGSSI
LMFCLYALLDDGNIGAGNLTPLGLFFVIYGIGACFGSNTGYAINPARDLGPRIMSHAVGYGHQVWTAGDYYFWVP
VIAPFLGCTFGGFLYDTFIYTGDSPINAPYMGFTRFMGVHAKARRTAMV*
>Triharzianum_82211
MTSSRRVLNETREDQDRSSLRRRSRASRRSTFRSVAPITTAGQGTQFNLAGPTDTPQLRTAHEPFVHPGYSDLNP
SYEQPNNAKPIWSLAKPLPRVVRPGMVPTKNELLENCVNAELPAENSQNLGLDVDPNEIEKGRIEKSADLRKMGA
QVTDARQQRENNFIKTILAADAEAQSDGLPQLVKTRSSQRRATITRPSIQSPLYTVQEGLSEHTSERHKDSHESQ
RSGQGEGEFDPGPDELIEGNRSLETLRLDQDAYPEDLHPLVQDLVEEEIHNNHTIWSVIRTHHREALAESLAVFV
QLTIGFCADLSVTVAKAGNPNTTDWAWGFATMIGIYISGGVSGAHLNPTITIMLWFFRGFPKRKMPEYFLAQFLG
AFCACFAAYGVYYVSIKHYLLTGVDDDIINCFVTSQRSSYINAPTALFNEFIGTMCLTIVVLALGDDQNAPPGAG
MNSLIIGLIITCLSMSFANQTGAALNPSRDFGPRLALLALGYTSELFTNPYWFYGPWAGTLLGSFMGAFLYDFFI
FTGGESPINYPLERTQRAFHKSGMKWRRRLHLTPKQEEERIV*
>Triharzianum_92358
MSSPTEQLNRLHTHDLELGTTDAVQKHISRNVVPPRRVSQRRLDFEHRRPRWLRECIAEATGVFMYVLPGIGAIT
SFTINATNPIGSTAFGSLFSIGFAFALGIAFAIITCAPTSGGHFSPAVTIALWFWQGFPLKKVPYYIFSQLLGGF
IAALVLMGIYHQQLDEMKQVLLAAGEPLVANGAPASVLCSFPNPGQSMGYVFMTEFFCDCFVGLIIWACLDPANP
FVSPSLAPLVIGLAYGAMAWGFGANTLTMNMARDFGPRVVAAIFYGREAFSYMNYAAIGIFTSIPATLVSSAFYE
FVMRDSLSVIGTGHAVHADGDEALVRHITRTTTVDGIEERRGEYKS*
>Triharzianum_99286
MASSGIFKQHSGSGDNGRSRDGFHPSTIQKHLIASAGEFVGTFFFLWFAYAGSMQYVKQATLSPLSGGISDTTVF
FIAHVYSFSLLVNVWAFYRISGGLFNPAVTLGMCLAGTLPWVRAAFLVPAQIIASMCAGGLARCMFPGDLAVANS
VLSSDTSIVRGLFIEMFFTAFLVFVVLMLAAERSKDTFIAPIGIGLALFVAMLAGTSYTGASLNPVRSFGCAVAT
PSFPGYEWIYWLGPFMGAVVAAGFYRFIKWSRYEEVNPNREETDHPNGDQPQPAQHSSV*
>Triharzianum_98742
MHRQYAPPREPIRNEIVVVFGEFCGTFMFLLMSFIGTQAALDNNDPTNPNAPLFPFSLLYVASSFGAALAVNVWV
FYRVTGGMFNPAVTLGLVLVGAVKPIRGLLIFPAQIVAGIAAAAVTDALLPGPLLVANKLASGTSISRGLFIEMF
LTAQLVITVYFLAVEKHRATFLAPLGIGLAVFIAHICGTNFTGTGINPARSFGPAVVTDFTGYQWIYWVGPLLGS
LLAFAVYTILKWLEYHNANPGQDDDNFVKKTPAGFNIPTGDRQYSNSTNGAKEHTPSEPRDSGVVQTNSGPQGFQ
AV*
>Triharzianum_485859
MAKPLRKFTERFKTALVIGLGEFCGTFMFLLLSFMGAQAALDNGPDGGKLDASTLLYIASSFGTALAVNVWVFYR
VTGGMFNPAVTLGLVLVGAVKPLRALIIILPMQIAAGIAAAATVSGLLPGPLSVTNSLGSGTSIVQGLFIEMFLTA
QLVLTVYFLAVEKHRATFLAPVGIGVSVFIAHMAGTNFTGTGINPVRSLGPAVVTGSFRGYHWIYWLGPCLGALL
SFTVYSLLKGLEYQIANPGQDAGDPETANEEAIAAAEMGQEGLAMVRSRSRSMSVPNDMMFKKEREAAMRRASVP
EESPMSAADVV*

>Trikoningii_BCGH01000004.1a
MELLSPPLEEEQLAWSKIRNYCQDFFSEFLGTMTLILFGDGVVAQVVLSGGTKGDYQSISWGWGIAVMLGVYVGG
KSGGHLNPAVTLANCLYRGHPWRKLPVYALAQLLGAMAGAAIVYGNYKSAFDAFEGGAGIRTVTGPTATAGVFCT
YPAPFMTRTGMFFSEFVASSILMFCIFALADPNNIGAGNLMPLCLFFLIFGIGACFGWETGYAINLARDFGPRLV

SYMIGYGHEVWSAGGYYFWIPMVAPFCGCAFGGFLYDVFIYTGNSPINTPMLGLQRLMRPRKSVWSNTHPSAIES
KV*
>Trikoningii_BCGH01000004.1
MREPFSEFFGVLILVLFGDGSIAQVVLGQGAKGDWQNINWGWALGLMLGVYCAGASGAHLNPAVTLANCVFRKFP
WNKLPVYILAQLLGAMIASLIVYGNYKSAIDVYEGGQGIRTVGLSTSTAGIFCTYPAPFLTKAGQFFDEFTGSAI
LLFCLYALQDDGNIGAGNLLPLGLFFVFYGIGACFGSNTGYAVNPARDLGPRIMTSAVGYGHQVWTAGDYYFWVP
IIAPFLGCLFGGFLYDTFIFTGDSPINLPYMGLTRGLGNRTKTGKPIMV*
>Trikoningii_BCGH01000008.1
MSSPTEQLNRLHTHDVELGTTDVVQKHATRNVVPPRRVSQRRLDFEHARPRWLRECIAEATGVFMYVLPGIGAIA
SFTFNATNPVGSTAFGSLFSIGFAFALGIAFAIITCAPTSGGHFSPAVTIALCVWQGFPLKKVPHYIFSQLLGGF
IAALVLMGIYHQQIVEMKEALLAAGEPLVANGAPASILCSFPNPDQTMGYVFLTEFFCDCFVSLIIWACIDPANP
FVSPAIAPLVIGLGYGAMAWGFGANTLSMNMARDLGPRIVAAIFFGGEAFTYKNYAPIGILVSIPAMMVATAFYE
LVMKDSLNIIETGHAVHEDGEEALVRHITRTTTVDGLEERSGEYKS*
>Trikoningii_BCGH01000002.1
MAFPGLSKRHAAANGDGRSREQGFHPSPMQKHLIAAAGEFVGTFFFLWFAYAGSMQYTKLATLSPPSGGMADTTV
FFIAFVYSFSLLVNVWAFYRISGGLFNPAVTLGMCLAGTLPWARAAFLVPAQIIASMCAGGLASAMFPGNIAAAN
SLLSSDTSIVRGLFIEMFFTAFLVFVVLMLAAERSKDTFIAPIGIGLALFVAMLAGTSYTGASLNPVRSFGCAVA
TPHFPGYEWIYWLGPFMGAALAAGFYKFVKWSHYEEANPQRDATDYDHDPRSQPAQHSAV*
>Trikoningii_BCGH01000009.1
MHRQYAPPREPIRNELIVVFGEFCGTFMFLLMAFIGTQAAIENNSPGNPDAPLFPFSLMYIAASFGTAVAVNVWV
FYRITGGMFNPAVSVELLLVGAVSPLRGLLIFPTQLVAAIAAAAVTDALLPGPLLVANKLSPGTSISRGLFIEMF
LTAQLVITVYFLAVEKHRATFLAPLGIGLAVFIAHICGTNYTGTSINPARSFGPAVVTDFTGYQWIYWLGPFLGS
FLAFVVYTILKWMEYHTANPGQTVCLLSPRILAAKLQKVSALRKYDSITGVICIIL*
>Trikoningii_BCGH01000003.1A
MESLQEKEPQPAGHDTTAARVQTTVDLPPSSSDTAASGRRSATSKMDLATFEGSFAPAVRPRGVRLTPWYRSRDY
FIGQWLDVSVWKSAVVEMVATSCLVFVSGQITATIEGYGTPQVGGYIGISNIILLSTFIYATAPASGGHLNPMIT
FSAILTGLCSVPRGMVLYLCGQTLGGALAGGLLLGVWGRERATSLQGGGCWYDPSQASPGQIYLNEVFSSFVLLF
LSFGVGLDPRQAALFGPRMGPLLVGASLGLVTFSSSGIIPGYAGAQMNPSRCLAFGIARRNMSDQWVWWFGPAVG
GLIEAIVYNLIPPHHMELVKQKSKETEPDTMVGHTDIPTV*
>Trikoningii_BCGH01000003.1B
MPNRLSGSFKTALVVAVGEFCGTFMFLLLSFMGAQTALNSSGGTLDAPTLLYIASSFGTALAVNVWVFYRVTGGM
FNPAVTLGLVLVGAVKPLRALYIFPSQLIAAIAAAAVTDGLLPGPLGVTNGLGSGTSVVQGLFIEMFLTAQLVLT
VYFLAVEKHRATFLAPVGIGVSVFIAHMAGTNFTGTGINPVRSLGPAIVTGRFNGYHWIYWLGPVLGALLSFGVY
SLLKVLEYQVANPGQDAGDPEEANEEAIAAAEMGTEGLAMFRSRSRSGSAPGGVGLRKDSALRRGSAPEGGRPVS
AAEAV*

>Trilongibrachiatum_1343482
MDLATFEGSFAPAVRPRGVRLTPWYRSRDYFIGQWLDVSVWKSAVVEMVATSCLVFVSGQITATIEGYGTPQVGG
YIGISNIILLSTFIYATAPASGGHLNPMITFSAILTGLCSVPRGVLYLCGQTLGGALAGGLLLGVWGRERATSLQ
GGGCWYDPSQASPGQIYLNEVFSSFVLLFLSFGVGLDPRQAALFGPRMGPLLVGASLGLVTFSSSGIIPGYAGAQ
MNPSRCLAFGIARRNMSYQWVWWFGPAVGGLIEAIVYNLIPPHHMELVKQKSKETEPDTMVGHTDIPTV*
>Trilongibrachiatum_1439288
MREPFSEFFGVLILVLFGDGSIAQVVLGQGAKGDWQNINWGWALGLMLGVYCAGASGAHLNPAVTLANCVFRKFP
WNKLPVYILAQLLGAMIASLIVYGNYKSAIDVYEGGQGIRTVGLSTSTAGIFCTYPAPFLTKAGQFFDEFTGSAI
LLFCLYALQDDGNIGAGNLLPLGLFFVFYGIGACFGSNTGYAVNPARDLGPRIMTSAVGYGHQVWTAGDYYFWVP
IIAPFLGCLFGGFLYDTFIFTGDSPINLPYMGLTRGLGNRTKTGKPIMV*
>Trilongibrachiatum_1339918
MGNPSLHDAEGAIFLATSLEEEELLAWSKIRNYCQDFFSEFLGTMTLILFGDGVVAQVVLSGGTKGDYQSISWGW
GIAVMLGVYVGGKSGGHLNPAVTLANCLYRGHPWRKLPVYALAQLLGAMAGAAIVYGNYKSAFDAFEGGAGIRTV
TGPTATAGVFCTYPAPFMTRTGMFFSEFVASSILMFCIFALADPNNIGAGNLMPLCLFFLIFGIGACFGWETGYA
INLARDFGPRLVSYMIGYGHEVWSAGGYYFWIPMVAPFLGCAFGGFLYDVFIFTGPSPINTPMLGLQRLMRPRRS
VWSNTHKSAIETKV*
>Trilongibrachiatum_1362869
MSSPDLDRLHTHDLELGTTDVVQKHATRNVVPPRRVSQRRLDFEHARPRWLRECIAEATGVFMYVLPGIGAIASF
TFNATNPVGSTAFGSLFSIGFAFALGIAFAIITCAPTSGGHFSPAVTIALCVWQGFPLKKVPHYIFSQLLGGFIA
ALVLMGIYHQQIVEMKEALLAAGEPLVANGAPASILCSFPNPDQTMGYVFLTEFFCDCFVSLIIWACIDPANPFV
SPAIAPLVIGLGYGAMAWGFGANTLSMNMARDLGPRIVAAIFFGGEAFTYKNYAPIGILVSIPAMMVATAFYELV
MKDSLNIIETGHAVHEDGEEALVRHITRTTTVDEKS*
>Trilongibrachiatum_1340117
MPNRLSGSFKTALVVAVGEFCGTFMFLLLSFMGAQTALNSSGGTLDAPTLLYIASSFGTALAVNVWVFYRVTGGM
FNPAVTLGLVLVGAVKPLRALYIFPSQLIAAIAAAAVTDGLLPGPLGVTNGLGSGTSVVQGLFIEMFLTAQLVLT
VYFLAVEKHRATFLAPVGIGVSVFIAHMAGTNFTGTGINPVRSLGPAIVTGRFNGYHWIYWLGPVLGALLSFGVY

SLLKVLEYQVANPGQDAGDPEEANEEAIAAAEMGTEGLAMFRSRSRSGSAPGGVGLRKDSALRRGSAPEGGRPVS
AAEAV*
>Trilongibrachiatum_51184
MAFPGLSKRHAAANGDGRSREQGFHPSPMQKHLIAAAGEFVGTFFFLWFAYAGSMQYTKLATLSPPSGGMADTTV
FFIAFVYSFSLLVNVWAFYRISGGLFNPALTLGMCLAGTLPWARAAFLVPAQIIASMCAGGLASAMFPGNIAAAN
SLLSSDTSIVRGLFIEMFFTAFLVFVVLMLAAERSKDTFIAPIGIGLALFVAMLAGTSYTGASLNPVRSFGCAVA
TPHFPGYEWIYWLGPFMGAALAAGFYKFVKWSHYEEANPQRDATDYDHDPRSQPAQHSAV*
>Trilongibrachiatum_22769
MPRLLSSRDTIRNELIVVFGEFCGTFMFLLMAFIGTQAAIENNSPGNPDAPLFPFSLMYIAASFGTAVAVNVWVF
YRITGGMFNPAVTLGLVLVGAVSPLRGLLIFPTQLVAAIAAAAVTDALLPGPLLVANKLSPGTSISRGLFIEMFL
TAQLVITVYFLAVEKHRATFLAPLGIGLAVFIAHICGTNYTGTSINPARSFGPAVVTDFTGYQWIYWLGPFLGSF
LAFVVYTILKWMEYHTANPGQDDDTAAKKTPAGLAVATNDRQYRSGSEGGKYRSPSGPRDSGIAHTSSGPQGFAA
V*

>Trichoderma_pleuroti_MDJU01000355.1
MELLSPPLEEEQLAWSKIRNYCQDFFSEFLGTMTLILFGDGVVAQVVLSGGTKGDYQSISWGWGIAVMLGVYVGG
KSGGHLNPAVTFGNCLYRGHPWRKLPVYVLAQLLGAMTGAAIVYGNYKSAFDAFEGGAGIRTVTGPTATAGVFCT
YPAPFMTRTGMFFSEFIASSILMFCIFALADPNNIGAGNLMPLCLFFLIFGIGACFGWETGYAINLARDFGPRLV
SFMIGYGHEVWSAGGYYFWIPMVAPFCGCAFGGFLYDVFIYTGNSPINTPMLGLQRLMRPRKSVWSNTHPSAIES
KV*
>Trichoderma_pleuroti_MDJU01000027.1
MREPFSEFFGVMILVLFGDGSVAQVVLGKGTKGDWNNINWGWALGVMLGVYCSGVSGAHLNPAVTLANCIFRKFP
WKKLPVYALAQLLGAMAASLIVYGNYKSAIDVFEGGQGIRTVGLDTSTAGIFCTYPAPLLTKTGQFFDEFIGSSI
LMFCLYALLDDGNNGAGNLTPLGLFFVIYGIGACFGSNTGYAINPARDLGPRIMSHAVGYGKQVWTAGDYYFWVP
VIAPFLGCTFGGFLYDTFIYTGDSPINAPYMGFTRFMGVRAKARRSTMV*
>Trichoderma_pleuroti_MDJU01000063.1
MTSSRRALNESREDQDRSSLRRRSRTSRRSTFRSVAPITTAGQGTQFNLAGSTDTPQLRTAHEPFVHPGYSDLNP
SYEQPNNAKPVWSLAKPLPRVVRPGMVPTKNELLENCVNAELPAENSQNLGLDVDPNEIEKGRIEKSADLRKMGA
QVADARQQRENNFIKTILAADAEAASDGLPQLVKTRSSQRRATITRPSIQSPLYTVQEGLSEHTSERHKGSHESQ
RSAQGEGEFDPGPDELIEGNRSLETLRLDQDAYPEDLHPLVQELVEDEIHNNHTIWSVIRTHHREALAESLAVFV
QLTIGFCADLSVTVAKAGNPNTTDWAWGFATMIGIYISGGVSGAHLNPTITIMLWFFRGFPKRKMPEYFLAQFLG
AFCACFAAYGVYYVSIKHYLLTGVDDDIVNCFVTSQRSSYINAPTALFNEFIGTMCLTIVVLALGDDQNAPPGAG
MNSLIIGLIITCLSMSFANQTGAALNPSRDFGPRLALLALGYTSELFTNPYWFYGPWAGTLLGSFMGAFLYDFMI
FTGGESPINYPLERTQRAFHKSGMKWRRRLRMTPKQEEERIV*
>Trichoderma_pleuroti_MDJU01000041.1
MSSPTEQLNRLHTHDVELGTTDAVQKHISRNVVPPRRVSQRRLDFEHRRPRWLRECIAEATGVFMYVLPGIGAIT
SFTINATNPIGSTAFGSLFSIGFAFALGIAFAIITCAPTSGGHFSPAVTIALWFWQGFPLKKVPYYIFSQLLGGF
IAALVLMGIYHQQLDEMKEVLLAAGRPLVANGAPASVLCSFPNPGQSMGYVFMTEFFCDCFVGLIIWACLDPANP
FVSPSLAPLVIGLAYGAMAWGFGANTLTMNMARDFGPRVVAAIFYGREAFSYMNYAAIGIFTSIPATLISSAFYE
FVMRDSLSVIGTGHAVHADGDEALVRHITRTTTVEGLEERSGEYKS*
>Trichoderma_pleuroti_MDJU01000371.1
MHRIYSPRAPREPIRNEIVVVFGEFCGTFMFLLMSFIGTQAAIDNNDPGNPDAPLFPFSLLYVASSFGAALAVNV
WVFYRVTGGMFNPAVSVELLLVGAVKPIRGLLIFPTQIVAGIAAAAVTDALLPGPLLVANKLASGTSISRGLFIE
MFLTAQLVITVYFLAVEKHRATFLAPLGIGLAVFIAHICGTNFTGTGINPARSFGPAVVTDFTGYQWIYWVGPFL
GSLLAFAVYSILKWLEYHTANPGQDDDNTVKKTPAGFSIPTGDRQYSNGGNGAKEHVPSDPRDSGVAQTSSAPQG
FQAV*
>Trichoderma_pleuroti_MDJU01000245.1
MASLQEKTTHPAGDDSPPAARVSTASDQKPATSRMDLAAFDGSFAPGVRPGAVRLTPWYRRRDYFVGQWADMSVW
KSAVVEFVATSCVVFLSGQIAATLEGYGTPQVGGYIGISNIIILIATFIYATAPASGGHLNPMITFSAILTGLCSV
PRGILYMCGQTLGGALAGGILLGVWGPKRATSLQGGGCWYDPSQANPGQIYLNEVFASFVLLFLSFGVGLDPRQA
ALFGPRMGPLLVGASLGLVSFATSGIIPGYAGAQMNPSRCLAFGIARRDMSCQWVWWFGPAVGGLMMGVFYKLIP
PHHVELSKQKSKESRLNSMAGNTEVPEV*
>Trichoderma_pleuroti_MDJU01000029.1
MANHLHRFTDRFKTALVIGLGEFCGTFMFLLLSFMGAQAALDNRSDGGKMDAATLLYIASSFGTALAVNVWVFYR
VTGGMFNPAVTLGLVLVGAVKPLRALIIVPMQIAAGIAAAGTVSGLLPGPLGVTNSLGSNTSIVQGLFIEMFLTA
QLVLTVYFLAVEKHRATFLAPVGIGVSVFIAHMAGTNFTGTGINPARSLGPAVVTGSFRGYHWIYWLGPCLGALL
SFSVYSLLKGLEYQIANPGQDAGDPETANEEAIAAAEMGQEGLAMVRSRSRSMSVPNDTLLKKDRDAAMRRASIP
EEGPVSAAEAV*

>Trireesei_128546
MDLAAFDGSFAPAVRPREVRLAPWYRSRDYFVGQWLDVSVWKSAVVEMVATSCLVFLSGQITATIEGYGTPQVGG
YIGISNIILLSTFIYATAPASGGHLNPMITFSAILTGLCSVPRGILYMSAQTLGGALAGGLLLGVWGHERATSLQ

GGGCWYDPSQASPGQVYLNEVFSSFVLLFLSFGVGLDPRQAALFGPRMGPLLVGASLGLVTFSSSGIIPGYAGAQ
MNPSRCLAFGIARRNMSYQWVWWFGPAVGGLIEALLYNLIPPHHTELVKKQGIGNDPDTMVGHTDIPTV*

>Trireesei_92240

MGNPNIHDAERAIFLTPSLEEEELLAWSKIRNYCQDFFSEFLGTMTLILFGDGVVAQVVLSGGTKGDYQSISWGW
GIAVMLGVYVGGKSGGHLNPAVTLANCLYRGHPWRKLPVYALAQLLGAMAGAAIVYGNYKSAFDAFEGGAGIRTV
TGPTATAGVFCTYPAPFMTRTGMFFSEFVASSILMFCIFALADPNNIGAGNLMPLCLFFLIFGIGACFGWETGYA
INLARDFGPRLVSYMIGYGHEVWSAGGYYFWIPMVAPFLGCAFGGFLYDVFIFTGPSPINTPMLGLQRLMRPRRS
VWSNTHQSTIETKV*

>Trireesei_141480

MREPFSEFFGVLILVLFGDGSIAQVVLGQGAKGDWQNINWGWALGLMLGVYCAGASGAHLNPAVTLANCVYRKFP
WKKLPVYVLAQLLGAMIASLIVYGNYKSAIDVYEGGQGIRTVGLSTSTAGIFCTYPAPFLTKAGQFFDEFTGSAI
LLFCLYALQDDGNIGAGNLLPLGLFFVFYGIGACFGSNTGYAVNPARDLGPRIMTSAVGYGHQVWTAGDYYFWVP
IVAPFLGCLFGGFLYDTFLFTGDSPMNLPYMGLTRVWGNRTKTGKPVMDSVISHKHS*

>Trireesei_102402

MSSPDLNRLHTHDLELGTTEVVQKHATRNVVPPRRVSQRRLDFEHARPRWLRECIAEATGVFMYVLPGIGAIASF
TFNATNPIGSTAFGSLFSIGFAFALGIAFAIITCAPTSGGHFSPAVTIALCVWQGFPLKKVPHYIFSQLLGGFIA
ALVLMGIYHQQIVEMKEALLAAGEPLVANGAPASILCSFPNPNQTMGYVFLTEFFCDCFVSLIIWACLDPANPFV
SPAIAPLVIGLAYGAMAWGFGANTLSMNTARDLGPRVVAAIFFGSEAFTYKNYAPIGILASIPAMLVATAFYELV
MKDSLNIIETGHAVHEDGDEALVRHITRTTTVDEKH*

>Trireesei_67036

FKTALVIAVGEFCGTFMFLLLSFMGAQTALNSSGGNLDAPTLLYIASSFGTALAVNVWVFYRVTGGMFNPAVTLG
LVLVGAVKPLRALYIFPSQLIAAIAAAAVTDGLLPGPLGVTNGLGSGTSVVQGLFIEMFLTAQLVLTVYFLAVEK
HRATFLAPVGIGVSVFIAHMAGTNFTGTGINPVRSLGPAIVTGNFHGYHWIYWLGPVLGALLSFGVYSLLKVLEY
QVANPGQD

>Trireesei_129514

MAFPGLSKRQHSAPNGGESRDREHFHPTPMQKHLIAAVGEFVGTFFFLWFGYAGSMQYTKLATLSPSSPGGMDDT
TVFFLAFVYSFSLLVNVWAFYRISGGLFNPALTFGMCLAGTLDWARAAFLVPAQLIASMCAGGLASAMFPGNISA
ANSLLSSDTSIAQGLFIEMFFTAFLVFVVLMLAVERSRYTFIAPVGIGLALFVVMLAGTSYTGASLNPARSFGCA
VATPHFPGYEWIYWLGPFMGAVVAAGFYKFVKWSHYEEVNPQRDATDYDHSHQPQPAGHSLV*

>Trireesei_143256

MPRLLSSRDTIRNELIVVFGEFCGTFMFLLMAFIGTQAAIENNSPGNPDAPLFPFSLMYIAASFGTAVAVNVWVF
YRITGGMFNPAVTLGLVLVGAVSPLRGLLIFPTQLVAAIAAAAVTDALLPGPLLVANKLSSGTSISRGLFIEMFL
TAQLVITVYFLAVEKHRATYLAPLGIGLAVFIAHICGTNFTGTSINPARSFGPAVVTDFTGYQWIYWLGPFLGSF
LAFVVYTILKWMEYHTANPGQDDDTAVKKTPAGLAVATNDRQYRNGSESGKNRSPSEPRDSGIAHTSSAPQGFAA
V*

>Trivi_8824

MDLAAFDGSFAPGVRPHAVRLTPWYRSRDYYIGQWLDLSVWKSAVVEFIATCCMVFLSGQITATLESYGTPQVGG
YIGISNIILLSTFIYATAPASGGHLNPMISFSAILTGLCSVPRGILYMCGQTLGGALAGGILLGVWGPERATSLK
GGGCWYDPSQANPGQIYLNEVFASFVLLFLSFGVGLDPRQAALFGPRMGPLLVGASLGLVSFSTSGIIPGYAGAQ
MNPSRCLAFGIARQNMAYQWIWWFGPAVGGLMMGVLYNLIPPHHTELSKQKSKESHSSSIVGHTEIPTV*

>Trivirens_160074

MAPLLPHANFWTSRHDAEALPSPTNVIPFAGRIGANQEFSLEKNNCTQIELLQKFPDAAPWIPLRDSLSLRPLLE
AVLWKAAVVEAIGTCLLVYLTCFVAVGLGQMVNVFASGALVPSLLGGLTAILILPLFIFATGPVSGAHLNPAITF
ATFFARLATLPRCILYVGFQTFGGAMAGLLLRASFDTRSFSVPGCYFDSTIVSTGSAFAIEFITDFALIFLSFGV
GLDPRQRSVFGPALGPIFVGLVLGMCTFVTGFSRVGYTGFSGNPARCFGAMVGSHFAPYHWIYWVAPLSASAIHG
MVYYLVPPYSRTRASCVSGGT*

>Trivirens_87413

MREPFSEFFGVMILVLFGDGSVAQVVLGKGAKGDWNNINWGWALGVMLGVYCGGVSGAHLNPAVTLANCIFRKFP
WKKLPIYALAQLLGAMVASLIVYGNYKSAIDVFEGGHGIRTVGLDTSTAGIFCTYPAPFLTKSGQFFDEFIGSSI
LMFCLYALLDDGNIGAGNLTPLGLFFVIYGIGACFGSNTGYAINPARDLGPRIMSHAVGYGHQVWTAGDYYFWIP
VIAPFLGCTFGGFLYDAFIYTGDSPINAPYMGLTRFMGVRAKAGRPAMV*

>Trivirens_140684

MELLSPPVEEEQLAWSKIRSNCQDFFSEFLGTMTLILFGDGVVAQVVLSGGTKGDYQSISWGWGIAVMLGVYVGG
KSGGHLNPAVTFANCLYRGHPWRKLPVYALAQLLGAMTGAAIVYANYKSAFDMFEGGAGIRTVTGPTATAGVFCT
YPAPFMTRTGMFFSEFIASSILMFCIFALADPNNIGAGNLMPLCLFFLIFGIGACFGWETGYAINLARDFGPRLV
SFMIGYGHEVWSAGGYYFWIPMVAPFCGCAFGGFLYDVFIFTGNSPINTPMLGLQRLMRPRKSVWSNTHPSAIEA
KV*

>Trivirens_41159

MSSSRRGLNESREDQDRGSLRRRSRASRRSTFRSAAPISTAGQGTQFNLAGPSDNLQLRSAHEPFVHPGYSDLNP
SYEQPNNAKPIWSLAKPLPRVVRPGMVPTKNELLENCVNAELPAENSQNLGLDVDPNEIEKGRIEKSADVRKMGA
QVTDARLQRENNFIKTILAADAETTSNGVPQLVKTRSSQRRATIQRSSIQSPLYTVQEGLSEHTSEQNKHSHESQ
RSEQGGEEFDPGQDELIEGNRSLETLRLDQDAYPEDLHPLVQELVEDEIHNNHTVWSVIRTHHREALAESLAVFV

QLTVGFCADLSVTVAKAGNPNTTDWAWGFATMIGIYISGGVSGAHLNPTITTMLWFFRGFPKRKMPEYFLAQFLG
AFCACFVAYGVYYVSIKHYLLTGADDDIINCFVTSQRSSYINAPTALFNEFIGTMCLTIVVLALGDDQNAPPGAG
MNSLIIGLIITCLSMSFANQTGAALNPSRDFGPRLALLALGYTSELFTNPYWFYGPWAGTLLGSFMGAFLYDFMI
FTGGESPINYPLERTQRALHKSHMKWQRRLRLTPKQEEDTVV*

>Trivirens_66182

MAPTEQLNRLHTHDLELGTTDAVQKHITRNVVPPRRVSQRRLDFEHRRPRWVRECIAEATGVFMYVLPGIGAIAS
FTVNATNPVGSTAFGSLFSIGFAFALGIAFAIITCAPTSGGHFSPAVTICLCIWQGFPLKKVPHYILSQLFGGFI
AALVLMGIYHQQLEEMKEVLLAAGKPLVANGAPASVLCSFPNPGQSMGYVFMTEFFCDCFVGLIIWACLDPANPF
VSPSLAPLLIGLAYGAMAWAFGANTLTMNMARDFGPRVVAAIFYGREAFSYMNYAAIGIFTSIPATLISSAFYEF
VMRDSLSVIGTGHAVHADGDEALVRHITRTTTVEGMEERSGEYKS*

>Trivirens_228188

MHRIASPRAPREGIRNEIVVVFGEFCGTFMFLLMSFIGAQAAIENNDPGNPNAPLFPFSLLYIAASFGSALAVNV
WVFYRVTGGMFNPAVTLGLVLVGAVKPLRGLFIFPTQIVAGIAAAAVTDALLPGPLLVANKLSSGTSISRGLFIE
MFLTSQLVITVYFLAVEKHRATFLAPLGIGLAVFIAHICGTNFTGTGINPARSFGPAVVTDFTGYQWIYWVGPFL
GSLLAFAVYTILKWLEYHTANPGQDDDSTAKKTPAGFSIPTNDRQFGNSDAAKHRPPSEPRDSGVAQTNSTPQGF
QAV*

>Trivirens_6760

FKTALVVGLGEFCGTFMFLLLSFMGAQAALDNRPDGGKLDASTLLYIASSFGTALAVNVWVFYRVTGGMFNPAVT
LGLVLVGAVKPLRALIIVPMQIAAGIAAAATVSGLLPGPLGVTNSLGSNTSIVQGLFIEMFLTAQLVLTVYFLAV
EKHRATFLAPVGIGVSVFIAHMAGTNFTGTGINPVRSFGPAVVTGSFRGYHWIYWLGPCLGALLSFSVYSLLKGL
EYQIANPGQD

>Necha2_72760

MRMAGPVSQTTSYNGDVESRPPVELDSPEPTAPRYRSYSHPFAGRLGANQAFTIDRRTSADEKFLEKEPDATPHM
SFRELLDCRPILSPYLWKAALIEGMGTLMQAYITIWIGISPPRLPTPPTAQLGNFDNAAFIGPLIGGITNIFFIS
LFISCFGPVSGAHFNPLITFATFCARLCSLPRLILYVSAQIGGGALAGLLVRASYGTREFKVGGCWLDPDIVPIR
EVFVVELIAATILLFLAFGLGLDPRQAQIVGPTLAPFLVGLASGTLAFSTGFTRYGYGGAGLNPARCMGAFVGTR
FPTWHWIHWVGDGIACIIHGLVYYFVPPWTKEN*

>Necha2_67872

MTASFNQDSISESSFNSPSSPAKVEMVQTKALKELPSDSTLQPHVGEREEVLLWSRVRETCQDAFSEFFGTFVMI
LFGDGVVAQVVLSRGTKGDYQSISWGWGLGVMLGVYVGGKSGGHLNPAVTLANCIFRGHPWRKLPIYAIAQTLGA
MAAAAVVYGNYKSAINAYEGGPGIRTVTGENATAGIFCTYPAAFMTRTGMVFSEFIASTILQFVIFALADSTNIG
AGPLMPLALFFLIFGIGACWGWETGYAINLARDFGPRLVSYMIGYGTEVWSAGGYYFWIPMVIPFLGTSFGGFLY
DTFMYTGPSPMNTPYMGLKRLVTPRRSVWSNTYDRALDSQV*

>Necha2_90431

MARLASFRNSVVIVLGEFCGTFMFLLLSFIGAQTALVTNNPSDPSAPLEPFSLMYIAASFGTALAVNVWIFYRVS
GGMFNPAVTLGLMLVGAVKPLNGLLIVPTQLVAAIAAAAVTDGLTPGPLLVANALGNGTSKAQGVFMEMFLTAQL
VLTVYFLAVEKHRSTYLAPIGIGISVFISHICGTNWTGTSINPARSFGPAVVTGFVGYHWIYWVGPFMGTLLAFG
CYKTFKWLEYHTANPGQDDDDVEKGRHHHLGFHSHAHEKAAMPQSQVDTIPPKDASPHQRNDSMIDGQMSPVAP*

>Necha2_51592

MATSDEAGVLDGPHHQQYQLSPVGRHLVAASGEFVGTFFFLYFGYAGNLMAALQAPDTAPNGGLASTTDIWIAVS
YGFSLLVNAWAFYRISGGLFNPAVSLGLCVGGQLSWTRAAFLFPAQVLGSICAGGLVDAMFPGRVEQANTLLGLN
TSIAQGVFLEMFFTAQLVFVVLMLAAEKSRDTFLAPVGIGLALFVALIPGVSVTGGSANPVRSFGCAVAGASFPG
YHWIYWVGPALGATLAALYYRLVKRLHYEEANPGQDSPHEV*

>Necha2_93338

MNQQLAKQQQHVTRSARSTPAANVSAYSPPGEAISSPNNESVPFAPPTSPLDQQPSRTSEADTIVPLQPAPSHEP
IRSRPGSRTGAYATPKGAARFSHDVDPLQHSGGSMKSQRRRFREDWDFDQEPRDHPTLSDYERYWRNEGRPERYT
SRRSNCPSPAFMNRRRTAAHDSDEDLYNDDLRYAKDPFEITRGRRLTDEEMGYHPGRINHQRRPSSSHGVNLASG
RLEWNNLTAHEKAQVMRLPLTQWMNSEFKNHFVASLGEFIGTTMFLFFAFAGTEVANIQSNTSAKTTTGESTGFS
VSTLLYISIIFGFSLMVNVWVFFRISGGLFNPAVTIAMLMVKAISLTRAICLFISQILGAMLASVVVLYLFPEAF
NVRTTLGGGASLVQGVFIEAILTAELVFTIFMLAKEKHRATFIAPVGIGLALFIAEMVGVQFTGGSLNPARSFGP
CVVTGTFDTEHWIYWVGPFIGSLIAVAFYWFIKTLEYEMANPGADGDDANDPTKNPEKRAEIQASKPPSIQFGLG
KSPSIRS*

>Fusacuminatum_CBMG010003518.1

MGTTAQRTASPSQDVESRPSFEPSGPRYQSYSQPFAGRLGANQAYVVDGVTSEDEKLLQHQPDATPHMSFRELMD
CRPITNINLWKAALIEGIGTLLFVYITIWANSSPNVIPASPTQQLGNFDNAAFIGPLIGGILNLVFITLFITSFG
AISGAHFNPLITFATFCARLCSLPRMILYIAAQVGGSVLAGLLVRASWGGRDFKTGGCWLFTEVVPPREIFVVEL
VSATILLFLAFGVGLDPRQAKVIGPALGPLLVGLSVGTMSFASAFTRYGYGGAGLNPARCMGAFVGSRFPTWHWI
HWVADGIACIIHGVCYYFIPPWTEVRQ*

>Fusacuminatum_CBMG010003683.1

MPISTMNDSISESSVRRSSVHKSSIPTKVEMSQHEKYSEATSEAPTISPPPEQYAWSRVREYCQDAFSEFFGTFI
LLLFGDGVVAQVVLSRGTKGDYQSISWGWGLGVMLGVYVGGKSGGHLNPAVTLANCLFRGHPWRKFPIYAIAQIL

GAMAAAAVVYGNYKSAFDVYEGGPGIRTVVGENATAGIFCTYPAEFMTRTGMFFSEFVASTILQFVIFAMADSAN
IGAGPLMPLGLFFLIFGIGCFGWETGYAINLARDFGPRLVSYMIGYGSEVWSAGGYYFWIPMVAPFFGCTFGGFL
YDVFIYTGPSPINTPGMGFGRLLSPRRSTWSNTYSATGSPV*
>Fusacuminatum_CBMG010000926.1
MVQFTQRTNSGMSGLPTEEAVADTKARTPIPDRIKNAIVIIIGEFCGTFMFLLLSFIGAQTALVTNNPSNPSAAL
EPFSLMYIAASFGTALAVNVWIFYRVSGGMFNPAVTLGLVLVGAVPPLHAAAIIPTQLIAAITAAGITKALIPGP
LLVTNALGNGTSIAQGVFIEMFLTAQLVLTVYFLAVEKHRSTHLAPIGIGISVFIAHICATNWTGTSINPARSFG
PSVVAGFNGYDWIYYVGPFMGSLLAFGCYKIFKVLEYQTANPGQDDDDLERNSGGHHFFKREKEEPVSHSHTDMI
EPKDHGVPQRNDSVIDGQMPPV*
>Fusacuminatum_CBMG010000883.1
MSSTADIHGTPVRNGHNGHHHHSRSRSHSNRNRNSNSRTLYHEKHQRSHLSDFQKHLVAASGEFAGTFLFLYFAY
AGNIVAVLQEPMAARDGSLANSTLMYIAMAYGFSLLVNVWAFFRISGGLFNPAVTFGLCLAGQLPWIRALFLFPA
QITAAMCAGGLVDAMFPGNASLANTVLGPNTSVVQGVFLEMFFTAQLTFVVLMLAAEKSRDTFLAPVGIGLALFV
ALIPGVFVTGGSANPVRSFGCAVGAREFPSYHWIYWIGPLLGAALAAGYFRLVKMMHYEEANPGQDSPVDV*
>Fusacuminatum_CBMG010000225.1
MSSSILSRSARNTPTMAGSAFSPPGEPSAAASSSSQERVPFMRENSPEADTLPSNPAATPVVTHGDNIPFSRTSE
ADTVVPLQPVISQDATSFRPGSRTGNFVTPKATRFSQDGGEPVYYSGSIKSNRRRPRERDEYDFDQAADYPNQSD
YERYWRTESRNDRDRYAGRRGPYPPPTFMNRRRVPPHDSDDEFYSSDDPRMPPNGGRRMVDEEMGAYPARPFNLH
RTSTLNGFNITTGKLEWNNLSRKEKSEIMRLPLTQWMNSNFKNHFVAGIGELIGTTMFLFFAFAGTEVANIQAET
NNRTTTGEATGGLNVSKLLYISIIFGFSLMVNVWVFFRISGGLFNPAVTIAMLMVKAISMTRAICLFLAQILGSM
LASVIVRYLFPETFNVRTTLGGGASLVQGVFIEALLTAELVFTIFMLAKEKHRATFIAPVGIGLALFIAEMVGVQ
FTGGSLNPARSFGPCVITGTFDSEHWIYWVGPFIGSLIAVCFYWFIKTLEYEMANPGADGDDLNDPTKNPEKRAE
VQATTTKIPTTAFGGGKTPSILS*
>Fusacuminatum_CBMG010002146.1
MPGPHEQNHDYFSKPTTPSTPGPAHLGNVASRIPSTISDRETGHINRNKSTHSKRHRAIHGLPTSFVSRMSKRPA
SSRRFTRMSSSGSAARPVAPQYPSQFREYAPAEEGYYDENPWFGESEKKPIFSLGKPLPHKVRRKVIKPIRPDGT
VDEEMAVVKEDSANEFPSRYNSYTTAGQPYRAETQSSLTTRDTQNQQSRTTAAGVAHNEKRNDAGQPVYDYVPGQ
VTPATPTHRDQPASRVQSRQQNEHEQPDFKVDGEPLGQQERSAVETGERDPNEMRNWWARLRAKHPEPLAEFLAT
CVAIFLGLTGTLSVNLSAKQSQGYGTYETSCWAWGFAWMFGIYLGGGVSGAHMNPAISISLSLFRGFPWKQCLVY
IIAQFLASIVAGALAYGIYADSIHFVDPDMTGMSMTFFSTPREWVSLSSAFFNQVVGSAIMMIAVFALGDDQNNP
PGAGMHALVLGFLVTTLKFTLGYNIGSALNPASDFGPRVIAYAVGYRESNVFQSGWWFYGPWAATLIGSVLGCAL
YDGFVFVGSESPINFRFNKEIKKRAKKLLN*

>Fusavenaceum_JQGD01000013.1
MGTTAQRTASPSQDVESRPSFEPSGPRYQSNSQPFAGRLGANQAYVVDGVTSEDEKLLQHQPDATPHMSFRELMD
CRPITNINLWKAALIEGIGTLLFVYITIWANSSPNVIPALPTQQLGNFDNAAFIGPLIGGILNLVFITLFITSFG
AISGAHFNPLITFATFCARLCSLPRMILYIAAQVGGSALAGLLVRASCGGRDFKTGGCWLFTEVVPPREIFVVEL
VSATILLFLAFGVGLDPRQAKVIGPALGPLLVGLSVGTMSFASAFTRYGYGGAGLNPARCMGAFVGSRFPTWHWI
HWVADGIACIIHGVCYYFIPPWTEVRQ*
>Fusavenaceum_KIL87348.1
MPISTMNDSISDSSIRRSSINKSSIPTKVEMSQHEKYSEATSEAPTISPPPEQYAWSRVREYCQDAFSEFFGTFI
LLLFGDGVVAQVVLSRGTKGDYQSISWGWGLGVMLGVYVGGKSGGHLNPAVTLANCLFRGHPWRKFPVYAIAQIL
GAMAAAAVVYGNYKSAFDAYEGGPGIRTVIGENATAGVFCTYPAEFMTRTGMFFSEFVASTILQFVIFAMADSNN
IGAGPLMPLGLFFLIFGIGACFGWETGYAINLARDFGPRLVSYMIGYGSEVWSAGGYYFWIPMVAPFFGCAFGGF
LYDVFIYTGPSPINTPGMGFGRLVSPRRSTWSNTYSATGSPV*
>Fusavenaceum_KIL88092.1
MVQFTQRTNSNMSGLPTEEAVADTRERTPIPDRIKNAIIIVIGEFCGTFMFLLLSFIGAQTALVTNNPSNPSAGL
EPFSLMYIAASFGTALAVNVWIFYRVSGGMFNPAVTLGLVLVGAVPPLYAVAIIPTQLVAAITAAGITKALIPGP
LLVTNALGNGTSIAQGVFIEMFLTAQLVLTVYFLAVEKHRSTHLAPIGIGISVFIAHICATNWTGTSINPARSFG
PSVVAGFNGYDWIYYIGPFMGSLLAFGCYKIFKVLEYQTANPGQDDDDLERNSGGHHFFKHEKEEPVSHSHTDMI
EPKDHGVPQRNDSVIDGQMPPV*
>Fusavenaceum_KIL87632.1
MSSTADIHSTPVRNGHNGHHRSRSRSHGNRNSRTLYHQKHQRGHLSEIQKHLVAASGEFAGTFLFLYFAYAGNIV
AVLQEPMAARDGSISNSTLMYIAMSYGFSLLVNVWTFFRISGGLFNPAVTFGLCLAGQLPWIRALFLFPAQIIAA
MCAGGLVDAMFPGNLSLANTVLGPDTSVVQGVFLEMFFTAQLVFVVLMLAAEKSRDTFMAPIGIGLALFVALIPG
VFVTGGSANPVRSFGCAVGAREFPSYHWIYWIGPLLGAALAAGYFRLVKMMHYEEANPGQDSPVDV*
>Fusavenaceum_KIL89865.1
MSSSILSRSARNTPTMAGSAFSPPGEPSAAASSSSQERVPFMRENSPEADTLPSNPAATPVVTHGDNIPFNRTSE
ADTVVPLQPVISQDATSFRPGSRTGNFVTPRATRFSQDGGEPVHYSGSVKSNRRRPRERDEYDFDQAADYPSQSD
YERYWRTESRNDRDRYAGRRGPYPPPTFMNRRRVPPHDSDDEFYSSDDPRMPPNGGRRVMDEEMGVYPARPSHLH
RSSTLNGFNITTGKLEWNNLSRKEKSQIMRLPLTQWMNSNFKNHFVAGIGELIGTTMFLFFAFAGTEVANIQAET
NNRTTTGEATGGLNVSKLLYISIIFGFSLMVNVWVFFRISGGLFNPAVTIAMLMVKAISMTRAICLFLAQILGSM
LASVIVRYLFPETFNVRTTLGGGASLVQGVFIEALLTAELVFTIFMLAKEKHRATFIAPVGIGLALFIAEMVGVQ

FTGGSLNPARSFGPCVITGTFDSEHWIYWVGPFIGSLIAVCFYWFIKTLEYEMANPGADGDDLNDPTKNPEKRAE
VQATTTKIPTTAFGGGKTPSILS*
>Fusavenaceum_JPYM01000003.1
MPGPHEQNHDYFSKPTTPSTPGPAHLGNVASRIPSTISDRETGHIDRNKSTHSKRHRAIHGLPSFVSRMSKRPAS
SRRFTRMSSSGSAARPVAPQHPSQFREYAPAEEGYYEENPWFGESEKKPIFSLGKPLPHKVRRKVIKPIRPDGTV
DEEMAVVKEDTANEFPSRYNSYTTAGQPYRAETQSSLTTRDTQNQQSRTTAAGVAHNEKRNDAGQPVYDYVPGQA
TPATPTHRDQPASRAQSHQQNEHSQPDFKVDGEPLGQQERSSVEAGDKDPNEMRNWWARLRAKHPEPLAEFLATC
VAIFLGLTATLSVNLSAKQSQGYGTYETSCWAWGFAWMFGIYLGGGVSGAHMNPAISISLSLFRGFPWKQCLIYI
IAQFVASIVAGALAYGIYADSIHFVDPDMTGMSMTFFSTPREWVSLSSAFFNQVVGSAIMMIAVFALGDDQNNPP
GAGMHALVLGFLVTTLKFTLGYNIGSALNPASDFGPRVIAYAVGYRESNVFHSGWWFYGPWAATLIGSVLGCALY
DGFVFVGSESPINFRFNKEIKKRAKKLLN*

>Fuscircinata_AYJV01002164.1
MAVTNRRKSRDSGDVEARPSSEPSGPHYRRYSQPFAGRLGANQAYVVEGGTSEDDHVLHHAPDATPHMSFRELMD
MRPIKNLDLWKAALIEGIGTLLFVYITIWVSISPDIAPAAPTQRFGSFDNAAFLGPLIGGITNLIFITLFITCFG
PISGAHFNPLITFATFCARLCSLPRLILYVAAQIGGGALAGLLVRASWGGRDFKVGGCWLFTDIVPPKEIFVVEL
VSATLLLFLAFGVGLDPRQAKIIGPALGPFMVGLSVGTMSFASAFARYGYGGAGMNPARCMGAFVGSRFPSWHWI
HWVADGTACIIHGVCYYFIPPWTEVRQ*
>Fuscircinata_AYJV01002992.1
MPIPTMNDSISESSVHKSSIPTKVEMSQNEKYSEAPSEPPTIPPPPEQYAWSRVREYCQDAFSEFFGTFILLLFG
DGVVAQVVLSRGTKGDYQSISWGWGLGVMLGVYVGGKSGGHLNPAVTLANCIFRGHPWRKFPVYAVAQVLGAMCA
AAVVYGNYKSAFDAYEGGPGIRTVIGENATAGVFCTYPAEFMTRTGMFFSEFIASTILQFVIFAMADSANIGAGP
LMPLGLFFLIFGIGACFGWETGYAINLARDFGPRLVSYMIGYGSEVWSAGGYYFWIPMVAPFMGCAFGGLLYDVF
IYTGPSPINTPGMGLPRLLSPRRSTWSNTYSASSPV*
>Fuscircinata_AYJV01001480.1
MVQFTRADTGMSGLPTEEAVADRRAGSPIPNRVRNAIVIVLGEFCGTFMFLLLSFIGAQTALVTNNPTNSTAPLE
PFSLMYIAASFGTALAVNVWIFYRVSGGMFNPAVTLGLVLVGAVPPLHALAIIPTQLVAAIAAAGVTDGLIPGPL
LVTNALGNGTSIAQGVFMEMFLTAQLVLTVYFLAVEKHRSTHLAPIGIGISVFIAHICLTNWTGTSINPARSFGP
SVVAGFHGYDWIYYLGPFMGSFLAFGCYKIFKVLEYQTANPGQDDDDLERGSKHHFFDHHEKEPIAHSQTDTLEP
KDHGAAPRNDSVIDGQMSHA*
>Fuscircinata_AYJV01003071.1
MANAAEVQGANGHQHHHKQQRTHLSEFGTHMVAASGEFVGTFFFLYFGYAGNIIAVLQEPATGPNGTLANNTIIW
IAMAYGFSLLVNVWAFYRISGGLFNPAVTFGLCLAGQLPWMRALYLFPAQLVASMCAGGLVEAMFPGSASQANTT
LGPNTSIAQGVFLEMFFTAQLVFVVLMLAAEKSRDTFLAPIGIGLSVFVALIPGVFVTGGSLNPVRSFGCAVGGR
DFPGYHWLYWVGPLLGGALAAGYFRLVKMMHYEEANPGQDSPVDV*
>Fuscircinata_AYJV01000611.1
MSSSIQSRSARSTPAINASAFNPPDTNCSSNERVPFMRDNSPEADSLPSNPALTPVITHGDNPPASNRNSEADTV
VPLQPVLSQDPSRVRPGSRTGNFVTPRTARFSQDGGEPLQYSGGSMKSSRRRYRGEDYDFDQAADYPTVSDYERY
WRNEGRHDRYTARRGPYPPPTFMNRRRAHPHDSDEEFYSSDDPRMTPSGGRRMMDEEMGYPHRPIHHRRDSTLNG
FNISTGKLEWSDLSSQRKSEIMRLPLTQWMNSNFKNHFVASLGELIGTTMFLFFAFAGTEVANIQAQTDSKTTTG
ESTGSLNVSKLLYISIIFGFSLMVNVWVFFRISGGLFNPAVTIAMLMVKAISMTRAICLFLSQILGSMLASVMVR
YLFPETFNVRTTLGGGASLVQGVFIEALLTAELVFTIFMLAKEKHRATFIAPVGIGLALFIAEMVGVQFTGGSLN
PARSFGPCVITGTFDSEHWIYWVGPGIGSLIAVCFYWFIKTLEYEMANPGADGDDLNDPTKNPEKRAEVQSNAVP
SIAFGGGKTPSIRS*
>Fuscircinatum_JRVE01000048.1
MTGHQEQNHDYFSKPETPSTPGQVHLGNVASRIPSTISDRETGQADRAKSTHSRRRGLHGLPSSFMSRMSKRPIS
SRRFTRMSSGHSTTRPTVQQHASQHHTYAPAEEGYYEENPWFGEAGKKPIFSLGKPLPHRVRRKVVKPIRPDGKV
DEEMAIVKEDSEDTPAGYASRTTSGQPYRMHTQTSIASRDMQNQQSRTTAAGVAHNEKRNDAGQPVFDYIPGEAT
PAATPSHRDPASRVASHQQSNNQPDYKIDSEPLGQQEKPEVEAGETDPNEMRNWWARIRAKHPEPLAEFLATSVA
IFLGLTGTLSVNLSAKQSQSYGTYETSCWAWGFAWMFGIYLGGGVSGAHMNPAISISLSLFRGFPWRQCFIYILA
QFIASIVAGALAYGIYADSIHYVDPEMDGMSKTFFSTPREWVSLQSAFFNQVVGSAIMMIAVFALGDDQNNPPGA
GMHALVLGFLVTTLKFTLGYNIGSALNPASDFGPRVVAYAVGYRESNVFYSGWWFYGPWAATLIGSVVGCALYDG
FVFVGSESPINFRFSKEIKHRAN*

>Fusculmorum_CBMH010000861.1
MGITMRRSNSHEARDGEIESRPSFEPTGPRFQSNDLPFAGRLGANQAYVIDGATSEDEKLLEHQPDATPHMSFRE
LVDLRPITNPDLWKAAMIEGIGTLLSVFVTIWASSSPDVIPAQPTRQLGNFDNAAFIGPLVGGIINVILITLFIT
CFGAVSGAHFNPLITFATFCARLCSLPRMILYIPAQVGGAALAGLLVRASWGGRDFKAGGCWLFTEVVPAREAFV
IELVFSTVLLFLAFGVGLDPRQAKIIGPALGPFLVGLSVGTMSFGSAFTRYGYGGAGMNPARCMGAFVGSRFPSW
HWIHWVANFIACIAHGICYYFIPPWTQVKG*
>Fusculmorum_CBMH010000823.1
MPISTINDSISESSVHKSSIPTKVEMSQNEKYSEAPSEAPTIPPPPEQYAWSRIRENCQDAFSEFFGTFVLLLFG
DGVVAQVVLSRGTKGDYQSISWGWGLGVMLGVYVGGKSGGHLNPAVTLANCLFRGHPWRKFPIYAVAQVLGAMAA

AAVVYGNYKSAIDAYEGGPGIRTVIGENATAGVFCTYPAEFMTRTGMFFSEFIASTILQFVIFAMADSANIGAGP
LMPLGLFFLIFGIGACFGWETGYAINLARDFGPRLVSYMLGYGSEVWSAGGYYFWVSPSAYCKVNISTDSSPDSY
GRSILWLCFWWFSV*
>Fusculmorum_CBMH010000147.1
MVQFGSRANTNMTGLPTEQAVADRRVGNPKRDRMRNAIVIVIGEFCGTFMFLLLSFIGTALVTNSPSDAGSPLLP
FSLMYIAASFGTALAVNVWIFYRVSGGMFNPAVTLGLVLVGAVTPIHALLIIPTQLVAAITAAGITDALLPGKLL
VTNALGNGTSVAQGVFIEMFLTAQLVLTVYFLAVEKHRSTHLAPIGIGISVFIAHICATNWTGTSINPARSFGPS
VVAGFHGYDWIYYIGPFMGSLLAFGCYKIFKVLEYQTANPGQDDDNLDRSGHHHFFGHGKEPMPRTHTDTIEPKD
HGVPQRNDSVIDDQMV*
>Fusculmorum_CBMH010000781.1
MADTYGMNGHNGHTKHRRSSSMNGRNRLYAQQKPQRTTHLSEFGKHMVAASGEFVGTFLFLYFGYAGNIVAVLQE
PISGPNGTLANNTVMYVAMAYGFSLLVNVWTFYRISGGLFNPAVTFGLCLSGQLPWIRALFLFPSQIIAAMCAGG
LVNAMFPGSASIANTTLGPDTSIAQGVFLEMFFTAQLVFVVLMLAAEKSRDTFLAPVGIGLALFVALIPGVFVTG
GSANPVRSFGCAVGSRDFPGYHWIYWVGPLLGAALAAGYFRLVKMMHYEEANPGQDSPVDV*
>Fusculmorum_CBMH010001238.1
MSSSILNRSARSTPAGANPAFNPPAEASSSSTQSGVPYIRENSPEAESIPSNPAMTPIITHGDSLASPGGRNSEA
DTVVPLQPAVSQDAIRSRPGSRTGNFVTPKAARFSQDGAEPTMQYSSGSVKSARRRTREREDYDFDQAADYAPMS
EYERYYRNEGRNDRDRYTRRGPYPPPTFMNRRRAPPLDSDEEFYSSDDPRIPPNDRRRMMDEEAGYPGRPHAFRR
TSTLNGFNITTGKLQWNELSRQEKSEIMRLPLTQWMNSNFKNHFVAGVGEFIGTTMFLFFAFAGTEVANIQADTT
NRTTTGESTGSLNVSKLLYISIIFGFSLMVNVWVFFRISGGLFNPAVTMAMLMVKAISVTRAIVLFLAQILGSML
ASVVVRYLFPETFNVRTTLGGGASLVQGVFIEALLTAELVFTIFMLAKEKHRATFIAPVGIGLALFIAEMVGVQF
TGGSLNPARSFGPCVITGSFDTEHWIYWVGPAIGSLIAVCFYWFIKTLEYEMANPGADGDDLNDPTKNPEKRAEI
QASKPVPTAAFGSGKTASILS*
>Fusculmorum_CBMH010000770.1
MTGPQDQNHDYFSKPTTPSTPGQAHLGNVANRIPSTIPDRESGTERAKSTHSRHRVLHGLPSTFMSYMSRRPVAS
SRGFSRMSSEGTASRPTAPQHSSHFHHYAPAEDGYYQENPWFGEADKKPIFSLGKPLPHKVRRKVIKPIRPDGTK
VDEEMAIVKEETINEPHPGYASRTTSGQPYRVETQSSLPSRDIQRQQSRTTAAGVAHNDRRNDAGQPVFEYIPGE
ATPTPGHRDPASRVQSKQDDGHSPPDFKVDGEPLGQQEKPCVESGDTDANEMRNWWARLRARHPEPLAEFLATAV
AIFLGLTGTLSVNLSAKQSQPYGTYETSCWAWGFAWMFGIYLGGGVSGAHMNPAISVSLSIFRGFPWRQCTIYVF
VQFIASIVAGALAYAMYADSINHVDPDMTKMSMTFFSTPREWVTLKSAFFNQVVGSAIMMIAVFALGDDQNNPPG
AGMHALVLGFLVTTLKFTLGYNIGSALNPASDFGPRVIAYAVGFRGDNVFHSGWWFYGPWAATLIGSILGCTLYD
GFVFVGSESPINFRLDKRVKKLLN*

>Fusequiseti_CBMI010004636.1
MATTARQTSSNGRDIESRPSIESLNPRYQSNNLPFAGRLGANQVYVVDGTTLEDQKILEHQPDATPHMSFTELMD
MRTIANINLWKAALIEGIGTLMFVYITSWANASPDVIPTRPTAQLGNFDNAAFLGPLIGGILNFLFVTLFIASFG
AISGAHFNPLITFATFCARLCSLPRMVLYISAQVGGSALAGLLVRASWGGRDFKVGGCWLYTDVVPPREIFVVEL
VSATLLLFLAFGVGLDPRQAKVIGPSLGPFLVGLSVGTMSFATAFTRYGYGGAGLNPARCMGAFVGSRFPGWHWI
HWVADGIACIVHGLCYYFIPPWAEVRN*
>Fusequiseti_CBMI010002872.1
MPIDTIHDSISESSVHKSSIPTKVEMSQTEKYSEATSEAPTIPPPPEQYAWSRMREYCQDAFSEFFGTFTLLLFG
DGVVAQVVLSRGTKGDYQSISWGWGLGVMLGVYVGGKSGGHLNPAVTLANCLFRGHPWRKFPVYMIAQILGAMAA
AFIVYGNYKSAIDAYEGVGVRTVIGENATAGIFCTYPAEFMTRTGMFFSEFIASTILQFVIFAMADSANIGAGPL
MPLGLFFLIFGIGACFGWETGYAINLARDFGPRLVSYMLGYGSEVWSAGGYYFWIPMVAPFLGCAFGALLYDVFI
YTGPSPINTPGMGLPRLLSPRRSTWSNTYSANSPV*
>Fusequiseti_CBMI010000078.1
MVQFTSRVDTGMTGLPTEQAAVDRRVGSPMGDRVRNALVIVLGEFAGTFMFLLLSFIGAQTALVTNNPSDPTAPL
LPFSLMYIAASFGTALAVNVWIFYRVSGGMFNPAVVGLVLVGAVPPLHALAIIPTQLVAAMAAAGITDALLPGPL
LVTNALGNGTSVAQGVFIEMFLTAQLVLTVYFLAVEKHRSTHLAPIGIGISVFIAHICATNWTGTSINPARSFGP
SVVAGFHGYDWIYYIGPFMGSLLAFGCYKIFKVLEYQTANPGQDDDDIERASRHHFFHHGKEPVSHSHTDTIEPK
DHGVPQRNDSVIDGQMAPV*
>Fusequiseti_CBMI010004009.1
MGSTDKRNGNGNSGHQRHHLSTFSAHLVAALGEFVGTFFFLYFGYAGNLMVTPQEPYPEVDGGSATTTSIYIALA
YGFSLLVNVWAFYRVSGGLFNPAVTLGLCIAGQLPWLRAVFLVPAQLLGSMCAGGLVDVMFPGSVAQANTVLGPH
TSIVQGVFLEMFFTAQLIYVVLMLAAEKSKDTFMAPIGIGLALFVALIPGVPVTGGSANPARSFGCAVAGTDFPG
YHWIYWVGPCLGAGLAAGYFRLVKRLHYEEANPGQDDAYDA*
>Fusequiseti_CBMI010004298.1
IVFRSARSTPAVANSAFSPPAEAPSSSTQERGSIVRDNSPEAESLPSNPAVTPIITHGDNLASPNGRSSEADTVV
PLQPAVSQDAAIRSRPGSRTGNFVTPKTARFSQDGAEPVQYSGGSIKSARRRTRERDDYDFDQAADYAPMSDYER
YYRTEGRNDRERYTRRGPYPPPSHMNRRRAPPHDSDEEFYSSDDPRLPPNGGRRMMDEELGYSGRPYAFRRTSTL
NGFNITTGKLQWNELSRQEKSEIMRLPLTQWMNSNFKNHFVASMGEFIGTTMFLFFAFAGTEVANIQADTNNRTT
TGESTGSLNVSKLLYISIIFGFSLMVNVWVFFRISGGLFNPAVTMAMLMVKAISITRAICLFLAQILGSMLASVV
VRYLFPETFNVRTTLGGGASLVQGVFIEALLTAELVFTIFMLAKEKHRATFIAPVGIGLALFIAEMVGVQFTGGS

LNPARSFGPCVITGSFDSEHWIYWVGPAIGSLIAVCFYWFIKTLEYEMANPGADGDDLNDPTKNPEKRAEIQAAK
PAVVPTAAFGTGKTASILS*
>Fusequiseti_CBMI010001576.1
MPGPQEQNHDYFSKPTTPSTPGQVHLGNVANRIPSTISDREGTDRSKSTNSKRHVLHGLPTSFMSRMSRRPATSN
RGFSRMSSEGTATRPTAPQHSSHHHTYAPAEDGYYEENPWFGEQDKKPIFSLGKPLPHKVRRKVLKPIKPDGKID
EEMAIVKEEPTNEPHPGYTSRTNSGQPYRMQTQSSLQSREMQRQQSRTTASGVAHNDKRNDAGQPVFDYVPSEAA
PTPGHRDPASRVESRQDRNTSQPDYKVDGEPLGHQEKPSVEAGETDPNEMRNWWARIRAKHPEPLAEFLATAVAI
FLGLTGTLSVNLSAKQSQSYGTYETSCWAWGFAWMFGIYLGGGVSGAHMNPAISVSLSIFRGFPWKQCGIYIIAQ
FVASIAAGALAYAIYADSIHYVDPDMTGLSMTFFSTPREWVTLKSAFFNQVVGSAIMMIAVFALGDDQNNPPGAG
MHALVLGFLVTTLKFTLGYNIGSALNPASDFGPRVVAYAVGYRQENVFHSGWWFYGPWAATLIGSILGCALYDSF
VFVGSESPVNFRFGKRVKKLL*

>Fusfujikuroi_ANFV01000069.1
MAVTNRRKARDSGDVEARPSSEPSGPHYRSNSQPFAGRLGANQAYVVEGGTSEDDHLLHHAPDATPHMSFWELMD
MSPIKNLDLWKAALIEGIGTLLFVYITIWVNISPDTAPAAPTQRFGSFDNAAFLGPLIGGITNLIFITLFITSFG
AISGAHFNPLITFATFCARLCSLPRLILYVAAQLGGGALAGLLVRASWGGRDFKVGGCWLFTDIVPPREIFVVEL
VSSTLLLFLAFGVGLDPRQAKIIGPALGPFMVGLSVGTMSFASAFARYGYGGAGLNPARCMGAFVGSSFPSWHWI
HWVADGTACIIHGVCYYFIPPWTEVRQ*
>Fusfujikuroi_ANFV01000060.1
MPIPTMNDSISESSVHKSSIPTKVEMSQNEKYSEAPSEPPTIPPPPEQYAWSRVREYCQDAFSEFFGTFILLLFG
DGVVAQVVLSRGTKGDYQSISWGWGLGVMLGVYVGGKSGGHLNPAVTLANCIFRGHPWRKFPVYAVAQVLGAMCA
AAVVYGNYKSAFDAYEGGPGIRTVIGENATAGVFCTYPAEFMTRTGMFFSEFIASTILQFVIFAMADSANIGAGP
LMPLGLFFLIFGIGACFGWETGYAINLARDFGPRLVSYMIGYGSEVWSAGGYYFWVSPMPMVFFLISTDSGIQIP
MVAPFMGCAFGGLLYDVFIYTGPSPINTPGMGLPRLLSPRRSTWSNTYSASSPV*
>Fusfujikuroi_CCT62977.1
MVQFTRTDTGMSGLPTEEAVADRRAGSPIPNRVRNAIVIVLGEFCGTFMFLLLSFIGAQTALVTNNPTNSTAPLE
PFSLMYIAASFGTALAVNVWIFYRVSGGMFNPAVTLGLVLVGAVPPLHALAIIPTQLVAAIAAAGVTDGLLPGPL
LVTNALGNGTSIAQGVFMEMFLTAQLVLTVYFLAVEKHRSTHLAPIGIGISVFIAHICLTNWTGTSINPARSFGP
SVVAGFHGYDWIYYLGPFMGSFLAFGCYKIFKVLEYQTANPGQDDDDLERGSKHHFFDHHEKEPIAHSQTDTLEP
KDHGAAPRNDSVIDGQMSHA*
>Fusfujikuroi_CCT74579.1
MASAAEVQGANGFNGHHQHKRQRTHLSEVGTHMVAASGEFVGTFFFLYFGYAGNIVAVLQEPATGPNGTLTNNTI
IWIAMAYGFSLLVNVWAFYRISGGLFNPAVTFGLCLAGQLPWMRALYLFPAQLIASMCAGGLVEAMFPGSASQAN
TTLGPNTSIAQGVFLEMFFTAQLVFVVLMLAAEKSRDTFLAPIGIGLSVFVALIPGVFVTGGSLNPVRSFGCAVG
GRDFPGYHWLYWVGPLLGGALAAGYFRLVKMMHYEEANPGQDSPVDV*
>Fusfujikuroi_CCT71530.1
MSSSIQSRSARSTPAINASAFNPPEANGSSNERVPFMRDNSPEADSLPSNPALTPVITHGDNPPVSNRNSEADTV
VPLQPVISQDPSRVRPGSRTGNFVTPKTARFSQDGGEPLQYSGGSMKSSRRRYRGEDYDFDQAADYPAVSDYERY
WRNEGRNDRYTARRGPYPPPTFMNRRRAHPHDSDEEFYSSDDPRMPPTGGRRMMDEEMGYPHRPFHHRRASTLNG
FNISTGKLEWSNLSPKEKSEIMRLPLTQWMNSNFKNHFVASLGELIGTTMFLFFAFAGTEVANIQAQTDSKTTTG
ESTGSLNVSKLLYISIIFGFSLMVNVWVFFRISGGLFNPAVTIAMLMVKAISMTRAICLFLSQILGSMLASVMVR
YLFPETFNVRTTLGGGASLVQGVFIEALLTAELVFTIFMLAKEKHRATFIAPVGIGLALFIAEMVGVQFTGGSLN
PARSFGPCVITGTFDSEHWIYWVGPGIGSLIAVCFYWFIKTLEYEMANPGADGDDLNDPTKNPEKRAEVQSNAVP
SIAFGGGKTPSIRS*
>Fusfujikuroi_JRVG01000144.1
MTGHQDQNHDYFSKPATPSTPGQVHLGNVASRIPSTISDRETGQPDHAKSTHSRRRGLHGLPSSFMSRMSKRPMS
SRRFTRMSSGHSTARPTVQQHASQYHTYAPAEEGYYEENPWFGEAGKKPIFSLGKPLPHRVRRKVVKPIRPDGKV
DEEMAIVKEDSEDTPAGYASRTTSGQPYRMHTQTSIGSRDMQNQQSRTTAAGVAHNEKRNDAGQPVFDYVPGEAT
PAATPVHRDPASRVASHQQNNNQPDYKIDSEPLGQQEKPEVEAGETDPNEMRNWWARIRAKHPEPLAEFLATSVA
IFLGLTGTLSVNLSAKQSQTYGTYETSCWAWGFAWMFGIYLGGGVSGAHMNPAISISLSLFRGFPWRQCFIYILA
QFIASIVAGALAYGIYADSIHYVDPEMDGMSKTFFSTPREWVSLQSAFFNQVVGSAIMMIAVFALGDDQNNPPGA
GMHALVLGFLVTTLKFTLGYNIGSALNPASDFGPRVVAYAVGYRGSNVFHSGWWFYGPWAATLIGSVVGCALYDG
FVFVGSESPINFRFSKEIKHRAN

>Fusgraminearum_HG970333
MRRTNSHEARDGEIESRPSFEHTGPRFQSNDLPFAGRLGANQAYVIDGATSEDEKLLEHQPDATPHMSFRELIDL
RPITNIDLWKAALIEGIGTMLSVFVTIWASSSPDVIPAQPTRQLGNFDNAAFIGPLVGGIINVILITLFITSFGA
ISGAHFNPLITFATFCARLCSLPRMILYIPAQIGGAALAGLLVRASWGGRDFKTGGCWLFTEVVPAREAFVIELV
FSTVLLFLAFGVGLDPRQAKIIGPALGPFLVGLSVGTMSFGSAFTRYGYGGAGMNPARCMGAFVGSRFPSWHWIH
WVASCIACIVHGICYYFIPPWTQVKG*
>Fusgraminearum_XP_011322503.1
MPISTINDSISESSVHKSSIPTKVEMSQNEKYSEAPSEAPTIPPPPEQYAWSRIRENCQDAFSEFFGTFVLLLFG
DGVVAQVVLSRGTKGDYQSISWGWGLGVMLGVYVGGKSGGHLNPAVTLANCLFRGHPWRKFPIYAVAQVLGAMAA

AAVVYGNYKSAIDAYEGGPGIRTVIGENATAGVFCTYPAEFMTRTGMFFSEFIASTILQFVIFAMADSANIGAGP
LMPLGLFFLIFGIGACFGWETGYAINLARDFGPRLVSYMLGYGSEVWSAGGYYFWIPMVAPFFGCAFGGFLYDVF
IYTGPSPINTPGMGFGRLVSPRRSTWSNTYNANSPV*
>Fusgraminearum_XP_011316524.1
MVQFGSRANTNMTGLPTEQAVEDRRVGNPKRDRMRNALVIVLGEFCGTFMFLLLSFIGAQTALVTNSPSDAGSPL
LPFSLMYIAASFGTALAVNVWIFYRVSGGMFNPAVTLGLVLVGAVTPIHALLIIPTQLVAAITAAGITDALLPGK
LLVTNALGNGTSVAQGVFIEMFLTSQLVLTVYFLAVEKHRSTHLAPIGIGISVFIAHICATNWTGTSINPARSFG
PSVVAGFHGYDWIYYIGPFMGSLLAFGCYKIFKVLEYQTANPGQDDDNLDRSGHHHFFGHRKEPMPHTHTDNIEP
KDHGVPQRNDSVIDDQMV*
>Fusgraminearum_XP_011322016.1
MADTYGMNGHNGHVKDRRSSSMNGRNRLYAQQEPQRTTHLSEFGKHMVAASGEFVGTFLFLYFGYAGNIVAVLQE
PISGPNGTLANNTVMYIAMAYGFSLLVNVWTFYRISGGLFNPAVTFGLCLSGQLPWIRALFLFPSQIIAAMCAGG
LVNAMFPGSASIANTTLGPNTSIAQGVFLEMFFTAQLVFVVLMLAAEKSRDTFLAPVGIGLALFVALIPGVFVTG
GSANPVRSFGCAVGSRDFPGYHWIYWVGPLLGAALAAGYFRLVKMMHYEEANPGQDSPVDV*
>Fusgraminearum_XP011325579.1
MSSSILNNRSARSTPAGANPAFNPPAEASSSSTQSGVPYIRENSPEAESIPSNPAMTPIITHGDSLASPGGRNSE
ADTVVPLQPAVSQDAIRSRPGSRTGNFVTPKAARFSQDGAEPIMQYSSGSVKSARRRTREREDYDFDQAADYAPM
SEYERYYRNEGRNDRDRYTRRGPYPPPTFMNRRRAPPLDSDEEFYSSDDPRIPPNDRRRMMDEEAGYPGRPHAFR
RTSTLNGFNITTGKLQWNELSRQEKSEIMRLPLTQWMNSNFKNHFVAGVGEFIGTTMFLFFAFAGTEVANIQADT
TNRTTTGESTGSLNVSKLLYISIIFGFSLMVNVWVFFRISGGLFNPAVTMAMLMVKAISVTRAIVLFLAQILGSM
LASVVVRYLFPETFNVRTTLGGGASLVQGVFIEALLTAELVFTIFMLAKEKHRATFIAPVGIGLALFIAEMVGVQ
FTGGSLNPARSFGPCVITGSFDTEHWIYWVGPAIGSLIAVCFYWFIKTLEYEMANPGADGDDLNDPTKNPEKRAE
IQASKPVPTAAFGSGKTASILS*

>Fuspseudograminearum_EKJ77666
MGITMRQANSHEARDGEIESRPSFEPTGPRFQSNDLPFAGRLGANQAYVVDGTTSEDEKLLEHQPDATPHMSFRE
LVDVRPITNIDLWKAAMIEGIGTLLSVFVTIWASSSPDVIPAQPTRQLGNFDNAAFIGPLVGGIINVILITLFIT
CFGAVSGAHFNPLITFATFCARLCSLPRMILYIPAQVGGAALAGLLVRASWGGRDFKTGGCWLFTEVVPAREAFV
IELVFSTVLLFLAFGVGLDPRQAKIIGPALGPFLVGLSVGTMSFGSAFTRYGYGGAGMNPARCMGAFVGSRFPSW
HWIHWVANFIACIAHGICYYFIPPWTQVKG*
>Fuspseudograminearum_XP_009259022.1
MPISTINDSISESSVHKSSIPTKIEMSQNEKYSEAPSEAPTIPPPPEQYAWSRIRENCQDAFSEFFGTFVLLLFG
DGVVAQVVLSRGTKGDYQSISWGWGLGVMLGVYVGGKSGGHLNPAVTLANCLFRGHPWRKFPVYAIAQVLGAMAA
AAVVYGNYKSAIDAYEGGPGIRTVIGENATAGVFCTYPAEFMTRTGMFFSEFIASTILQFVIFAMADSANIGAGP
LMPLGLFFLIFGIGACFGWETGYAINLARDFGPRLVSYMLGYGSEVWSAGGYYFWIPMVAPFFGCAFGGFLYDVF
IYTGPSPINTPGMGFGRLVSPRRSTWSNTYNANSPV*
>Fuspseudograminearum_XP009261345.1
MVQFGSRTDASMTGLPTEQAVADRRVGNPKRDRIRNAIVIILGEFCGTFMFLLLSFIGAQTALVTNSPSDPGSAL
LPFSLMYIAASFGTALAVNVWIFYRVSGGMFNPAVTLGLVLVGAVTPIHALLIIPTQLVAAITAAGITDALLPGK
LLVTNALGNGTSVAQGVFIEMFLTAQLVLTVYFLAVEKHRSTHLAPIGIGISVFIAHICATNWTGTSINPARSFG
PSVVAGFHGYDWIYYIGPFMGSLLAFGCYKIFKVLEYQTANPGQDDDDLDKSGHHHFFGHGKEPMPHTHTDTIEP
KDHGVPQRNDSVIDDQMV*
>Fuspseudograminearum_XP_009253247.1
MADTYGMNGHNGHAKHRRSSSMNGRNRLYAQQEPQRTSHLSEFGKHMVAASGEFVGTFLFLYFGYAGNIVAVLQE
PISGPNGTLANNTVMYVAMAYGFSLLVNVWTFYRISGGLFNPAVTFGLCLSGQLPWIRALFLFPSQIIAAMCAGG
LVNAMFPGSASIANTTLGPNTSIAQGVFLEMFFTAQLVFVVLMLAAEKSRDTFLAPVGIGLALFVALIPGVFVTG
GSANPVRSFGCAVGSRDFPGYHWIYWVGPLLGAALAAGYFRLVKMMHYEEANPGQDSPIDV*
>Fuspseudograminearum_XP_009260256.1
MSSSILNNRSARSTPAGANPAFNPPAEASSSSTQSGVPYIRENSPEAESIPSNPAMTPIITHGDSLASPGGRNSE
ADTVVPLQPAVSQDAIRSRPGSRTGNFVTPKAARFSQDGAEPIMQYSSGSVKSARRRTREREDYDFDQAADYAPM
SEYERYYRNEGRNDRDRYTRRGPYPPPTFMNRRRAPPLDSDEEFYSSDDPRIPPNDRRRMMDEEAGYPGRPHAFR
RTSTLNGFNITTGKLQWNELSRQEKSEIMRLPLTQWMNSNFKNHFVAGVGEFIGTTMFLFFAFAGTEVANIQADT
NNRTTTGESTGSLNVSKLLYISIIFGFSLMVNVWVFFRISGGLFNPAVTMAMLMVKAISVTRAIVLFLAQILGSM
LASVVVRYLFPETFNVRTTLGGGASLVQGVFIEALLTAELVFTIFMLAKEKHRATFIAPVGIGLALFIAEMVGVQ
FTGGSLNPARSFGPCVITGSFDTEHWIYWVGPAIGSLIAVCFYWFIKTLEYEMANPGADGDDLNDPTKNPEKRAE
IQASKPVPTAAFGSGKTASILS*
>Fuspseudograminearum_JTGC01000315.1
MAGTQDQSHDYFSKPTTPSTPGQAHLGNVANRIPSTIPDRESGTERSKSTHSRHRVLHGLPSTFMSYMSRRPVAS
SRGFSRMSSEGTASRPTAPQHSSQFHHYAPAEDGYYQENPWFGEADKKPIFSLGKPLPHKVRRKALKPVRPDGKV
DEEMAIVKEEITNEPHPGYASRTTSGQPYRVETQSSLPSRDIQRQQTRTTAAGVAHNDRRNDAGQPVFEYIPGEA
TPTPGHRDPASRVQSKQDNGHSPPDFKVDGEPLGHQEKPCVESGDTDADEMRNWWARLRARHPEPLAEFLATAVA
IFLGLTGTLSVNLSAKQSQPYGTYETSCWAWGFAWMFGIYLGGGVSGAHMNPAISVSLSIFRGFPWKQCAIYVFV
QFIASIVAGALAYAIYADSINYVDPDMTKMSMTFFSTPREWVTLKSAFFNQVVGSAIMMIAVFALGDDQNNPPGA

GMHALVLGFLVTTLKFTLGYNIGSALNPASDFGPRVIAYAVGFRGDNVFHSGWWFYGPWAATLIGSLLGCTLYDG
FVFVGSESPVNFRVDKRVKKLFN*

>Fusoxysporum_5361
MTATNRRKARDSGDVEARPSSEPSGPHYQSYSQPFAGRLGANQAYVVEGGTSEDDHLLHHAPDATPHMSFWELMD
VRPIKNLDLWKAALIEGIGTLLFVYITIWVNISPDIAPAAPTQRFGSFDNAAFLGPLVGGMTNLIFITLFITSFG
AISGAHFNPLITFATFCARLCSLPRLILYVAAQIGGAALAGLLVRASWGGRDFKVGGCWLFTDIVPPKEIFVVEL
VSATLLLFLAFGVGLDPRQAKIIGPALGPFMVGLSVGTMSFASAFARYGYGGAGLNPARCMGAFVGSRFPSWHWI
HWVADGIACIIHGVCYYFIPPWTEVRQ*
>Fusoxysporum_3231
MNDSISESSVHKSSIPTKVEMSQNEKYSEAPSEPPTIPPPPEQYAWSRVREYCQDAFSEFFGTFILLLFGDGVVA
QVVLSRGTKGDYQSISWGWGLGVMLGVYVGGKSGGHLNPAVTLANCIFRGHPWRKFPVYAVAQVLGAMCAAAVVY
GNYKSAFDAYEGGPGIRTVIGENATAGVFCTYPAEFMTRTGMFFSEFIASTILQFVIFAMADSANIGAGPLMPLG
LFFLIFGIGACFGWETGYAINLARDFGPRLVSYMIGYGSEVWSAGGYYFWIPMVAPFMGCAFGGLLYDVFIYTGP
SPINTPGMGLPRLLSPRRSTWSNTYSASSPV*
>Fusoxysporum_1148
MVQFTRADTGMSGLPTEEAVADRRAGSPIPNRVRNAIVIVLGEFCGTFMFLLLSFIGAQTALVTNNPTNSTAPLE
PFSLMYIAASFGTALAVNVWIFYRVSGGMFNPAVTLGLVLVGAVPPLHALAIIPTQLVAAIAAAGVTDGLIPGPL
LVTNSLGNGTSIAQGVFLEMFLTAQLVLTVYFLAVEKHRSTHLAPVGIGISVFIAHICLTNWTGTSINPARSLGP
SVIAGFHGYDWIYYLGPFMGSFLAFGCYKIFKVLEYQTANPGQDDDDLERGSKHHFFGHHEKEPISHSQTDTS
>Fusoxysporum_2910
MASAAEVHGANGFNGHHHHHKQQRTHLSEFGTHMVAASGEFVGTFFFLYFGYAGNIIAVLQEPATGPNGTLASNT
IIWIAMAYGFSLLVNVWAFYRISGGLFNPAVTFGLCLAGQLPWMRALYLFPAQLIASMCAGGLVEAMFPGSASQA
NTTLGPNTSIAQGVFLEMFFTAQLVFVVLMLAAEKSRDTFLAPIGIGLSVFVALIPGVFVTGGSLNPVRSFGCAV
GGRDFPGYHWLYWVGPLLGGALAAGYFRLVKVMHYEEANPGQDSPVDV*
>Fusoxysporum_5739
MRDNSPEADSLPSNPALTPVITHGDNPSASNRNSEADTVVPLQPVISQDPSRVRPGSRTGNFVTPKTARFSQDGG
EPLQYSGGSMKSSRRRYRGEDYDFDQAADYPTVSDYERYWRNEGRNDRYTARRGPYPPPTFMNRRRAHPHDSDEE
FYSSDDPRMPPTGGRRMMDEEMGYPHRPFHHRRASTLNGFNISTGKLEWSNLSPKEKSEIMRLPLTQWMNSNFKN
HFVASLGELIGTTMFLFFAFAGTEVANIQAQTDSKTTTGESTGSLNVSKLLYISIIFGFSLMVNVWVFFRISGGL
FNPAVTIAMLMVKAISMTRAICLFLSQILGSMLASVMVRYLFPETFNVRTTLGGGASLVQGVFIEALLTAELVFT
IFMLAKEKHRATFIAPVGIGLALFIAEMVGVQFTGGSLNPARSFGPCVITGTFDSEHWIYWVGPGIGSLIAVCFY
WFIKTLEYEMANPGADGDDLNDPTKNPEKRAEIQSNAAPSIAFGGGKTPSIRS*
>Fusoxysporum_12344
MTGHQDQNQNHDYFSKPTTPSTPGQVHLGNVASRIPSTISDRETGEPDRAKSTHSRRRGIHGLPSSFVSRMSKRP
ISSRRFTRMSSGNSTARLAAQQHASQYHTYAPAEEGYYEENPWFGEAGKKPIFSLGKPLPHRVRRKVVKPIKPDG
KVDEEMAIVKEESEDTPAGYASRTTSGQPYRTHTQTSITSREMQNQQSRTTAAGVAHNEKRNDAGQPVFDYVPGE
ATPAAIPSYRDPASRVASHQQSNNQPDYKIDGEPLGQQEKPEVEAGETDPNEMRNWWARIRAKHPEPLAEFLATS
VAIFLGLTGTLSVNLSAKQSQSYGTYETSCWAWGFAWMFGIYLGGGVSGAHMNPAISISLSLFRGFPWRQCFIYI
LAQFIASIVAGALAYGIYADSIHYVDPEMDGMSKTFFSTPREWVSLQSAFFNQVVGSAIMMIAVFALGDDQNNPP
GAGMHALVLGFLVTTLKFTLGYNIGSALNPASDFGPRVVAYAVGYRGSN*

>FusLycopersici_AAXH01000716
MTATNRRKARDSGDVEARPSSEPSGPHYQSYSQPFAGRLGANQAYVVEGGTSEDDHLLHHAPDATPHMSFWELMD
VRPIKNLDLWKAALIEGIGTLLFVYITIWVNISPDIAPAAPTQRFGSFDNAAFLGPLVGGMTNLIFITLFITSFG
AISGAHFNPLITFATFCARLCSLPRLILYVAAQIGGAALAGLLVRASWGGRDFKVGGCWLFTDIVPPKEIFVVEL
VSATLLLFLAFGVGLDPRQAKIIGPALGPFMVGLSVGTMSFASAFARYGYGGAGLNPARCMGAFVGSRFPSWHWI
HWVADGIACIIHGVCYYFIPPWTEVRQ*
>Fuslycopersici_EXL45426.1
MLDDANPLSVTCLSCIRQTSPLSISRSSSQAHSRMPIPTMNDSISESSVHKSSIPTKVEMSQNEKYSEAPSEPPT
IPPPPEQYAWSRVREYCQDAFSEFFGTFILLLFGDGVVAQVVLSRGTKGDYQSISWGWGLGVMLGVYVGGKSGGH
LNPAVTLANCIFRGHPWRKFPVYAVAQVLGAMCAAAVVYGNYKSAFDAYEGGPGIRTVIGENATAGVFCTYPAEF
MTRTGMFFSEFIASTILQFVIFAMADSANIGAGPLMPLGLFFLIFGIGACFGWETGYAINLARDFGPRLVSYMIG
YGSEVWSAGGYYFWVSPTTIPRLSISTDSEIQIPMVAPFMGCAFGGLLYDVFIYTGPSPINTPGMGLPRLLSPRR
STWSNTYSASSPV*
>Fuslycopersici_AGBH01000103.1
MVQFTRADTGMSGLPTEEAVADRRAGSPIPNRVRNAIVIVLGEFCGTFMFLLLSFIGAQTALVTNNPTNSTAPLE
PFSLMYIAASFGTALAVNVWIFYRVSGGMFNPAVTLGLVLVGAVPPLHALAIIPTQLVAAIAAAGVTDGLIPGPL
LVTNSLGNGTSIAQGVFLEMFLTAQLVLTVYFLAVEKHRSTHLAPVGIGISVFIAHICLTNWTGTSINPARSLGP
SVIAGFHGYDWIYYLGPFMGSFLAFGCYKIFKVLEYQTANPGQDDDDLERGSKHHFFGHHEKEPISHSQTDTLEP
KDHGVAPRNDSVIDGQMSNA*
>Fuslycopersici_EWZ86261.1

MASAAEVHGANGFNGHPHHHKQQRTHLSEFGTHMVAASGEFVGTFFFLYFGYAGNIIAVLQEPATGPNGTLANNT
IIWIAMAYGFSLLVNVWAFYRISGGLFNPAVTFGLCLAGQLPWMRALYLFPAQLIASMCAGGLVEAMFPGSASQA
NTTLGPNTSIAQGVFLEMFFTAQLVFVVLMLAAEKSRDTFLAPIGIGLSVFVALIPGVFVTGGSLNPVRSFGCAV
GGRDFPGYHWLYWVGPLLGGALAAGYFRLVKVMHYEEANPGQDSPVDV*
>Fuslycopersici_AAXH01000387.1
MGPTAEKNGHDDLGHPRGHLSTFNAHLVAASGEFVGTFFFLYFGYAGNMMAVLQSPYPAINGGLASTTDTWIAIS
YGFSLLVNVWAFYRISGGLFNPAVTLGLCIAGQLPWLRAAFLVPAQLLGSMCAGGLVDAMFPGSVAQANTVLGPY
TSIAKGVFLEMFFTAQLIFVVLMLAAEKSRDTFIAPIGIGLALFVALIPGVFVTGGSLNPVRSFGCAVGGRDFPG
YHWLYWVGPLLGGALAAGYFRLVKMMHYEEANPGQDSPVDV*
>Fuslycopersici_AAXH01000729.1
MSLDPLPTIETTLTSSSPSDMSSSIQSRSARSTPAINASAFNPPEANGSSNERVPFMRDNSPEADSLPSNPALTP
VITHGDNPSASNRNSEADTVVPLQPVISQDPSRVRPGSRTGNFVTPKTARFSQDGGEPLQYSGGSMKSSRRRYRG
EDYDFDQAADYPTVSDYERYWRNEGRNDRYTARRGPYPPPTFMNRRRAHPHDSDEEFYSSDDPRMPPTGGRRMMD
EEMGYPHRPFHHRRASTLNGFNISTGKLEWSNLSPKEKSEIMRLPLTQWMNSNFKNHFVASLGELIGTTMFLFFA
FAGTEVANIQAQTDSKTTTGESTGSLNVSKLLYISIIFGFSLMVNVWVFFRISGGLFNPAVTIAMLMVKAISMTR
AICLFLSQILGSMLASVMVRYLFPETFNVRTTLGGGASLVQGVFIEALLTAELVFTIFMLAKEKHRATFIAPVGI
GLALFIAEMVGVQFTGGSLNPARSFGPCVITGTFDSEHWIYWVGPGIGSLIAVCFYWFIKTLEYEMANPGADGDD
LNDPTKNPEKRAEIQSNAAPSIAFGGGKTPSIRS*
>Fuslycopersici_MALP01000202.1
MTGHQDQNHDYFSKPTTPSTPGQVHLGNVASRIPSTISDRETGEPDRAKSTHSRRRGLHGLPSSFVSRMSKRPIS
SRRFTRMSSGNSTARPTAEQHASQYHTYAPAEEGYYEENPWFGEAGKKPIFSLGKPLPHRVRRKVVKPIKPDGKV
DEEMAIVKEESEDTPAGYASRTTSGQPYRTHTQTSIASREMQNQQSRTTEAGVAHNEKRNDAGQPVFDYVPGEAT
PAATPSYRDPASRVASHQQSNNQPDYKIDGEPLGQQEKPEVEAGETDPNEMRNWWARIRAKHPEPLAEFLATSVA
IFLGLTGTLSVNLSAKQSQSYGTYETSCWAWGFAWMFGIYLGGGVSGAHMNPAISISLSLFRGFPWRQCFIYILA
QFIASIVAGALAYGIYADSIHYVDPEMDGMSKTFFSTPREWVSLQSAFFNQVVGSAIMMIAVFALGDDQNNPPGA
GMHALVLGFLVTTLKFTLGYNIGSALNPASDFGPRVVAYAVGYRGSNVFHSGWWFYGPWAATLIGSVVGCALYDG
FVFVGSESPINFRFSKEIKNRAN

>Fusvasinfectum_AGNC01000296.1
MTVANRRKARDSGDVEARPSSEPSGPHYQSYSQPFAGRLGANQAYVVEGGTSEDDHLLHHAPDATPHMSFWELMD
MRPIKNLDLWKAALIEGIGTLLFVYITIWVNISPDIAPAAPTQRFGSFDNAAFLGPLIGGMTNLIFITLFITSFG
AISGAHFNPLITFATFCARLCSLPRLILYVAAQIGGGALAGLLVRASWGGRDFKVGGCWLFTDIVPPKEIFVVEL
VSATLLLFLAFGVGLDPRQAKIIGPALGPFMVGLSVGTMSFASAFARYGYGGAGLNPARCMGAFVGSRFPSWHWI
HWVADGIACIIHGVCYYFIPPWTEVRQ*
>Fusvasinfectum_EXM16766.1
MLDDANPLSATCLSCIRQTSPLSVSRSSSQAHSRMPIPTMNDSISESSVHKSSIPTKVEMSQNEKYSEAPSEPPT
IPPPPEQYAWSRVREYCQDAFSEFFGTFILLLFGDGVVAQVVLSRGTKGDYQSISWGWGLGVMLGVYVGGKSGGH
LNPAVTLANCIFRGHPWRKFPVYAVAQVLGAMCAAAVVYGNYKSAFDAYEGGPGIRTVIGENATAGVFCTYPAEF
MTRTGMFFSEFIASTILQFVIFAMADSANIGAGPLMPLGLFFLIFGIGACFGWETGYAINLARDFGPRLVSYMIG
YGSEVWSAGGYYFWIPMVAPFMGCAFGGLLYDVFIYTGPSPINTPGMGLPRLLSPRRSTWSNTYSASSPV*
>Fusvasinfectum_AGNC01000077.1
MVQFTRADSGMSGLPTEEAVADRRAGSPIPNRVRNAIVIVLGEFCGTFMFLLLSFIGAQTALVTNNPTNSTAPLE
PFSLMYIAASFGTALAVNVWIFYRVSGGMFNPAVTLGLVLVGAVPPLHALAIIPTQLVAAIAAAGVTDGLIPGPL
LVTNSLGNGTSIVQGLFLEMFLTAQLVLTVYFLAVEKHRSTHLAPVGIGISVFIAHICLTNWTGTSINPARSLGP
SVIAGFHGYDWIYYLGPFMGSFLAFGCYKIFKVLEYQTANPGQDDDDLERGRKHHLFGHHEKEPISHSQTDTLEP
KDHGVAPRNDSVIDGQMSNA*
>Fusvasinfectum_AGNC01000348.1
MASAAEVHGANGFNGHHHHHKQQRTHLSEFGTHMVAASGEFVGTFFFLYFGYAGNIIAVLQEPATGPNGTLANNT
IIWIAMAYGFSLLVNVWAFYRISGGLFNPAVTFGLCLAGQLPWMRALYLFPAQLIASMCAGGLVEAMFPGSASQA
NTTLGPNTSIAQGVFLEMFFTAQLVFVVLMLAAEKSRDTFLAPIGIGLSVFVALIPGVFVTGGSLNPVRSFGCAV
GGRDFPGYHWLYWVGPLLGGALAAGYFRLVKMMHYEEANPGQDSPVDV*
>Fusvasinfectum_EXM26604.1
MSLDPLPTIETTLTSSSPSDMSSSIQSRSARSTPAINASAFNPPEANGSSNERVPFMRDNSPEADSLTSNPALTP
VITHGDNPSASNRNSEADTVVPLQPVISQDPSRVRPGSRTGNFVTPKTARFSQDGGEPLQYSGGSMKSSRRRYRG
EDYDFDQAADYPTVSDYERYWRNEGRNDRYTARRGPYPPPTFMNRRRAHPHDSDEEFYSSDDPRMPPTGGRRMMD
EEMGYPHRPFHHRRASTLNGFNISTGKLEWSNLSPKEKSEIMRLPLTQWMNSNFKNHFVASLGELIGTTMFLFFA
FAGTEVANIQAQTDSKTTTGESTGSLNVSKLLYISIIFGFSLMVNVWVFFRISGGLFNPAVTIAMLMVKAISMTR
AICLFLSQILGSMLASVMVRYLFPETFNVRTTLGGGASLVQGVFIEALLTAELVFTIFMLAKEKHRATFIAPVGI
GLALFIAEMVGVQFTGGSLNPARSFGPCVITGTFDSEHWIYWVGPGIGSLIAVCFYWFIKTLEYEMANPGADGDD
LNDPTKNPEKRAEIQSNAAPSIAFGGGKTPSIRS*
>FusVasinfectum_AGNC01000196.1
MTGHQDQNHDYFSKPTTPSTPGQVHLGNVASRIPSTISDRETGEPDRAKSTHSRRRGLHGLPSSFVSRMSKRPIS
SRRFTRMSSGNSTARPTAQQHASQYHTYAPAEEGYYEENPWFGEAGKKPIFSLGKPLPHRVRRKVVKPIKPDGKI

DEEMAIVKEESEDTPAGYASRTTSGQPYRMHTQTSIASREMQNQQSRTTAAGVAHNEKRNDAGQPVFDYVPGEAT
PAATPSYRDPASRVASHQQSNNQPDYKIDGEPLGQQEKPEVEAGETDPNEMRNWWARIRAKHPEPLAEFLATSVA
IFLGLTGTLSVNLSAKQSQSYGTYETSCWAWGFAWMFGIYLGGGVSGAHMNPAISISLSLFRGFPWRQCFIYILA
QFIASIVAGALAYGIYADSIHYVDPEMDGMSKTFFSTPREWVSLQSAFFNQVVGSAIMMIAVFALGDDQNNPPGA
GMHALVLGFLVTTLKFTLGYNIGSALNPASDFGPRVVAYAVGYRGSNVFHSGWWFYGPWAATLIGSVVGCALYDG
FVFVGSESPINFRFSKEIKNRAN*

>Fusverticillioides_AAIM02000133.1
MAVTNRRNARDSSDVEARPSLEPSGPRYRSNSQPFAGRLGANQAYVVEGGTSKDDHLLHHAPDATPHMSFRELMD
MRPIKNLDLWKAALIEGIGTLLFVYITIWVNISPDTAPAAPTQRFGSFDNAAFLGPLIGGITNLIFITLFVTSFG
AISGAHFNPLITFATFCARLCSLPRLILYVAAQIGGGALAGLLVRASWGGRDFKVGGCWLFTDIVPPREIFVVEL
VSATLLLFLAFGVGLDPRQAKIIGPALGPFMVGLSVGTMSFASAFARYGYGGAGLNPARCMGAFVGSRFPGWHWI
HWVADGTACIIHGVCYYFIPPWTEVRQ*
>Fusverticillioides_EWG48857.1
MNDSISESSVHKSSIPTKVEMSQNEKYSEAPSEPPTIPPPPEQYAWSRVREYCQDAFSEFFGTFILLLFGDGVVA
QVVLSRGTKGDYQSISWGWGLGVMLGVYVGGKSGGHLNPAVTLANCIFRGHPWRKFPVYAVAQVLGAMCAAAVVY
GNYKSAFDAYEGGPGIRTVIGENATAGVFCTYPAEFMTRTGMFFSEFIASTILQFVIFAMADSANIGAGPLMPLG
LFFLIFGIGACFGWETGYAINLARDFGPRLVSYMIGYGSEVWSAGGYYFWIPMVAPFLGCAFGGLLYDVFIYTGP
SPINTPGMGLPRLLSPRRSTWSNTYSASSPV*
>Fusverticillioides_EWG36264.1
MVQFTRANSGMSGLPTEEAVADRRAGSPIPNRVRNAIVIVLGEFCGTFMFLLLSFIGAQTALVTNNPTNSTAPLE
PFSLMYVAASFGTALAVNVWIFYRVSGGMFNPAVTLGLVLVGAVPPLHALAIIPTQLVAAIAAAGVTDGLIPGPL
LVTNALGNGTSIAQGVFMEMFLTAQLVLTVYFLAVEKHRSTHLAPIGIGISVFIAHICLTNWTGTSINPARSFGP
SVVAGFHGYDWIYYLGPFMGSFLAFGCYKIFKVLEYQTANPGQDDDDLERGSKHHFFEHHEKEPIAHSQTDTLEP
KDHGAAPRNDSPIDGQMSHA
>Fusverticillioides_EWG49201.1
MANAAEVQAANGFNGHHHHHKQQKTHLSEFGTHMVAASGEFVGTFFFLYFGYAGNIIAVLQEPATGPNGTLANNT
IIWIAMAYGFSLLVNVWAFYRISGGLFNPAVTFGLCLAGQLPWMRALYLFPAQLIASMCAGGLVEAMFPGSASQA
NTTLGPNTSIAQGVFLEMFFTAQLVFVVLMLAAEKSRDTFLAPIGIGLSVFVALIPGVFVTGGSLNPVRSFGCAV
GGRDFPGYHWLYWVGPLLGGALAAGYFRLVKMMHYEEANPGQDSPVDV*
>Fusverticillioides_EWG52904.1
MSLDPLPTIETTLTSSSPSDMSSSIQSRSARSTPAINASAFNPPEANGSSNERVPFMRDNSPEADSLPSNPALTP
VITHGDNPPASNRNSEADTVVPLQPAISQDPSRVRPGSRTGNFVTPKTARFSQDGGEPLQYSGGSMKSSRRRYRG
EDFDFDQAADYPTVSDYERYWRNEGRNDRYTTRRGPYPPPTFMNRRRAHPHDSDEEFYSSDDPRMPPTGGRRMMD
EEMGYPHRPFHHRRASTLNGFNISTGKLEWSDLSPKEKSEIMRLPLTQWMNSNFKNHFVASLGELIGTTMFLFFA
FAGTEVANIQAQTDSKTTTGESTGSLNVSKLLYISIIFGFSLMVNVWVFFRISGGLFNPAVTIAMLMVKAISMTR
AICLFLSQILGSMLASVMVRYLFPETFNVRTTLGGGASLVQGVFIEALLTAELVFTIFMLAKEKHRATFIAPVGI
GLALFIAEMVGVQFTGGSLNPARSFGPCVITGTFDSEHWIYWVGPGIGSLIAVCFYWFIKTLEYEMANPGADGDD
LNDPTKNPEKRAEIQSNAAPSIAFGGGKTPSIRS*
>Fusverticillioides_AAIM02000170.1
MTGHQEQNHDYFSKPATPSTPGQVHLGNVASRIPSTISDREAQPDRAKSTHSRRRALHGLPSSFVSRMSKRPISS
RRFTRMSSGHSTARPTVQHHASQYHEYAPAEEGYYEENPWFGEAGKKPIFSLGKPLPHRVRRKVVKPIRPDGKVD
EEMAIVKEDSEDTPAGYASRTTSGQPYRMHTQTSIASRDVQNQQSRTTASGVAHNVKRNDAGQPVFDYVPGEATP
AATPSHRDPASRVASHQQSNNQPDYKIDSEPLGQQEKPEVEAGETDPNEMRNWWARIRAKHPEPLAEFLATSVAI
FLGLTGTLSVNLSAKQSQPYGTYETSCWAWGFAWMFGIYLGGGVSGAHMNPAISISLSLFRGFPWRQCFIYILAQ
FIASIVAGALAYGIYADSIHYVDPEMDGMSKTFFSTPREWVSLQSAFFNQVVGSAIMMIAVFALGDDQNNPPGAG
MHALVLGFLVTTLKFTLGYNIGSALNPASDFGPRVVAYAVGYRESNVFHSGWWFYGPWAATLIGSVVGCALYDGF
VFVGSESPINFRFSKEIKHRAN*

>Fusvirguliforme_AEYB01001170.1
MAGPVSGDVESRPPVELGSPPRYHSSSHPFAGRLGANQAFTVDRRTSEDEKLLEREPDATPHMPFRELLDCRPIL
SRYLWKAALIEGIGTLMQVYISIWISISPPNLPTRPTAQLGNFDNAAFIGPLVGGITNIIFISLFISSFGAGAHF
NPLITFATFCARLCSLPRLILYVGAQISGGVLAGLLVRASYGTRDFKVGGCWLDPDIVPVREIFVVELVSATILL
FLAFGLGLDPRQAKIVGPTLAPFLVGLASGTLTFSTAFTRYGYGGAGLNPARCMGAFVGTRFPTWHWIHWVGDGI
ACIIHGAVYYFVPPWTKETD*
>Fusvirguliforme_AEYB01000058.1
MARFASFQSNYSTQHDGPPHPGEVGHPLPSTARNSAVIVLGEFCGTFMFLLLSYIGAQTALVTNNPSDPSAPLEP
FSLMYIAASFGTALAVNVWIFYRVSGGMFNPAVVTVGLMLVGAVKPLNGLLIIPTQLVAAIAAAAVTDGLIPGPL
LVTNTLGNGTSKAQGVFIEMFLTAQLVLTVYFLAVEKHRSTHLAPIGIGISVFISHICATNWTGTSINPARSFGP
AVVTMFVGYHWIYWVGPFMGALLAFGCYKVFKWLEYQTANPDQDDDDLEKGRRHHFGLHGHAQEKAVMPREQVET
IPPKDASPHQRNDSMIDGQMSPISP*
>Fusvirguliforme_AEYB01001186.1

MGPSADTNGHDGPGHRRRHLSTVKAHLVAASGEFVGTFFFLYFGYAGNMMAVLQSPYPAVNGGLASTTDIWIAMS
YGFALLVNVWAFYRISGGLFNPAVTLGLCIGGQLPWLRAAFLVPAQLLGSMCAGGLVDAMFPGSVAQANTVLGPY
TSIAKGVFLEMFFTAQLVFVVLMLAAEKSRDTFIAPIGIGLALFVALIPGVFVTGGSANPVRSFGCAVAGTDFPG
YHWIYWVGPALGAALAAGYYRLAKRMHYEEANPGQDAPHDV*
>Fusvirguliforme_AEYB01000674.1
MATSDEAGVLDRPPHQRYQLSPVGRHLVAASGEFVGTFFFLYFGYAGNLMAALQAPYTAPDGGIASSTDIWIAMS
YGFSLLVNAWAFYRISGGLFNPAVSLGLCVGGQLSWTRAAFLFPAQLLGSLCAGGLVDAMFPGQVEQANTLLGPN
TSIARGVFLEMFFTAQLVFVVLMLAAEKSRDTFLAPVGIGLALFVALIPGVSVTGGSANPVRSFGCAAAGASFPG
YHWIYWVGPALGATLAALYYRLVKRMHYEEANPQDSPHEV*
>Fusvirguliforme_AEYB01000247.1
MPLSSLPSIETSLRSPTPDFDMSSLHTPSVTRSARSTPAANVSAFSPPGEAISSPNNESVPFAPPASPTDHSPPR
NSETDTIVPLQPAASHEAIRSRPGSRTGAFATPKGATRFTHDVEPLPLQHSGGSMKSQRRRFREDWDYDQEPRDH
PTLGDYDRYWRNEGRPERYTSRRSNGPPPAFMNRRRAAAHDSDEDMYNDDLRYTKDPFESSRGRRLTDEEMGFHQ
AGHHHQRRPSSSHGVNLASGRLDWNNLTAHEKAQVMRLPLTQWMNSDFKNHFVASLGEFIGTTMFLFFAFAGTEV
ANIQSNTSSKTTTGESTGFSVSTLLYISIIFGFSLMVNVWVFFRISGGLFNPAVTIAMLMVKAISLTRAVCLFVS
QILGAMLASVVVLYLFPEAFNVRTTLGGGASLVQGVFIEAILTAELVFTIFMLAKEKHRATFIAPVGIGLALFIA
EMVGVQFTGGSLNPARSFGPCVVTSTFDTEHWIYCVGPFVGSLIAVGFYWFIKTLEYEMANPGADGDDANDPTKN
PEKRAEIQASKPPSMQFGIGKSPSNRS*

>Lacbi2_317173
MSGQHQITEQSSRNPLSRVSTLLPEKPLSPTSTYAGTQKHPEAPRQSSFLIQLQNIRNAIRKPMAEFFGVALLII
FGAGSACQVVLSTNPDVASSARGSFLSINFGWAIGIAMGVWVSGGISGGHINPAITIAMATYRGFPWRKVPSYIL
AQVLGGVVGAGLVYANYIHAIDIFEGGHHIRTQATASLFATYALPYMTQASCFFSEFLATAVLSMMVFALTDKRN
HSPTNGLLPFALFILFVGLGASLGMETAYALNPARDFGPRLFLAMAGYGKALFNYRSQYWLWAPIIAPVLGAQAG
GLLYDTFLNDGDNSPIKWRCASSQEHQLAEVV*
>Lacbi2_443240
MDDKFDDDALPNSKTTPEDYGDKLAEYDYTNTFPNTWMRLREPFREYIAEFVGVAVLIIFGVGADCQVVLSANTG
VAPSPKGDYLSLNCGWAIGTAMGVWISGGISGGHINPAVTLALATWRGFPWRKVPGFLFAQLLGGIVGAGLVYVN
YIHAIDIVEGGRHVRTLDTAGLFATYAADHMTNVSCFFSEFLATAVLIVVIHAMNDKRNAPPPAGLAPLVLFFLI
LGIGASLGMETGYAINPARDLGPRMLTAMVGYGRQVFAFRNQYWIWCPVIAPFLGAQVGTIFYDLFFYKGQDNVF
GRLGSHIHISPA*
>Lacbi2_576801
MFTLAHHRHAIRKPMAEFFGVALLVIFGAGAACQVVLSTNPNSFLSINFGWAIGIAMGAWISGSISGGHINPAIT
IAMATYRGFPWREVPSYILAQVLGGVVGAALVYANYIHAIDVFEGGRHIRTQATASLFATYALPYMTQVSCFFSE
FLATAVLAMMVLALTDNRNGAPTNGLSPFALFVLFIGLGASLGMETAYALNPARDFGPRLFLAMAGYGKALFNYR
SQYWLWAPIIAPVLGAQAGGLLYDTFLYDGDDSPIKWR*
>Lacbi2_568479
MKLTISHHKCAIRKVMAEFVGVALLVIFGAGTACQVVLSTNPSSFLSINFGWAIGIATGAWVSAGISGGHINPAI
TIAMATYRGFPWREVPGYIFAQALGGFVGAALVYANYFHAIDIFEGGHIRTQATASLFATFALPYMTQASCFFSE
FLATAVLFIVFLALNDKHNGALTNGLLPFALFILFIGLGASLGMQTGYAVNPARDFGPRLFLAMAGYGKAVFNYR
RQYWIWAPIIAPILGAQAGGLLYDTSIYNGDDSPIKWR*
>Lacbi2_671860
MSATPIIHLRDVKKRTGVLNAWERVRNKPQVHWAMECFAEALGVFFYVYFGLGSTAAWVIGNILKQSGLSSVFQI
GFAYAFGILFAIGVCAATSGGHFNPCVTIAFTIFRGFPPLKAVRYIVAQILGAYIASALVYNQWKVLIVESELLL
KQAGVYETTMFTPNGPAGIFALYLLPGAQTLPRAFLNEFVNCFVLALVIWAALDPTSFMIPPVMAPFIIAAAYAG
SIWGYAVPAISLNSARDIGCRLFALTIWGKSAAGGSYSAITALVNIPATLLAAVVYELFLVDSDRVVAGSHLEFM
NVAANHRRHRHQAEDDNHGDADDSSQEKPV*
>Lacbi2_482072
MSNAPLVHLSDLQKRLRVFAVWEKVRNDGKVHWAIECFAEMFGVFLYVYFGLGSTAGWVIGNIIKETNLSSILQI
GLAYAFGIWFAIGLCSSSSGGHFNPCVTLSFVVFKGFPKLKACRYIIAQILGAYIASALVYSQWNVLIEECTLGL
IKAKAYDTTMFTPNGPAGIFALYLVPGAQSVPRALLNEFVNSTLIGMIIWAALDPTNMMVPPAMGPLFISLAYAA
VIWGFATPAVALNTARDLGARLFAMSIWGTKAAGSGYSAIACLINIPATLLGVFLYEVFFTDSDRVVSPAALTIM
NAHANHRRLHHGHGEADKRDSTEKPTITTYEHAGGNGVEVSHV*
>Lacbi2_456764
MHPQVASLFDNVYEDLAAATLEFIGTAFFLLFGLGGIQASTAEDTASGQPPASGIEHVLYISTCMGLSLVVSAWL
FFRVTGGLFNPNISFALLLVGGLKPLRFVLFCIAQLTGAIAGAAIVRGLTSAPLSVNNVLQQGTSAAQGVFIEMF
ITAALVLSVLMLAAEKHEATPFAPVGIGLTLFACHLFAVYYTGAAMNSARAFGPAVISGFPEPQHWVYWVGPFLG
SLLGAGFYATLKHYKYWHLNPDQATSDYRKSPSDPVALLKSTAETFINVGDEETRNGCASNEEGVRATGDEKSSN
ATSSRTNFSPV*

>Mycfi2_173306

MSKVTTCSARNDPASFQLHFQGAKVREHPFAGRIGGNQELCLDPDDEAVKKQPDAAPIISLSQSLDPPGFLVFEY
YKMGLIEGPGTCLLVFVSGAGANALTTLGDSVSPMAIALYAALMNWVALTLFVYTTAPASGGHLNPSITLGTLFA
GLSSLPRSLIYVVSQSVGAIVGGFWLRLGLGKSGYFPSGVVPGCTVDQQLVSRGQLFVLEYVFALAQLFLAFGVG
LGPRNAKTYGPAFAPVLVGLTLALGTLASAFVKEGYSGICKHHSRLVCLTCQKADRGDMQYNYLHWFATLAACMV
HGVFYHTVPPYKKSSGKARREALPSFSIMQVKKARFGPSVILGRDGENGRALKHDQVSSRSPDCMDEDLIVEIWE
ASLDMTVSCLRCTFTSMVL*

>Mycfi2_108013
PRRAATVAARRFRSPQRPSTALQPETSNHSSLRRRSTAASKRSQGAEARSSTAAGRSKLQTLAGPEITPQVEATY
QPYVNPGYAELNPEYEQPANAKPVWSLAKPLPRVVRPGMVPTSSEIFQSRQHPQLPGGNTQKLGIEADPNDLEAG
RIQPAINPAKVSAQLKDSRAQREENFLSSQIGRRGTVTGRGRATSRASQYQVSFGEPEKSHTQQGQDGKAGEEVP
LVSPGMERTPSAPDELGATPLEAIPERHEPPTRPASLAEGEDDDASQATLHEDEEPDLWVDMDPIKPIDNPPLVA
EVHNNHTWWSIVRTQHREFLAEFLATFVQLTTGFCADTQTTLNNNGNPNSTAWAWGFSTMIGIYISGGISGAHLN
PAITLMLWFYRGFPKRKVPEYVLAQLLAAFLAALVAYGLYFAGIQNYINTSSTTDPASDILNGFVTSRRFTFIDT
ATAFFNEFLGIAFLGCTILALGDDQNAPPGAGMNSLIIGLVITGLSLSFVYNTGLAMNPTRDLGPRLAMLALGYG
KELFTNPYWFYGPIVAPILGAFAGGALYDIAIFTGGESPINYPWTRTKRSYRKGNAKWKRRLRL*
>Mycfi2_33768
NTWSAIRLHLQRPLAECLGTTIFIFLGIAGNLSVLTSSYQTGTMQSIYWCWGFAVMLAIYIAGGGSGAFLNPALT
IMLTIFRGFPARRVPVYVFVQVLGAFVGGLMAFGVYRDGLVHLGGGGGLISETTGSAFYTEPKEWVSNSTAFFTE
VLGTAVIACSILALGDSSNSPPGAGMHAFIIGLLTTAVTMSLGWSTGGCFNPVRDLGPRLATIAAGYPVSSFSAR
DHWWIWGPWCATITGALVGGLVYDVCIFKGGESPVNYSFGRWKVEGLKREQGLAGMFGMHEKEGEIERKLESGDI
HDDPKLTEM*
>Mycfi2_133800
MTAELKKEANSALTWSRIRRTWREPLSEFMGTFILIMFGDGVVAQVVLSRGTKGDYQSISWGWGIGVMLGVYASG
ISGAHINPAVTFANCVFRKFPWKKFPVYAVAQVLGAMCAAAVVYGNYKSAIDTFEGGAGIRTVPGYSENASAGIF
CTYPAAFMSNTGQFFSEFIASTLLMFLIYAIKDDHNIGAKNLTPLALFFIIFGIGACWGWETGYAINLARDFGPR
LVSYMVGYGPNVWRAGNYYFWVPMVAPFCGCTFGGFLYDVLLFTGQSPINTPYWGFYRFIPSLRRQYKGMRWDEE
NAQEDEDVSVH*
>Mycfi2_99980
MATLDEELDHAGHNHLLWPKVKIALKEPIAEFWGTFILVLFGDAAIAQTMLSGTAAGRASSPGGAGFGAWDTISW
AWGLGLMLGVYVAGDSGAFLNPAICLASCIFRKLPWRRLPMYWLAEFLGAFVAAGVVYGNYVNGINQYEGHGIRS
VASADNPTGTAGIFATFPASDLTKASQFFDQFIGSALLVFLIWTLKDDSNKGKFVASGAWFPLGLFFVMMGIATA
FGWQTGFAINPARDLGPRVMIAAIGYSGVWSAGGYYFWVPIVAPFCGAVVGAFLYDIFIYTGETPVNTPWMGLKK
LLNPKRTIQERLEHQRQQSIV*
>Mycfi2_165933
MSDNSHMERLQTLDLELGATEAVVQHYARHVVATKPVSQRKLNFERSRPRWLRECMAEATGVFFYVFPGIAAVAS
MVLNKANPAYGSFFEVGWAFALGIAFAIITCAPTSGGHFNPAITICFAVWQGFPWKKVPSYIFSQIFGAFIAGLF
LMGLYHQQLSAFQAELRAAGESSVPTMSSILCAYPLPNQTNLGYLFLIEFFVDSYIGIIIWACLDPANPFITGAS
APFVIGLAYAAMVWGFAPITISTNLARDLGTRIVAAIFYGGEAFSYHEYSWIAILVNVPATLFATGYYEFLMRDS
LAKIGKGAARHEHGEEGLGLHLTKSGISRVRTEGNFKPGSSSTSEHEKV*
>Mycfi2_99603
MQQSSRNNGGKGDVASSARSSPIRSHLIACMGEFGGTFMFLFFAFMLHITATAQSDPTKPPDTITVLTIAFAYAF
FYGLWLLINALILQGINGGMFNPAVTLGLTFSGVLSSTRAALLIPIQLLAGVTAAAVVRIVLPIGNMGRVYTSLT
AGTSVAQGLFLEMFLTSLLVIVAIIFVPRTGALSFMAPLGIGLALFVGIFAGLGYTGASLNPVRSFAPCAVTSDF
PRSHWIYWAGPFLGALLASVYSQFARIIRTGKVRPNISQDNLGERYAHEE*
>Mycfi2_134906
FVATLGEFFGTTMFLFFAFAGTQVANIGSSGNQDQTTTNASTGFSPIVLLYISLSFGFSLMVNVWVFFRISGGLF
NPAVTLAMVIVQGVGVVRAVLLVGAQICGAIFSSYIVSVLFPTTFNVRTTLSDGTSVVRGVFIEALLTAELVFTI
YMLANEKHKATFMAPIGIGLALFIAEMVGVYYTGGSLNPARSFGPCVISGVWDSEHWIYWIGPCAGAFIAFGFYR
FIKMLEYEMANPGQESSSKEEAAAEAAEMTPKKESV*
>Mycfi2_120962
MLANQTSTPSWQTNSVLTNFLVQVGEFIGTFLFLFFAFAATQVANAAATGSTNTTSIVQVPNTSALVYISLAFGF
SLAVNAWVFFRISGGLFNPAVTLGMALIGAVTWVRAGLIFVAQILGAMASAGVVSALFPGPLAVSTTLGGGTSIT
RGLFIEMFLTAELVFTIFMLAAEKHKGSFLAPIGIGLSLFIAHLSGVYFTGASLNPARSFGPCVALRSFPGYHWL
YWVGPALGALVAVGFYRFVKILEYETANPGQDFNEHEAA*
>Mycfi2_76398
MEDKLSFGRPMAGIFQPREHAKQSPARNHFVAATGEFVGTFMFLFFAYLGHSMSVATASDTSRIGTNSNSTIIYI
SMSYGLSLLVTAWALYRVSGGLFNPAVTLGLVITGQLPAVRGAIFFPTQIIGGIAAAAVASAIIPGDIAVTQTTL
ANGMNQAQGVFLEMFLTAYLVFVILMLAAEKSKATFIAPIGIGMALFVAQIAGVYYTGASLNPARSFGPCVAAAK
FQGYHWIYWIGPFLGAIIAGGYFHFVKFFNYYEANPGQDSAGGAFADDINVSVSLGR*
>Mycfi2_100823
MSNRSSWLVPPQMDKPGLGYIEPRNAYRQPAWRNHFVACTGEFVGTFLFLLFAFLGHSTAVYQAPATGPNGLHTN
ETLMYIALSYGFSLLVNAWTMYRVSGGLFNPAVTLGLVLGGGLSAVRGLLFFPVQLLAAISAAAVVEALIPGSIR

QVQTTLAPEVNVAQGLFLEMFLTSLLVFTVLMLAAEKWRATFVAPVGIGIALFIAELAGAVYTGASLNPVRSFAP
CVVSPNFQSYHWIYWVGPLLGALLSGSYYHFVKFFNFWQANPGQDS*
