# Supplementary figures and images for: MIP diversity from Trichoderma: Structural considerations and transcriptional modulation during mycoparasitic association with Fusarium solani olive trees
Source: PLoS One. 2018 Mar 15;13(3):e0193760. doi: 10.1371/journal.pone.0193760 (PMC5854309; doi:10.1371/journal.pone.0193760)

## S2 Fig

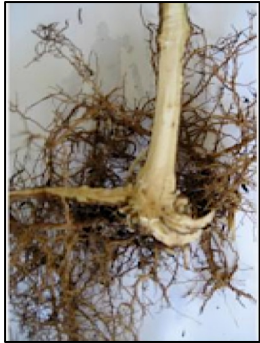

Water  
control

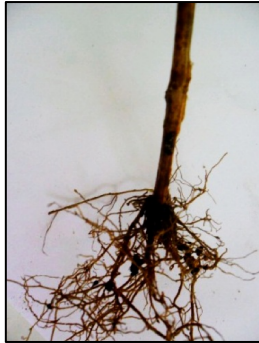

*F. Solani*  
*Fso14*

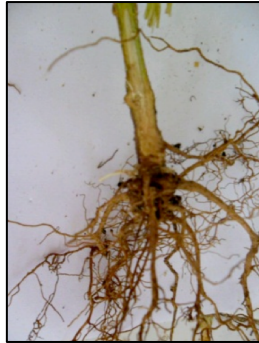

*T. Harzianum*  
*Ths97*

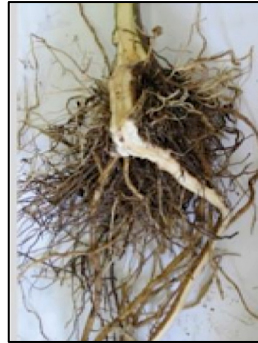

Preventive  
*Ths97 / Fso14*

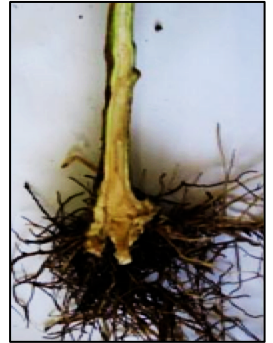

Curative  
*Fso14 / Ths97*

Supplement: S2 Fig — Preventive treatment: Ths97-treated plants subject to Fso14 infestation; Curative treatment: Fso14 infested plants treated with Ths97. Dual inoculation contexts were set up with a 10-day delay between each fungal inoculation. Fungi were inoculated on roots. (PDF) [file pone.0193760.s002.pdf]

S3 Fig.

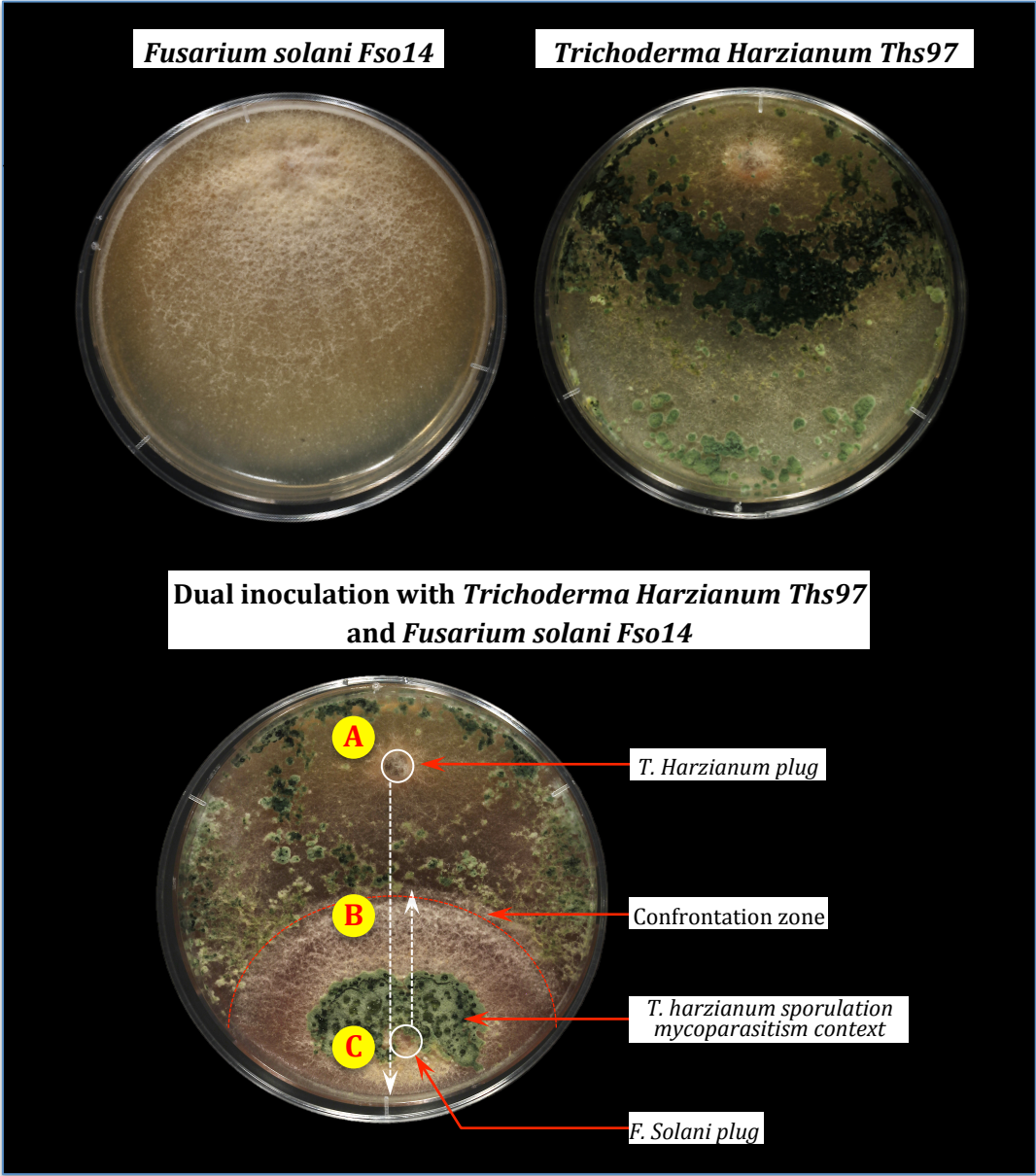

Supplement: S3 Fig — Mycelial were grown in Petri dishes on PDA medium. Slides show 6 days of growth at 27°C. Letters A, B, and C on dual culture assay correspond to area sampled for molecular experiments, with (A) Ths97 individually, (B) area of confrontation between mycelia, and (C) area of overlap of Ths97 on Fso14. (PDF) [file pone.0193760.s003.pdf]
