# Supplementary material for: MIP diversity from Trichoderma: Structural considerations and transcriptional modulation during mycoparasitic association with Fusarium solani olive trees
Source: PLoS One. 2018 Mar 15;13(3):e0193760. doi: 10.1371/journal.pone.0193760 (PMC5854309; doi:10.1371/journal.pone.0193760)
Supplement: S1 Table — Reference species for MIP nomenclature: Mycosphaerella fijiensis (Mycfi) and Laccaria bicolor (Lacbi). AQP, aquaporins; AQGP, aquaglyceroporins; XIP, X-intrinsic proteins. (PDF) [file pone.0193760.s004.pdf]

S1 Table. Features of the non-redundant representative fungal MIP proteins from *Trichoderma* and *Fusarium* species used in the phylogenetic analysis.

Reference species for MIP nomenclature: *Mycosphaerella fijiensis* (Mycfi) and *Laccaria bicolor* (Lacbi).
AQP, aquaporin; AQGP, aquaglyceroporin; XIP, X-intrinsic protein.
NCBI [http://www.ncbi.nlm.nih.gov/] ; JGI [http://genome.jgi-psf. org/]

Species	ID protein [JGI]; [NCBI]*	Nomenclature used in this work	MIP sub-family	Phylogeny group	TMH number	AA number	Molar mass	Pi
			Trichoderma					
Trichoderma asperellum	64497	Triasperellum_64497	XIP	◆	6	301	32,22	8,19
	60030	Triasperellum_60030	Fps-like	●	6	341	32,64	7,62
	79784	Triasperellum_79784	Fps-like	■	6	581	64,26	5,86
	150529	Triasperellum_150529	"Other" AQGP	◆	6	339	36,79	6,19
	142240	Triasperellum_142240	classicAQP	▲	6	311	32,63	7,92
	170459	Triasperellum_170459	classicAQP	■	6	303	32,33	8,77
Trichoderma atroviride	319992	Triatroviride_319992	XIP	◆	6	342	32,1	8,75
	39327	Triatroviride_39327	Fps-like	●	6	299	32,65	7,6
	283564	Triatroviride_283564	Fps-like	■	6	594	65,63	5,88
	90169	Triatroviride_90169	"Other" AQGP	◆	6	344	37,57	6,75
	6990	Triatroviride_31598	classicAQP	●	6	277	30,1	7,7
	43816	Triatroviride_43816	classicAQP	▲	6	313	32,73	7,8
	31598	Triatroviride_6990	classicAQP	■	6	308	32,45	9,24
Trichoderma citrinoviride	1108082	Tricitrinoviride_1108082	XIP	◆	6	294	31,44	8,34
	1115377	Tricitrinoviride_1115377	Fps-like	●	6	315	34,27	6,2
	161710	Tricitrinoviride_161710	Fps-like	▲	6	294	29,62	7,63
	1172378	Tricitrinoviride_1172378	"Other" AQGP	◆	6	336	36,35	6,04
	61920	Tricitrinoviride_61920	classicAQP	●	6	284	30,78	6,03
	1143547	Tricitrinoviride_1143547	classicAQP	▲	6	304	31,49	7,58
	1171100	Tricitrinoviride_1171100	classicAQP	■	6	302	32,26	8,52
Trichoderma gamsii	JPDN01000001.1*	Trigamsii_JPDN01000001.1	XIP	◆	6	301	32,1	8.83
	ANCB02000213.1*	Trigamsii_ANCB02000213.1	Fps-like	●	6	289	35,59	6,89
	JPDN01000042.1*	Trigamsii_JPDN01000042.1	Fps-like	■	6	589	64,83	5,82
	JPDN01000071.1	Trigamsii_JPDN01000071.1	"Other" AQGP	◆	6	344	37,61	6,55
	JPDN01000006.1*	Triga_JPDN01000006.1	classicAQP	●	6	288	31,2	7
	JPDN01000108.1*	Trigamsii_JPDN01000108.1	classicAQP	▲	6	312	32,73	7,81
	JPDN01000082.1*	Trigamsii_JPDN01000082.1	classicAQP	■	6	303	32,55	9,05
Trichoderma guizhouense	LVVK01000020.1*	Triguizhouense_LVVK01000020.1	XIP	◆	6	327	34,8	8,67
	LVVK01000017.1*	Triguizhouense_LVVK01000017.1	Fps-like	●	6	302	32,81	6,87
	LVVK01000019.1b*	Triguizhouense_LVVK01000019.1b	Fps-like	▲	6	274	29,47	7,57
	LVVK01000013.1*	Triguizhouense_LVVK01000013.1	Fps-like	■	6	567	63,33	6,05
	LVVK01000015.1*	Triguizhouense_LVVK01000015.1	"Other" AQGP	◆	6	346	37,63	6,5
	LVVK01000015.1*	Triguizhouense_LVVK01000015.1	classicAQP	●	6	285	30,67	7,77
	LVVK01000018.1*	Triguizhouense_LVVK01000018.1	classicAQP	▲	6	311	32,86	7,77
	LVVK01000004.1	Triguizhouense_LVVK01000004.1	classicAQP	■	6	302	32,41	6,51
Trichoderma hamatum	ANCB02000213.1*	Trihamatum_ANCB02000213.1	XIP	◆	6	301	32,21	8.20
	ANCB02000044.1*	Trihamatum_ANCB02000044.1	Fps-like	●	6	343	32,94	6,88
	ANCB02000059.1*	Trihamatum_ANCB02000059.1	Fps-like	■	6	602	63,06	6
	ANCB02000079.1*	Trihamatum_ANCB02000079.1	"Other" AQGP	◆	6	332	35,75	6,18
	ANCB02000398.1*	Trihamatum_ANCB02000398.1	classicAQP	▲	6	315	33,17	7,74
	ANCB02000229.1*	Trihamatum_ANCB02000229.1	classicAQP	■	6	314	33,7	8,68
Trichoderma harzianum	488926	Triharzianum_488926	XIP	◆	6	294	31,55	8,75
	90014	Triharzianum_90014	Fps-like	●	6	303	32,81	6,87
	490850	Triharzianum_490850	Fps-like	▲	6	274	29,47	7,56
	82211	Triharzianum_82211	Fps-like	■	6	567	63,25	6,09
	92358	Triharzianum_92358	"Other" AQGP	◆	6	346	37,7	6,5
	99286	Triharzianum_99286	classicAQP	●	6	284	30,67	7
	98742	Triharzianum_98742	classicAQP	■	6	302	32,38	7,99
	485859	Triharzianum_485859	classicAQP	▲	6	311	32,84	7,79
Trichoderma koningii	BCGH01000003.1*	Trikoningii_BCGH01000003.1	XIP	◆	6	340	36,23	6,52
	MDJU01000355.1*	Trikoningii_MDJU01000355.1	Fps-like	●	6	302	32,79	6,87
	BCGH01000004.1*	Trikoningii_BCGH01000004.1	Fps-like	▲	6	274	29,44	6,69
	BCGH01000008.1*	Trikoningii_BCGH01000008.1	"Other" AQGP	◆	6	336	37,41	5,79
	BCGH01000002.1*	Trikoningii_BCGH01000002.1	classicAQP	●	6	285	30,67	7,77
	BCGH01000003.1*	Trikoningii_BCGH01000003.1	classicAQP	▲	6	306	31,56	7,87
	BCGH01000009.1*	Trikoningii_BCGH01000009.1	classicAQP	■	6	partial	-	-
Trichoderma longibrachiatum	1343482	Trilongibrachiatum_1343482	XIP	◆	6	294	31,46	7,64
	1339918	Trilongibrachiatum_1339918	Fps-like	●	6	315	34,01	6,51
	1439288	Trilongibrachiatum_1439288	Fps-like	▲	6	275	29,44	7,49
	1362869	Trilongibrachiatum_1362869	"Other" AQGP	◆	6	336	36,29	5,79
	51184	Trilongibrachiatum_51184	classicAQP	●	6	285	30,68	7,77
	1340117	Trilongibrachiatum_1340117	classicAQP	▲	6	306	31,56	7,87
	22769	Trilongibrachiatum_22769	classicAQP	■	6	301	32,05	8,53
Trichoderma pleuroti	MDJU01000245.1*	Tripleuroti_MDJU01000245.1	XIP	◆	6	328	34,74	8,9
	MDJU01000355.1*	Tripleuroti_MDJU01000355.1	Fps-like	●	6	302	32,87	6,87
	MDJU01000027.1*	Tripleuroti_MDJU01000027.1	Fps-like	▲	6	274	29,59	8,47
	MDJU01000063.1*	Tripleuroti_MDJU01000063.1	Fps-like	■	6	567	63,09	6,26
	MDJU01000041.1*	Tripleuroti_MDJU01000041.1	"Other" AQGP	◆	6	346	37,67	6,5
	MDJU01000029.1*	Tripleuroti_MDJU01000029.1	classicAQP	▲	6	304	32,46	6,51
	MDJU01000371.1*	Tripleuroti_MDJU01000371.1	classicAQP	■	6	311	32,73	6,97
Trichoderma reesei	128546	Trireesei_128546	XIP	◆	6	294	31,3	7,69
	92240	Trireesei_92240	Fps-like	●	6	314	34,19	6,7
	141480	Trireesei_141480	Fps-like	▲	6	282	30,46	7,58
	102402	Trireesei_102402	"Other" AQGP	◆	6	336	36,31	6,11
	129514	Trireesei_129514	classicAQP	●	6	287	31,2	6,48
	67036	Trireesei_67036	classicAQP	▲	6	partial	-	-
	143256	Trireesei_143256	classicAQP	■	6	309	32,98	8
Trichoderma virens	8824	Trivirens_8824	XIP	◆	6	294	31,37	8,2
	160074	Trivirens_160074	XIP	◆	6	322	34,3	8
	140684	Trivirens_140684	Fps-like	●	6	303	32,81	6,87
	87413	Trivirens_87413	Fps-like	▲	6	274	29,44	7,59
	41159	Trivirens_41159	Fps-like	■	6	567	63,06	6
	66182	Trivirens_66182	"Other" AQGP	◆	6	300	37,45	6,55
	6760	Trivirens_6760	classicAQP	▲	6	partial	-	-
	228188	Trivirens_228188	classicAQP	■	6	327	35,06	8,59

Fusarium								
Fusarium solani	72760	Necha2_72760	XIP	-	6	343	36,23	7,61
	67872	Necha2_67872	Fps-like	-	6	341	37,12	6,31
Nectria haemotococca	90431	Necha2_90431	classicAQP	-	6	289	32,06	6,58
	51592	Necha2_51592	classicAQP	-	6	266	28,5	5,58
	93338	Necha2_93338	classicAQP	-	7	532	58,95	8,32
Fusarium acuminatum	CBMG010003518.1*	Fusacuminatum_CBMG010003518.1	XIP	-	6	327	35,38	7,62
	CBMG010003683.1*	Fusacuminatum_CBMG010003683.1	Fps-like	-	6	341	37,05	6,43
	CBMG010002146.1*	Fusacuminatum_CBMG010002146.1	Fps-like	-	6	555	61,38	9,1
	CBMG010000926.1*	Fusacuminatum_CBMG010000926.1	classicAQP	-	6	322	34,51	5,96
	CBMG010000883.1*	Fusacuminatum_CBMG010000883.1	classicAQP	-	6	296	32,03	8,83
	CBMG010000225.1*	Fusacuminatum_CBMG010000225.1	classicAQP	-	6	548	60,44	7,04
Fusarium avenaceum	JQGD01000013.1*	Fusavenaceum_JQGD01000013.1	XIP	-	6	327	35,25	7,6
	KIL87348.1*	Fusavenaceum_KIL87348.1	Fps-like	-	6	342	37,07	6,31
	JPYM01000003.1*	Fusavenaceum_JPYM01000003.1	Fps-like	-	6	554	61,13	9,01
	KIL88092.1*	Fusavenaceum_KIL88092.1	classicAQP	-	6	322	34,67	5,67
	KIL87632.1*	Fusavenaceum_KIL87632.1	classicAQP	-	6	291	31,54	8,83
	KIL89865.1*	Fusavenaceum_KIL89865.1	classicAQP	-	6	548	60,4	7,74
Fusarium circinatum	AYJV01002164.1*	Fuscircinata_AYJV01002164.1	XIP	-	6	327	35,47	8,48
	AYJV01002992.1*	Fuscircinata_AYJV01002992.1	Fps-like	-	6	336	36,38	5,66
	JRVE01000048.1*	Fuscircinata_JRVE01000048.1	Fps-like	-	6	548	60,51	8,53
	AYJV01001480.1*	Fuscircinata_AYJV01001480.1	classicAQP	-	6	320	34,27	5,78
	AYJV01003071.1*	Fuscircinata_AYJV01003071.1	classicAQP	-	6	270	28,87	6,21
	AYJV01000611.1*	Fuscircinata_AYJV01000611.1	classicAQP	-	6	539	59,61	7,16
Fusarium culmorum	CBMH010000861.1*	Fusculmorum_CBMH010000861.1	XIP	-	6	330	35,69	7,04
	CBMH010000823.1*	Fusculmorum_CBMH010000823.1	Fps-like	-	6	314	34,14	5,25
	CBMH010000770.1*	Fusculmorum_CBMH010000770.1	Fps-like	-	6	548	60,53	8,68
	CBMH010000147.1*	Fusculmorum_CBMH010000147.1	classicAQP	-	6	316	34,04	6,58
	CBMH010000781.1*	Fusculmorum_CBMH010000781.1	classicAQP	-	6	286	30,76	8,78
	CBMH010001238.1*	Fusculmorum_CBMH010001238.1	classicAQP	-	6	546	59,976	7,73
Fusarium equiseti	CBMI010004636.1*	Fusequiseti_CBMI010004636.1	XIP	-	6	327	35,42	7,65
	CBMI010002872.1*	Fusequiseti_CBMI010002872.1	Fps-like	-	6	335	36,47	5,59
	CBMI010001576.1*	Fusequiseti_CBMI010001576.1	Fps-like	-	6	546	60,27	8,96
	CBMI010000078.1*	Fusequiseti_CBMI010000078.1	classicAQP	-	6	319	34,11	6,02
	CBMI010004009.1*	Fusequiseti_CBMI010004009.1	classicAQP	-	6	266	28,2	5,92
	CBMI010004298.1*	Fusequiseti_CBMI010004298.1	classicAQP	-	6	544	59,73	7,85
Fusarium fujikuroi	ANFV01000069.1*	Fusfujikuroi_ANFV01000069.1	XIP	-	6	327	35,24	6,74
	ANFV01000060.1*	Fusfujikuroi_ANFV01000060.1	Fps-like	-	6	354	38,33	5,43
	JRVG01000144.1*	Fusfujikuroi_JRVG01000144.1	Fps-like	-	6	548	60,39	8,72
	CCT62977.1*	Fusfujikuroi_CCT62977.1	classicAQP	-	6	320	34,3	5,78
	CCT74579.1*	Fusfujikuroi_CCT74579.1	classicAQP	-	6	272	29,03	6,36
	CCT71530.1*	Fusfujikuroi_CCT71530.1	classicAQP	-	6	549	59,47	7,74
Fusarium	5133	Fusgraminearum_5133	XIP	-	6	326	35,37	7,07
graminearum	4917	Fusgraminearum_4917	Fps-like	-	6	336	36,31	5,66
	4321	Fusgraminearum_4321	Fps-like	-	6	548	60,29	8,84
	952	Fusgraminearum_952	classicAQP	-	6	318	34,4	6,44
	4430	Fusgraminearum_4430	classicAQP	-	6	286	30,75	7,76
	12878	Fusgraminearum_12878	classicAQP	-	6	547	60,1	7,73
Fusarium	EKJ77666*	Fuspseudograminearum EKJ77666	XIP	-	6	330	35,69	6,65
pseudograminearum	XP_009259022.1 *	Fuspseudograminearum_XP_009259022.1	Fps-like	-	6	336	36,32	5,66
	JTGC01000315.1*	Fuspseudograminearum_JTGC01000315.1	Fps-like	-	6	548	60,32	8,67
	XP_009261345.1*	Fuspseudograminearum_XP_009261345.1	classicAQP	-	6	318	34,16	6,15
	XP_009253247.1*	Fuspseudograminearum_XP_009253247.1	classicAQP	-	6	286	30,73	8,44
	009260256.1*	Fuspseudograminearum_XP_009260256.1	classicAQP	-	6	547	60,12	7,73
Fusarium oxysporum	FOXG_5361	Fusoxyporum_5361	XIP	-	6	327	35,37	7,11
	FOXG_3231	Fusoxyporum_3231	Fps-like	-	6	331	35,84	5,66
	FOXG_19409	Fusoxyporum_19409	Fps-like	-	6	548	60,27	8,7
	FOXG_1148	Fusoxyporum_1148	classicAQP	-	6	298	31,86	6,02
	FOXG_2910	Fusoxyporum_2910	classicAQP	-	6	273	29,13	6,34
	FOXG_5739	Fusoxyporum_5739	classicAQP	-	6	503	55,7	7,14
	FOXG_12344	Fusoxyporum_12344	classicAQP	-	5	499	54,8	8,61
Fusarium oxysporum	AAXH01000716*	Fuslycopersici_AAXH01000716	XIP	-	6	327	35,37	7,11
lycopersici	EXL45426.1*	Fuslycopersici_EXL45426.1	Fps-like	-	6	388	41,95	5,84
	MALP01000202.1*	Fuslycopersici_MALP01000202.1	Fps-like	-	6	548	60,29	8,23
	AGBH01000103.1*	Fuslycopersici_AGBH01000103.1	classicAQP	-	6	320	34,2	5,83
	EWZ86261.1*	Fuslycopersici_EWZ86261.1	classicAQP	-	6	273	29,11	6,28
	AAXH01000387.1*	Fuslycopersici_AAXH01000387.1	classicAQP	-	6	266	28,1	6,02
	AAXH01000729.1*	Fuslycopersici_AAXH01000729.1	classicAQP	-	6	559	61,53	6,54
Fusarium oxysporum	AGNC01000296.1*	Fusvasinfectum_AGNC01000296.1	XIP	-	6	327	35,4	7,11
vasinfectum	EXM16766.1*	Fusvasinfectum_EXM16766.1	Fps-like	-	6	370	39,98	6,1
	AGNC01000196.1*	Fusvasinfectum_AGNC01000196.1	Fps-like	-	6	548	60,28	8,7
	AGNC01000077.1*	Fusvasinfectum_AGNC01000077.1	classicAQP	-	6	320	34027	5,96
	AGNC01000348.1*	>Fusvasinfectum_AGNC01000348.1	classicAQP	-	6	273	29,19	6,34
	EXM26604.1*	Fusvasinfectum_EXM26604.1	classicAQP	-	6	559	61,53	6,54
Fusarium	AAIM02000133.1*	Fusverticillioides_AAIM02000133.1	XIP	-	6	327	35,35	8,78
verticilloides	EWG48857.1*	Fusverticillioides_EWG48857.1	Fps-like	-	6	331	35,82	5,66
	AAIM02000170.1*	Fusverticillioides_AAIM02000170.1	Fps-like	-	6	547	60,33	8,72
	EWG36264.1*	Fusverticillioides_EWG36264.1	classicAQP	-	6	320	34,26	5,91
	EWG49201.1*	Fusverticillioides_EWG49201.1	classicAQP	-	6	273	29,19	6,28
	EWG52904.1*	Fusverticillioides_EWG52904.1	classicAQP	-	6	559	61,52	6,35
Fusarium	AEYB01001170.1*	Fusvirguliforme_AEYB01001170.1	XIP	-	6	320	34,7	7,66
virguliform	AEYB01000058.1*	Fusvirguliforme_AEYB01000058.1	Fps-like	-	6	325	35,04	6,29
	AEYB01001186.1*	Fusvirguliforme_AEYB01001186.1	classicAQP	-	6	266	28,09	7,05
	AEYB01000674.1*	Fusvirguliforme_AEYB01000674.1	classicAQP	-	6	265	28,13	5,91
	AEYB01000247.1*	Fusvirguliforme_AEYB01000247.1	classicAQP	-	6	552	60,68	6,77

Laccaria bicolor								
Laccaria bicolor	317173	Lacbi2_317173	AQGP	-	6	332	35,82	9,13
	443240	Lacbi2_443240	AQGP	-	6	312	33,92	5,76
	576801	Lacbi2_576801	AQGP	-	6	263	28,36	8,55
	568479	Lacbi2_568479	AQGP	-	6	263	28,3	9,17
	671860	Lacbi2_671860	"Other" AQGP	-	6	330	35,79	7,69
	482072	Lacbi2_482072	"Other" AQGP	-	6	343	37,13	8,17
	456764	Lacbi2_456764	classicAQP	-	6	311	32,65	5,44
Mycosphaerella fijiensis								
Mycosphaerella fijiensis	173306	Mycfi2_173306	XIP	-	6	394	42,25	8,43
	108013	Mycfi2_108013	AQGP	-	6	589	64,3	8,7
	33768	Mycfi2_33768	AQGP	-	6	309	3306	6,08
	133800	Mycfi2_133800	AQGP	-	6	311	34,621	8,14
	99980	Mycfi2_99980	AQGP	-	6	321	34,79	6,96
	165933	Mycfi2_165933	"Other" AQGP	-	6	349	38,23	6,79
	99603	Mycfi2_99603	classicAQP	-	6	275	29,27	9,69
	134906	Mycfi2_134906	classicAQP	-	6	261	28,07	5,03
	120962	Mycfi2_120962	classicAQP	-	6	264	27,89	6,9
	76398	Mycfi2_76398	classicAQP	-	6	282	29,93	8,63
	100823	Mycfi2_100823	classicAQP	-	6	271	29,42	8,57
