# Supplementary material for: MIP diversity from Trichoderma: Structural considerations and transcriptional modulation during mycoparasitic association with Fusarium solani olive trees
Source: PLoS One. 2018 Mar 15;13(3):e0193760. doi: 10.1371/journal.pone.0193760 (PMC5854309; doi:10.1371/journal.pone.0193760)
Supplement: S2 Table — (PDF) [file pone.0193760.s005.pdf]

S2 Table. Primers used for qPCR amplification.

		Nomenclature used in this work	Accession number [JGI] / [NCBI]*	Primer sequences [5' to 3' ; Forward / Reverse]	Annealing [°C]
Trichoderma harzianum	**18S rDNA**	/	18SrDNA -KT897696.1 *	GGTGGAGTGATTTGTCTG / CTTACTAGGGATTCCTCG	52
	**Elongation factor 1-alpha**	/	146236	CGCCATTGCTCGTGTTGACATG / AATTTCTCGCTCGGCGGTGGTG	54
	**Tubulin**	/	134768	GTACTAAGTTGTTTCTTTGCTGTTG / CTCTTGTACATACACCAATTGCTC	60
	**Aquaporin**	Th99286	99286	CGCCAGTATGTGTGCAGGCG / GCGCCGGTGTAAGAAGTCCC	56
		Th98742	98742	CCGCAGCAGTCACCGATGCC / CTGGTAGCCCGTGAAGTCGGTG	56
		Th485859	485859	GCCGCAGCGACCGTATCAGG / TATCCCCTGAAGCTGCCCGTC	56
	**Aquaglyceroporin**	Th90014	90014	GTTCTGCATCTTCGCCCTGGC / CATATCCAATCATGAACGACACC	56
		Th490850	490850	CTCTATGCTCTGCTAGACGATGG / GACTTGGTGTCCATAACCAACTG	56
		Th82211	82211	CTGCTTTGTCACAAGCCAGCGC / GAGGTCCAAAATCGCGGCTCGG	56
		Th92358	92358	CACCATGAACATGGCTCGAGAC / CCTCACCAAGGCCTCATCTCC	56
	**X-Intrinsic Protein**	Th488926	488926	GGCGGYTGYTGGTACGACCC / TCATCTGCGCTCCAGCRTAGCC	60
***Fusarium solani* (Necha2)**	**18S rDNA**	/	18SrDNA -JQ837837.1 *	GTATTGCTTTGGCAGTACGCT / GGCCTTTCGGCCAAGGAAGT	56
	**Actin**	/	63567	CGCCATTGCTCGTGTTGACATG / AATTTCTCGCTCGGCGGTGGTG	56
	**Tubulin**	/	98894	GAGTACTAAGCGGTTTCGGATGC / GCATGCCGCGTGCGCGCAAGGTTC	58
	**Aquaporin**	Necha2_90431	90431	GTGGCTCGAATACCATACTGCC / GCGACGGGGCTCATCTGGC	56
		Necha2_51592	51592	CGGGTGGCTCTGCAAACCC / CCTCATGAGGACTGTCCTG	56
		Necha2_93338	93338	GGATCTCTCATCGCTGTGGCG / CGGATCGAAGGCGACTTGCCC	56
	**Aquaglyceroporin**	Necha2_67872	67872	GATCGGATACGGAACTGAGG / CCAGGGCGCGGTCATAGGTG	58
	**X-Intrinsic Protein**	Necha2_72760	72760	GATTTACACGATATGGATATGGCGG / GTTCTCCTTGGTCCAGGGTGG	60
