# Supplementary material for: MIP diversity from Trichoderma: Structural considerations and transcriptional modulation during mycoparasitic association with Fusarium solani olive trees
Source: PLoS One. 2018 Mar 15;13(3):e0193760. doi: 10.1371/journal.pone.0193760 (PMC5854309; doi:10.1371/journal.pone.0193760)
Supplement: S3 Table — (PDF) [file pone.0193760.s006.pdf]

S3 Table. Detail of the TFBS nucleotide sites found on 1.5kb of each promoter of the four expressed MIP.

XIP					AQP				
Sens	Position	Motif	Bases	Cutoff	Sens	Position	Motif	Bases	Cutoff
+	(5, 16)	ROX1	ACCATTGTCTTC	0.80	+	(3, 14)	UASPHR	ATTGCTACCTCG	0.86
+	(13, 17)	GCR1	CTTCC	1.00	-	(53, 59)	PHO2	CTAAGTG	0.83
+	(15, 27)	CSRE	TCCAGATGACTGG	0.81	-	(76, 87)	UASPHR	TGTTCTGCCTCG	0.89
+	(21, 26)	GCN4	TGACTG	0.92	-	(77, 82)	GCN4	TGCCTC	0.80
-	(27, 32)	GCN4	TGGCTC	0.80	+	(90, 99)	PHO4	AGCACCTTGT	0.81
-	(44, 51)	STE12	ATGACACT	0.83	-	(96, 107)	RAP1	GCACCCTCACAA	0.84
-	(45, 50)	GCN4	TGACAC	0.81	+	(113, 118)	GCN4	TGAATC	0.86
-	(69, 80)	SWI5	ACAAGACGCTGG	0.82	+	(146, 150)	GCR1	CATCC	0.93
+	(76, 87)	SWI5	CTTGTCTGCTGG	0.84	+	(150, 154)	GCR1	CCTCC	0.86
-	(77, 85)	repressor_of_CAR1	AGCAGACAA	0.82	+	(164, 172)	repressor_of_CAR1	AGCCGCTGC	0.92
-	(136, 140)	GCR1	CCTCC	0.86	+	(175, 179)	ADR1	TCTCC	0.89
+	(147, 158)	MIG1	TCCCAGATTCTT	0.81	+	(199, 207)	repressor_of_CAR1	AACCGGCGC	0.81
-	(187, 195)	repressor_of_CAR1	AGCCACCCA	0.99	+	(207, 216)	LEU3	CCGCATTCGG	0.89
-	(215, 221)	SCB	CCCGAAA	0.91	-	(213, 221)	repressor_of_CAR1	AGAGGCCGA	0.81
+	(220, 225)	GCN4	GGACTC	0.80	+	(232, 241)	PHO4	TGCACCTGGT	0.81
-	(230, 241)	XBP1	AGCTCGAGCCGA	0.85	+	(243, 252)	PHO4	AGAACGTGGG	0.83
-	(238, 245)	STE12	ATGAAGCT	0.83	-	(243, 252)	PHO4	CCCACGTTCT	0.88
-	(245, 253)	repressor_of_CAR1	CGGCGCCGA	0.82	+	(270, 275)	MCB	ACGCGT	1.00
-	(264, 272)	repressor_of_CAR1	AGCCACGAC	0.84	-	(270, 275)	MCB	ACGCGT	1.00
-	(321, 326)	GCN4	TGACTT	0.91	+	(305, 309)	GCR1	CGTCC	0.86
-	(346, 355)	MCM1	GCTGATTAGG	0.80	-	(310, 315)	GCN4	TAACTC	0.81
-	(348, 353)	GCN4	TGATTA	0.80	+	(316, 324)	repressor_of_CAR1	ATCCACCCA	0.86
+	(384, 388)	ADR1	TCTCC	1.00	-	(330, 336)	PHO2	CTAACTG	0.83
+	(390, 394)	GCR1	CTTCC	1.00	-	(346, 354)	repressor_of_CAR1	AGCTGACGA	0.82
+	(396, 404)	repressor_of_CAR1	CGCCGCCCT	0.86	-	(412, 418)	PHO2	CTAAAGG	0.80
+	(420, 425)	MCB	ACGCAT	0.82	+	(445, 449)	GCR1	CATCC	0.93
-	(420, 425)	MCB	ATGCGT	0.82	+	(466, 472)	BAS2	TAACTAA	0.82

+	(429, 433)	GCR1	CTTCC	1.00	-	(463, 468)	GCN4	TTACTC	0.82
+	(449, 455)	REB1	TTACCCG	1.00	+	(470, 476)	TBP	TAAAAAA	0.84
+	(451, 456)	MCB	ACCCGT	0.82	+	(479, 484)	ACE2	GCTGGT	1.00
-	(451, 456)	MCB	ACGGGT	0.82	-	(489, 496)	STE12	ATCAAACT	0.83
+	(461, 468)	PDR1/PDR3	TCCCTGGA	0.82	-	(497, 505)	repressor_of_CAR1	ACCCGCCGG	0.92
-	(529, 535)	PHO2	TCAAATG	0.83	-	(501, 507)	REB1	ATACCCG	0.87
-	(538, 543)	GCN4	TGACGC	0.82	-	(507, 512)	BAS2	TAATAA	0.86
+	(557, 563)	PHO2	CTACATT	0.80	-	(532, 540)	repressor_of_CAR1	AGCCTCCAA	1.00
-	(560, 566)	PHO2	TTCAATG	0.83	-	(534, 538)	GCR1	CCTCC	0.86
+	(565, 573)	repressor_of_CAR1	AACTGCCCA	0.82	-	(540, 546)	SCB	CACCAAA	0.85
+	(571, 582)	SWI5	CCATCATGCTGG	0.93	-	(545, 552)	STE12	ATGTAACA	0.83
+	(584, 592)	MATalpha2	CATGTATCT	0.83	+	(547, 553)	PHO2	TTACATT	0.83
-	(598, 602)	GCR1	CTTCC	1.00	+	(559, 564)	GCN4	TGACGC	0.82
-	(622, 630)	repressor_of_CAR1	AGACGCCGA	0.96	+	(561, 566)	MCB	ACGCAT	0.82
-	(631, 635)	GCR1	CGTCC	0.86	-	(561, 566)	MCB	ATGCGT	0.82
+	(632, 638)	SCB	GACGAAA	0.85	+	(566, 572)	TBP	TACAAAA	0.84
+	(634, 640)	PHO2	CGAAATG	0.80	+	(576, 580)	GCR1	CTTCC	1.00
-	(643, 649)	SCB	CACGAGA	0.85	-	(597, 603)	PHO2	CTACATT	0.80
+	(651, 660)	PHO4	CGGACGTGCA	0.81	+	(610, 616)	SCB	AACGAAA	0.85
-	(651, 660)	PHO4	TGCACGTCCG	0.83	-	(618, 624)	REB1	TAACCCG	0.90
-	(652, 656)	GCR1	CGTCC	0.86	-	(623, 629)	TBP	TATAATA	0.84
-	(692, 696)	GCR1	CATCC	0.93	-	(622, 627)	BAS2	TAATAA	0.85
-	(721, 729)	repressor_of_CAR1	AACCACCCA	0.88	+	(626, 632)	TBP	TATAGAA	0.89
-	(755, 761)	RAP1	ACACCCA	0.98	+	(628, 634)	TBP	TAGAAAA	0.84
-	(759, 766)	STE12	AAGAAACA	0.83	+	(647, 658)	SWI5	ATACACGGCTGG	0.84
-	(767, 773)	SCB	AACGAAA	0.85	-	(648, 656)	repressor_of_CAR1	AGCCGTGTA	0.82
+	(798, 803)	GCN4	TGATTC	0.87	+	(680, 686)	TBP	TATAAGA	0.84
+	(802, 809)	PDR1/PDR3	TCCCTGGA	0.82	+	(689, 694)	MCB	ACGCAT	0.82
-	(811, 819)	repressor_of_CAR1	GGCTGCCAA	0.82	-	(689, 694)	MCB	ATGCGT	0.82
+	(816, 824)	repressor_of_CAR1	AGCCAGCAG	0.82	+	(699, 705)	PHO2	CTACATG	0.80
-	(818, 829)	SWI5	AAATCCTGCTGG	0.91	-	(736, 740)	GCR1	CATCC	0.93
-	(826, 832)	TBP	TACAAAT	0.82	-	(771, 777)	PHO2	CTAAAAG	0.80
+	(865, 871)	PHO2	CTTAATG	0.80	-	(772, 778)	TBP	TCTAAAA	0.84
-	(872, 883)	ROX1	TCAAATGTTCAC	0.80	-	(792, 797)	BAS2	TAATAA	0.86

-	(877, 883)	PHO2	TCAAATG	0.83	+	(793, 799)	TBP	TATTAAA	0.86
+	(881, 886)	GCN4	TGACAC	0.81	+	(794, 801)	STE12	ATTAAACA	0.83
-	(887, 893)	PHO2	GTAAGTT	0.80	-	(802, 808)	PHO2	CTATATG	0.80
+	(978, 986)	repressor_of_CAR1	AACAGCCAA	0.86	+	(804, 810)	TBP	TATAGAA	0.89
-	(988, 992)	GCR1	CTTCC	1.00	+	(811, 817)	PHO2	CTGAATG	0.80
-	(1018, 1025)	STE12	ATGACACT	0.83	-	(826, 831)	GCN4	TGAATC	0.86
-	(1019, 1024)	GCN4	TGACAC	0.81	-	(873, 878)	GCN4	TAACTC	0.81
+	(1025, 1030)	GCN4	TGATTA	0.80	-	(876, 881)	BAS2	TAATAA	0.87
-	(1047, 1053)	TBP	TAAAAAT	0.82	+	(895, 900)	GCN4	GGACTC	0.80
-	(1052, 1057)	GCN4	TGATTA	0.80	-	(896, 901)	GCN4	TGAGTC	0.87
-	(1094, 1100)	PHO2	TTAACTT	0.86	+	(908, 912)	GCR1	CTTCC	1.00
+	(1097, 1103)	PHO2	TTAAATA	0.91	+	(955, 966)	SWI5	AATTCTTGCTGG	0.86
-	(1097, 1103)	TBP	TATTTAA	0.80	+	(971, 979)	MATalpha2	CTTGTAAAT	0.87
+	(1103, 1111)	repressor_of_CAR1	AGCCTACTA	0.82	+	(973, 979)	TBP	TGTAAAT	0.82
-	(1108, 1116)	MATalpha2	CATGTTAGT	0.82	+	(974, 980)	PHO2	GTAAATG	0.94
+	(1117, 1125)	repressor_of_CAR1	AGCCGGGCA	0.84	+	(987, 993)	PHO2	CTATATG	0.80
+	(1131, 1135)	ADR1	TCTCC	0.82	+	(992, 998)	TBP	TGTAAAA	0.84
-	(1135, 1141)	PHO2	CCAAATG	0.80	+	(1029, 1035)	TBP	GATAAAA	0.84
-	(1150, 1159)	PHO4	AGCATGTGGA	0.82	-	(1030, 1041)	MIG1	CGCCAGTTTTAT	0.82
+	(1150, 1161)	SWI5	TCCACATGCTGG	0.82	+	(1030, 1037)	STE12	ATAAAACT	0.83
+	(1163, 1171)	repressor_of_CAR1	AGCTGCTGA	0.83	+	(1041, 1047)	PHO2	GTAAATA	0.86
+	(1175, 1184)	PHO4	CCCACGTAGA	0.84	+	(1046, 1052)	TBP	TACAAAA	0.84
-	(1189, 1195)	SCB	CACGGAA	0.85	+	(1055, 1059)	GCR1	CCTCC	0.86
+	(1255, 1274)	SMP1	GCTGCTACTACTAGCC	0.81	+	(1060, 1064)	GCR1	CCTCC	0.86
-	(1274, 1279)	BAS2	TAATAA	0.91	-	(1060, 1071)	XBP1	GGGTCGAGGAGG	0.87
+	(1303, 1308)	MCB	ACGGGT	0.82	-	(1104, 1112)	repressor_of_CAR1	AGCAGTCAA	0.82
-	(1303, 1308)	MCB	ACCCGT	0.82	+	(1105, 1110)	GCN4	TGACTG	0.92
-	(1304, 1310)	REB1	ATACCCG	0.87	+	(1111, 1115)	GCR1	CTTCC	1.00

-	(1335, 1339)	ADR1	TCTCC	1.00
-	(1343, 1348)	GCN4	TGCCTC	0.80
+	(1359, 1364)	GCN4	TCACTC	0.81
-	(1369, 1375)	PHO2	TTAAACT	0.83
-	(1381, 1387)	PHO2	TTGAATG	0.83
-	(1396, 1401)	GCN4	CGACTC	0.80
-	(1407, 1411)	ADR1	TCTCC	0.81
+	(1416, 1422)	RAP1	GCACCCA	0.87
-	(1420, 1431)	SWI5	GTCGCCGGCTGG	0.81
+	(1422, 1430)	repressor_of_CAR1	AGCCGGCGA	0.99
+	(1424, 1433)	LEU3	CCGGCGACGG	0.87
+	(1430, 1435)	MCB	ACGGGT	0.82
-	(1430, 1435)	MCB	ACCCGT	0.82
+	(1466, 1482)	GAL4	GGACCAGAGACCTGC	0.81
-	(1466, 1482)	GAL4	GCAGGTCTCTGGTCC	0.81

+	(1121, 1129)	repressor_of_CAR1	AGCAGCCGT	0.88
-	(1135, 1147)	CSRE	TTCTCATGAATGG	0.81
-	(1152, 1161)	MCM1	CCACAAAAGG	0.81
-	(1154, 1160)	SCB	CACAAAA	0.85
-	(1155, 1163)	repressor_of_CAR1	AGCCACAAA	0.89
+	(1170, 1179)	MCM1	CTCAATCAGG	0.80
+	(1209, 1214)	GCN4	TGCCTC	0.80
-	(1252, 1263)	ROX1	CACATTCTTCTC	0.82
+	(1292, 1297)	BAS2	TAATAA	0.94
-	(1330, 1336)	SCB	AACGAAA	0.85
+	(1352, 1357)	MCB	ACCCGT	0.82
-	(1352, 1357)	MCB	ACGGGT	0.82
-	(1372, 1389)	RLM1	ATTCTATATATACTGC	0.80
-	(1376, 1382)	TBP	TATATAC	0.86
+	(1377, 1383)	TBP	TATATAT	0.93
-	(1378, 1384)	TBP	TATATAT	0.93
+	(1379, 1385)	TBP	TATATAG	0.87
+	(1381, 1387)	TBP	TATAGAA	0.89
+	(1398, 1402)	ADR1	TCTCC	0.87
+	(1394, 1405)	UASPHR	GTTCTCTCCTCG	0.80
-	(1415, 1420)	BAS2	TAATAA	0.86
-	(1435, 1440)	GCN4	TGAGTA	0.80
+	(1448, 1452)	GCR1	CATCC	0.93
+	(1466, 1472)	TBP	TATACAG	0.84
+	(1489, 1497)	repressor_of_CAR1	AACTGCCCA	0.82

92358				
Sens	Position	Motif	Bases	Cutoff
+	(15, 19)	GCR1	CTTCC	1.00
+	(23, 28)	GCN4	TGAGTC	0.87
-	(24, 29)	GCN4	TGACTC	1.00
+	(47, 52)	GCN4	TGCCTC	0.80
+	(47, 55)	repressor_of_CAR1	TGCCTCCAA	0.88
+	(49, 53)	GCR1	CCTCC	0.86
+	(58, 66)	repressor_of_CAR1	AACCACCCA	0.88
-	(74, 79)	GCN4	TGACTT	0.91
+	(77, 84)	PDR1/PDR3	TCAGCGGA	0.87
-	(77, 84)	PDR1/PDR3	TCCGCTGA	0.87
+	(95, 99)	GCR1	CGTCC	0.86
-	(98, 106)	repressor_of_CAR1	AGCCAACGG	0.81
+	(102, 107)	GCN4	TGGCTC	0.80
+	(106, 117)	ABF1	TCGCCAGAAACG	0.82
-	(111, 122)	MIG1	CCCCGCGTTTCT	0.86
+	(115, 120)	MCB	ACGCGG	0.87
-	(115, 120)	MCB	CCGCGT	0.87
+	(128, 134)	PHO2	CCAAATG	0.80
-	(136, 142)	PHO2	CTAAACG	0.80
+	(155, 164)	PHO4	AGCCCGTTGG	0.81
+	(163, 168)	GCN4	GGACTC	0.80
-	(164, 169)	GCN4	TGAGTC	0.87
-	(167, 174)	PDR1/PDR3	TCCGTTGA	0.81
-	(173, 179)	PHO2	TTAATTC	0.80
-	(174, 180)	PHO2	TTTAATT	0.83
+	(175, 182)	STE12	ATTAAACT	0.83
+	(176, 182)	PHO2	TTAAACT	0.83

90014				
Sens	Position	Motif	Bases	Cutoff
-	(6, 14)	repressor_of_CAR1	GGCCTCCCA	0.83
-	(8, 12)	GCR1	CCTCC	0.86
+	(41, 50)	PHO4	CGCACTTGTG	0.84
-	(75, 84)	MCM1	CCCGATTAGA	0.84
+	(81, 96)	PUT3	CGGGATATGCACGCC[illegible]	0.95
+	(100, 104)	GCR1	CATCC	0.93
-	(123, 131)	repressor_of_CAR1	AGCCGGCCC	0.88
-	(128, 135)	STE12	ATGAAGCC	0.86
+	(133, 141)	MATalpha2	CATGTATGT	0.83
+	(167, 173)	PHO2	CTTAATG	0.80
+	(181, 186)	SWI5	TGCTGA	0.86
-	(193, 204)	ABF1	TCACAAACAACA	0.82
-	(206, 213)	STE12	ATGGAACC	0.86
+	(243, 251)	repressor_of_CAR1	AGCCCCTCA	0.83
+	(272, 283)	UASPHR	TTTCTTTCTTCG	0.83
+	(275, 286)	UASPHR	CTTTCTTCGTCT	0.81
+	(288, 299)	UASPHR	TTTTCTCCTTCT	0.81
+	(291, 295)	ADR1	TCTCC	
+	(297, 301)	ADR1	TCTCC	
-	(334, 340)	SCB	CAAGAAA	0.85
-	(349, 357)	repressor_of_CAR1	AGCAGCGCA	0.82
+	(360, 365)	GCN4	TGTCTC	0.80
+	(364, 369)	GCN4	TCACTC	0.81
-	(391, 397)	PHO2	CAAAATT	0.80
+	(398, 402)	GCR1	CATCC	0.93
+	(413, 422)	PHO4	GGCGCGTGGA	0.82
+	(414, 419)	MCB	GCGCGT	0.85

-	(223, 234)	MIG1	CCCCACACAAAC	0.82	-	(414, 419)	MCB	ACGCGC	0.87
+	(274, 278)	ADR1	TCTCC	0.89	-	(431, 436)	GCN4	TGACTA	0.94
+	(277, 288)	MIG1	CCCCACACATCC	0.82	+	(463, 469)	SCB	CACGAGA	0.85
+	(284, 288)	GCR1	CATCC	0.93	-	(468, 473)	GCN4	AGACTC	0.80
+	(285, 293)	repressor_of_CAR1	ATCCACCAT	0.81	-	(518, 522)	GCR1	CCTCC	0.86
-	(304, 310)	REB1	TTACCCC	0.87	+	(528, 534)	TBP	TATGAAA	0.85
+	(307, 313)	PHO2	GTAAATT	0.94	+	(529, 536)	STE12	ATGAAATG	0.83
-	(313, 320)	PDR1/PDR3	CCCGTGGA	0.81	+	(530, 536)	PHO2	TGAAATG	0.83
-	(313, 322)	PHO4	CGCCCGTGGA	0.84	+	(543, 547)	GCR1	CCTCC	0.86
+	(340, 348)	repressor_of_CAR1	TGCCGACAA	0.84	+	(549, 553)	GCR1	CTTCC	1.00
+	(346, 352)	PHO2	CAAAATT	0.80	+	(554, 558)	GCR1	CCTCC	0.86
-	(361, 369)	repressor_of_CAR1	AACAGCCCA	0.82	+	(575, 579)	GCR1	CATCC	0.93
-	(387, 398)	MIG1	CCCAACATTGTT	0.81	+	(584, 589)	GCN4	TTACTC	0.82
-	(391, 402)	RAP1	CCACCCCAACAT	0.84	+	(584, 590)	REB1	TTACTCG	0.85
-	(396, 404)	repressor_of_CAR1	AGCCACCCC	0.92	+	(586, 591)	MCB	ACTCGT	0.82
-	(439, 445)	PHO2	CTAAAAG	0.80	-	(586, 591)	MCB	ACGAGT	0.82
+	(444, 452)	repressor_of_CAR1	AGCCTCCTG	0.89	+	(612, 618)	REB1	TTGCCCG	0.85
+	(446, 450)	GCR1	CCTCC	0.86	+	(624, 628)	GCR1	CTTCC	1.00
+	(453, 461)	MATalpha2	CCTGTAAGT	0.82	+	(628, 632)	GCR1	CGTCC	0.86
+	(479, 484)	GCN4	TGACTT	0.91	-	(633, 639)	TBP	TAAAAAA	0.84
+	(485, 490)	GCN4	TGGCTC	0.80	-	(691, 697)	PHO2	CTCAATT	0.80
+	(561, 572)	SWI5	AGTTTCTGCTGG	0.89	-	(696, 702)	REB1	TCACCCT	0.80
-	(569, 575)	REB1	TTACCAG	0.85	-	(703, 712)	MCM1	CCGAATCAGG	0.89
-	(583, 587)	GCR1	CTTCC	1.00	+	(705, 710)	GCN4	TGATTC	0.87
-	(600, 604)	ADR1	TCTCC	0.85	+	(717, 725)	repressor_of_CAR1	AGCCTCCGA	1.01
-	(624, 628)	GCR1	CGTCC	0.86	+	(719, 723)	GCR1	CCTCC	0.86
+	(641, 646)	MCB	ACGCAT	0.82	+	(730, 735)	MCB	GCGCGT	0.85
-	(641, 646)	MCB	ATGCGT	0.82	-	(730, 735)	MCB	ACGCGC	0.87
-	(655, 660)	GCN4	TGACTT	0.91	+	(783, 794)	MIG1	CCCCAGGTCGTT	0.81
+	(660, 666)	TBP	AATAAAA	0.84	-	(816, 823)	STE12	ATGAAATC	0.90
+	(678, 682)	GCR1	CTTCC	1.00	+	(824, 828)	GCR1	CTTCC	1.00
-	(694, 701)	PDR1/PDR3	TACGTGGA	0.81	+	(831, 837)	REB1	TCACCCG	0.92
+	(696, 702)	SCB	CACGTAA	0.85	+	(833, 838)	MCB	ACCCGT	0.82
+	(700, 705)	BAS2	TAATGA	0.84	-	(833, 838)	MCB	ACGGGT	0.82

-	(705, 711)	PHO2	TCAAATT	0.83	+	(836, 840)	GCR1	CGTCC	0.86
-	(707, 713)	TBP	TATCAAA	0.85	-	(879, 884)	GCN4	TAACTC	0.81
-	(721, 730)	PHO4	GGCAAGTGTT	0.82	+	(902, 907)	GCN4	TGATTC	0.87
+	(739, 747)	repressor_of_CAR1	AGCCACCAG	0.95	-	(902, 909)	STE12	ATGAATCA	0.83
-	(763, 769)	REB1	TTGCCCG	0.85	-	(903, 908)	GCN4	TGAATC	0.86
-	(770, 779)	MCM1	CCCCATTAGT	0.83	+	(909, 915)	REB1	TTTCCCG	0.85
-	(776, 784)	repressor_of_CAR1	AGCGGCCCC	0.86	+	(946, 950)	GCR1	CATCC	0.93
-	(804, 808)	GCR1	CCTCC	0.86	+	(949, 960)	MIG1	CCCCGTATTTGA	0.83
+	(840, 851)	UASPHR	TCTTAGTCCTCG	0.80	-	(963, 969)	REB1	TTCCCCG	0.85
+	(851, 862)	XBP1	GTCTCGCAGCGG	0.83	-	(966, 970)	GCR1	CTTCC	1.00
+	(875, 880)	GCN4	TGACTT	0.91	+	(969, 978)	PHO4	AGAACGTGTT	0.82
+	(880, 885)	GCN4	TGCCTC	0.80	-	(969, 978)	PHO4	AACACGTTCT	0.86
+	(938, 945)	STE12	ATGCAACC	0.86	+	(972, 977)	MCB	ACGTGT	0.82
+	(944, 956)	CSRE	CCCGGAGAGATGG	0.81	-	(972, 977)	MCB	ACACGT	0.82
-	(947, 951)	ADR1	TCTCC	0.91	+	(1047, 1051)	GCR1	CGTCC	0.86
+	(958, 964)	PHO2	CTGAATT	0.80	-	(1075, 1083)	repressor_of_CAR1	AGCCGAAGA	0.84
-	(988, 992)	GCR1	CCTCC	0.86	-	(1099, 1107)	repressor_of_CAR1	AGACGCAAA	0.83
-	(996, 1000)	GCR1	CCTCC	0.86	+	(1119, 1125)	REB1	TTACCCG	1.00
+	(998, 1006)	repressor_of_CAR1	AGGGGCCAA	0.81	-	(1134, 1142)	repressor_of_CAR1	AGCAGCAGA	0.83
-	(1022, 1028)	REB1	TTACCCC	0.87	+	(1145, 1149)	GCR1	CATCC	0.93
+	(1063, 1071)	repressor_of_CAR1	AGTCGCCTC	0.84	-	(1150, 1155)	SWI5	GGCTGG	0.83
+	(1066, 1074)	repressor_of_CAR1	CGCCTCCAA	0.87	-	(1154, 1165)	SWI5	AATTGAGGCTGG	0.82
+	(1068, 1072)	GCR1	CCTCC	0.86	+	(1159, 1165)	PHO2	CTCAATT	0.80
-	(1075, 1083)	repressor_of_CAR1	GGCCGCCCA	0.94	+	(1160, 1171)	ROX1	TCAATTGTTATA	0.84
-	(1096, 1103)	STE12	ATGAAACG	0.93	-	(1165, 1171)	TBP	TATAACA	0.84
-	(1098, 1104)	SCB	CATGAAA	0.85	-	(1177, 1183)	REB1	TTACCCA	0.87
+	(1114, 1122)	repressor_of_CAR1	AGCCACCTG	0.90	+	(1189, 1200)	XBP1	CCCACGAGGAGA	0.80
-	(1133, 1137)	ADR1	TCTCC	0.86	-	(1196, 1200)	ADR1	TCTCC	0.82

+	(1182, 1190)	repressor_of_CAR1	TGCCACCAA	0.89
-	(1195, 1200)	GCN4	TGAATC	0.86
+	(1196, 1207)	SWI5	ATTCAAAGCTGG	0.81
-	(1198, 1209)	MIG1	CCCCAGCTTTGA	0.84
-	(1207, 1215)	repressor_of_CAR1	AGACGCCCC	0.86
-	(1214, 1225)	MIG1	CCCCAGAAATAG	0.83
-	(1224, 1228)	GCR1	CCTCC	0.86
+	(1267, 1275)	repressor_of_CAR1	AGCTTCCGA	0.86
+	(1269, 1273)	GCR1	CTTCC	1.00
-	(1321, 1325)	GCR1	CATCC	0.93
+	(1323, 1328)	MCB	ATGCGT	0.82
-	(1323, 1328)	MCB	ACGCAT	0.82
+	(1333, 1338)	GCN4	TGACTA	0.94
-	(1357, 1368)	SWI5	GTTTCATGCAGG	0.81
+	(1361, 1367)	SCB	CATGAAA	0.85
+	(1362, 1369)	STE12	ATGAAACT	0.97
-	(1368, 1374)	TBP	TATATAG	0.87
+	(1369, 1375)	TBP	TATATAG	0.87
-	(1374, 1381)	STE12	ATGAGACT	0.83
+	(1381, 1386)	GCN4	TGCCTC	0.80
+	(1386, 1390)	GCR1	CTTCC	1.00
-	(1405, 1411)	SCB	CAAGAAA	0.85
+	(1412, 1423)	UASPHR	TCTTCTTCCTCC	0.90
+	(1416, 1420)	GCR1	CTTCC	1.00
+	(1419, 1423)	GCR1	CCTCC	0.86
-	(1423, 1429)	PHO2	TTGAATG	0.83
+	(1428, 1436)	repressor_of_CAR1	AAGCGCCTA	0.81
-	(1433, 1442)	MCM1	GCCAATTAGG	0.88
+	(1434, 1440)	PHO2	CTAATTG	0.86
-	(1444, 1450)	TBP	TATCAAA	0.85
+	(1449, 1455)	TBP	TATACAC	0.82
+	(1463, 1470)	STE12	GTGAAACA	0.83
-	(1473, 1479)	PHO2	GTAACTT	0.80
-	(1488, 1493)	GCN4	TGACTT	0.91

-	(1217, 1224)	STE12	AGGAAACC	0.86
+	(1225, 1233)	repressor_of_CAR1	AACCGCGGG	0.83
-	(1230, 1236)	REB1	TCACCCG	0.92
+	(1258, 1266)	repressor_of_CAR1	AGCCTGCAA	0.88
+	(1286, 1292)	TBP	TAAAAAA	0.84
+	(1294, 1300)	TBP	TATAATT	0.82
+	(1295, 1301)	PHO2	ATAATTT	0.80
-	(1297, 1303)	PHO2	TCAAATT	0.83
+	(1309, 1313)	GCR1	CCTCC	0.86
+	(1317, 1321)	GCR1	CCTCC	0.86
+	(1327, 1334)	STE12	TTGAAACA	0.83
+	(1338, 1349)	MIG1	CCCCAATTCTTT	0.83
+	(1365, 1376)	RAP1	ACACACATACAT	0.88
+	(1386, 1394)	repressor_of_CAR1	AGCATCCCA	0.82
+	(1388, 1392)	GCR1	CATCC	0.93
+	(1408, 1415)	STE12	AAGAAACA	0.83
+	(1417, 1423)	PHO2	CTGAATT	0.80
+	(1423, 1427)	ADR1	TCTCC	0.86
+	(1436, 1444)	repressor_of_CAR1	AGCCGCTCC	0.88
+	(1442, 1453)	UASPHR	TCCTCTTCCTCT	0.82
+	(1446, 1450)	GCR1	CTTCC	1.00
-	(1477, 1482)	SWI5	GGCTGG	0.83
-	(1478, 1489)	XBP1	TCGTCGAGGCTG	0.80
+	(1479, 1487)	repressor_of_CAR1	AGCCTCGAC	0.83
+	(1480, 1491)	XBP1	GCCTCGACGATG	0.87
